# Strategies to reduce antimicrobial resistance in newborns in low-income and middle-income countries: a systematic review and meta-analysis

**DOI:** 10.1016/S2214-109X(25)00533-9

**Published:** 2026-03-16

**Authors:** Rachel Lee Him, Davneet Sihota, Leila Harrison, Angela Dramowski, Susan E Coffin, Davidson H Hamer, Zulfiqar A Bhutta

**Affiliations:** a Centre for Global Child Health, The Hospital for Sick Children, Toronto, ON, Canada; b Department of Paediatrics and Child Health, Faculty of Medicine and Health Sciences, Stellenbosch University, Cape Town, South Africa; c Division of Infectious Diseases, Children's Hospital of Philadelphia, Philadelphia, PA, USA; d Department of Global Health, Boston University School of Public Health, Boston, MA, USA; e Section of Infectious Diseases, Department of Medicine, Boston University Avedisian & Chobanian School of Medicine, Boston, MA, USA; f Center on Emerging Infectious Diseases, Boston University, Boston, MA, USA; g Institute for Global Health and Development, The Aga Khan University South-Central Asia, East Africa, and UK, Karachi, Pakistan

## Abstract

**Background:**

Optimal strategies to reduce antimicrobial resistance (AMR) and their effect on newborns in low-income and middle-income countries (LMICs) remain unclear. We assessed the effectiveness of AMR mitigation strategies for newborn care in LMICs.

**Methods:**

A systematic review and meta-analysis was conducted. We searched MEDLINE, Embase, CINAHL, Global Index Medicus, Cochrane Central Register of Controlled Trials, and grey literature from Jan 1, 2000, to Nov 20, 2025, for randomised or quasi-randomised trials, quasi-experimental studies, observational or implementation studies, and programme evaluations. We included studies comparing any intervention, policy, or strategy designed to mitigate AMR development and spread (intervention) among newborns receiving facility-based or community-based care in LMICs (population), with standard practices or no intervention (comparator), on a range of outcomes including clinical and antibiotic use outcomes (outcome). Strategies to reduce AMR were categorised as regulation (structural or organisational actions), education (health-care worker trainings), or optimisation (responsible antimicrobial use). We pooled data from included studies to estimate the effectiveness of each of the three strategy types or a combination thereof. Given the low-resource context, we also narratively synthesised the available evidence on barriers and facilitators to implementing strategies to reduce AMR in newborn care settings (PROSPERO CRD42023388338).

**Findings:**

Of 3688 studies screened, 31 facility-based studies were included. Regulation reduced the risk of newborns receiving at least one antimicrobial by 21% (risk ratio 0·79 [95% CI 0·77–0·80]), but had no effect on neonatal sepsis risk. Optimisation reduced culture-positive sepsis risk by 32% (0·68 [0·55–0·83]) and risk of newborns on antibiotics by 13% (0·87 [0·78–0·98]), but had no effect on neonatal mortality risk. Regulation and optimisation did not significantly reduce neonatal mortality due to nosocomial bloodstream infection (BSI) risk (0·62 [0·31–1·25]). Regulation, education, and optimisation reduced neonatal mortality risk by 27% (0·73 [0·57–0·93]) and multidrug-resistant organism infections or colonisation risk by 29% (0·71 [0·52–0·97]). Regulation, education, and optimisation also decreased the risk of newborns receiving antibiotics by 29% (0·71 [0·61–0·81]) and the risk of duration of antibiotic therapy exceeding 5 days by 64% (0·36 [0·14–0·93]). Effect direction plots revealed overall positive directions of effect for outcomes including neonatal mortality (72·72%), neonatal mortality due to nosocomial BSI (100%), sepsis (75%), and drug-resistant (100%) and multidrug-resistant (80%) infection and colonisation. Effect direction plots also showed decreased overall antibiotic use (94·7%), access (71·4%) and watch (88·9%) antibiotic use, and duration of antibiotic therapy (83·3%) after strategies to reduce AMR were implemented. Common implementation barriers included delays in reporting culture test results, health-care worker non-adherence to infection prevention and control measures, and challenges in antibiotic prescribing for culture-negative newborns with sepsis-like presentation.

**Interpretation:**

To improve clinical outcomes, interventions targeting the control of antimicrobials alone will not suffice. Our results showed that three types of interventions (regulation, education, and optimisation) must be taken together to reduce AMR. These results can inform and accelerate guidance development for multi-dimensional, holistic, and integrated maternal and newborn care programmes in LMICs.

**Funding:**

The Bill & Melinda Gates Foundation.

## Introduction

Antimicrobial resistance (AMR) refers to the survival of micro-organisms including bacteria, viruses, fungi, and parasites despite treatment with antimicrobial drugs, rendering these life-saving medicines ineffective.^1^ The mortality and economic effects of AMR cannot be overstated. A recent global forecast of AMR burden in 2050 estimated 1·91 million deaths due to AMR and 8·22 million deaths associated with AMR.^2^ In addition to the immense pressure on global health systems, without urgent and coordinated action on AMR by 2050, the annual economic cost could reach US$1·2 trillion, forcing approximately 28·3 million people into extreme poverty.^3^

**Research in context**

**Evidence before this study**

Although antimicrobial resistance (AMR) is an urgent global challenge, it disproportionately affects low-income and middle-income countries (LMICs), necessitating effective mitigation strategies to reduce AMR transmission and infection events and improve newborn survival. However, to date, there have been no evidence syntheses on the effectiveness of strategies to reduce AMR in newborns in LMICs that have reported the full range of outcomes including mortality, morbidity, antimicrobial usage, and hospitalisation outcomes. Before conducting this review, we searched Ovid MEDLINE, Embase, CINAHL, Global Index Medicus, and the Cochrane Central Register of Controlled Trials for studies published between Jan 1, 2000, and Nov 15, 2022, without language restriction, using a search strategy that combined terms related to newborns, antimicrobial resistance, infections, and LMICs. Existing evidence syntheses were limited to a scoping review on antimicrobial stewardship in children aged 0–18 years and a systematic review on preterm newborns with gestational age ≤34 weeks. We identified one systematic review published in 2020 assessing the effectiveness of antimicrobial stewardship programmes in neonatology, but their analysis focused on hospitalised newborns and did not cover mitigation strategies beyond stewardship. All three reviews included studies from high-income country settings and meta-analyses were not conducted.

**Added value of this study**

To the best of our knowledge, we conducted the first systematic review and meta-analysis on strategies to reduce AMR in newborns receiving care in low-resource settings, describing and classifying a range of interventions implemented and evaluating their effects on a comprehensive set of clinical and process outcomes. Our review found that the most effective AMR mitigation strategies will create an enabling environment through organisational actions addressing AMR development and spread, regularly educate health-care workers on appropriate policies and procedures for AMR mitigation, and engage in antimicrobial stewardship for judicious prescribing. Implementing regulation, education, and optimisation interventions reduced the risks of clinical outcomes such as neonatal mortality by 27% and multidrug-resistant organism infections or colonisations by 29%, and reduced the risks of antimicrobial use outcomes such as the number of newborns receiving antibiotics by 29% and the duration of antibiotic therapy exceeding 5 days by 64%. Although some results did not reach statistical significance in meta-analysis, effect direction plots showed a pattern of predominantly positive health effects from studies implementing strategies to reduce AMR on outcomes of neonatal mortality due to nosocomial bloodstream infection, sepsis, early-onset sepsis (EOS; ≤72 h after birth), and late-onset sepsis (LOS; >72 h after birth). More successful implementation, which reduces both the direction and magnitude of effect, will involve addressing the most commonly reported barriers. We found substantial health-care facility and microbiology laboratory resource constraints, patterns of poor antibiotic prescribing, and frequent health-care worker non-adherence to infection prevention and control procedures. Studies emphasised tailoring AMR mitigation strategies to the local context, considering culture data and AMR trends, prescribing practices, availability of diagnostic testing, and staffing capacity. Notable evidence gaps include strategies designed for primary care facility and community settings; low-income country implementation; multi-site research; and evidence on water, sanitation, and hygiene, and vaccination interventions.

**Implications of all the available evidence**

This review highlights emerging approaches to prevent and control AMR in neonates hospitalised in resource-limited care settings. Our findings show that there are diverse opportunities to tailor-make local strategies to reduce AMR that incorporate all three critical and interdependent intervention types (regulation, education, and optimisation), given the broad range of interventions currently used in LMIC-based newborn care. For greater effects on clinical outcomes of neonatal mortality and morbidity, research funding and capacity building in this area are urgently needed. Special attention should be given to microbiology laboratory resources and capacity, as its proper functioning will heavily influence the ability of clinicians to make appropriate prescribing decisions.

An estimated 214 000 newborns die from sepsis caused by AMR pathogens every year, the greatest mortality burden occurring in low-income and middle-income countries (LMICs).^4^ Newborns in facility care settings are especially vulnerable to developing severe infections due to immature immunity, reliance on medical devices, and the need for surgical procedures. Antibiotics are the most frequently prescribed medication in the neonatal intensive care unit (NICU),^5^ and antibiotic stewardship must be monitored, as antibiotic misuse and overuse are key drivers of AMR.^6^, ^7^

Antimicrobial stewardship, also termed antimicrobial optimisation, refers to the appropriate use of antimicrobial agents through optimal drug selection, dosage, and duration of therapy.^1^ To facilitate antimicrobial stewardship, WHO's Access, Watch, or Reserve (AWaRe) classification distinguishes first and second choice antibiotics from antibiotics of last-resort for common paediatric infections.^8^, ^9^ Nevertheless, inadequate microbiology laboratory capacity to perform culture and susceptibility testing is a substantial challenge in LMICs, hindering clinicians’ ability to prescribe judiciously. In addition to effective and safe treatment, decreasing the risk of colonisation and infection with AMR organisms through water, sanitation, and hygiene (WASH) and infection prevention and control (IPC) measures are also essential.^10^

To the best of our knowledge, no comprehensive evidence synthesis existed on this topic at study initiation; therefore, we conducted a de novo systematic review to synthesise the evidence for strategies to reduce AMR in newborns in LMICs, and to assess implementation challenges, options, and evidence gaps.

## Methods

### Overview

Our review followed the Cochrane Handbook for Systematic Reviews of Interventions^11^ methods guidance, and is reported according to the PRISMA statement^12^ (appendix pp 2–7). Our protocol is registered in PROSPERO (CRD42023388338).

We searched Ovid MEDLINE, Embase, CINAHL, Global Index Medicus, and the Cochrane Central Register of Controlled Trials databases for fully published or in-press studies published in any language between Jan 1, 2000, and Nov 20, 2025 (appendix pp 8–19). We used search terms related to newborns, AMR, infections, and LMICs. We also searched grey literature and the reference lists of relevant reviews to identify other studies to include for screening.

Randomised or quasi-randomised trials, quasi-experimental studies, observational studies, programme evaluations, and implementation studies were eligible for inclusion. The exposure of interest was any intervention, policy, or strategy designed to promote antimicrobial stewardship or mitigate the development and spread of AMR. Interventions could occur in facility or community settings of LMICs. For example, interventions carried out by community health workers or in households or community pharmacies constituted community-based settings, and interventions carried out in primary, secondary, or tertiary and higher health-care facilities constituted facility-based settings. We defined LMICs according to the World Bank Group country classifications by income level at the time of the literature search.^13^ Eligible comparators included standard practices or no intervention. Primary outcomes of interest included all-cause neonatal mortality, infection-attributable neonatal mortality, stillbirth, early-onset sepsis, late-onset sepsis, localised infections (eg, omphalitis, urinary tract infection, and meningitis), localised infections due to multidrug-resistant organisms (MDROs), bloodstream infections (BSIs), BSIs due to MDROs, central-line-associated bloodstream infections, ventilator-associated pneumonias, and colonisation with MDROs (appendix pp 20–26). Laboratory-confirmed and clinically suspected localised and systemic infections were included but analysed as separate outcomes when possible. Secondary outcomes of interest included the proportion of newborns receiving any antibiotic, duration of antibiotic therapy, use of WHO AWaRe antimicrobials, and length of hospital stay (for inpatient newborns). These patient and process outcomes are common direct and proxy measures to evaluate the effectiveness of AMR reduction strategy implementation.^1^, ^14^ Study screening was completed independently and in duplicate by at least two reviewers (RLH and DS) using the same eligibility criteria. Screening conflicts were resolved through discussion and consensus or consultation with a third reviewer (LH). We excluded studies if they did not disaggregate data on newborns from the broader paediatric population, if their interventions focused on reducing infections without the intention of also affecting AMR, or if the study made ineligible comparisons such as comparing a high-income country (HIC) NICU with a LMIC NICU (appendix pp 27–30).

### Data abstraction and quality assessment

Data extraction and quality assessments were completed independently and in duplicate by two reviewers (RLH and DS). We extracted data in a purpose-built form generated in Microsoft Excel, which underwent a phase of piloting and modification. Extracted data consisted of publication and study characteristics such as design and methodology (including data collection and analysis); country, country income level, and setting details (eg, rural *vs* urban location, level of implementation, and type of facility); study population eligibility criteria and descriptions of participants (eg, baseline characteristics such as males *vs* females and very preterm, preterm, or term); and detailed descriptions of interventions, including types and duration and details on the comparison group, outcomes evaluated, and key conclusions. We also noted the presence or absence of funding and conflict of interest statements reported by included studies. All data were then matched, checked for consistency, and merged into a single form. Discrepancies were resolved through discussion and consensus by RLH, DS, and LH. When any study information was insufficient or unclear, we contacted the primary authors of the study. The methodological quality of included studies was assessed independently and in duplicate by two reviewers (RLH and DS) according to study design using the Cochrane RoB-2 tool for randomised controlled trials (RCTs),^15^ the Cochrane ROBINS-I tool for non-randomised studies,^16^ or the National Institutes of Health tool for observational studies.^17^

### Data synthesis and analysis

**Figure 1:**
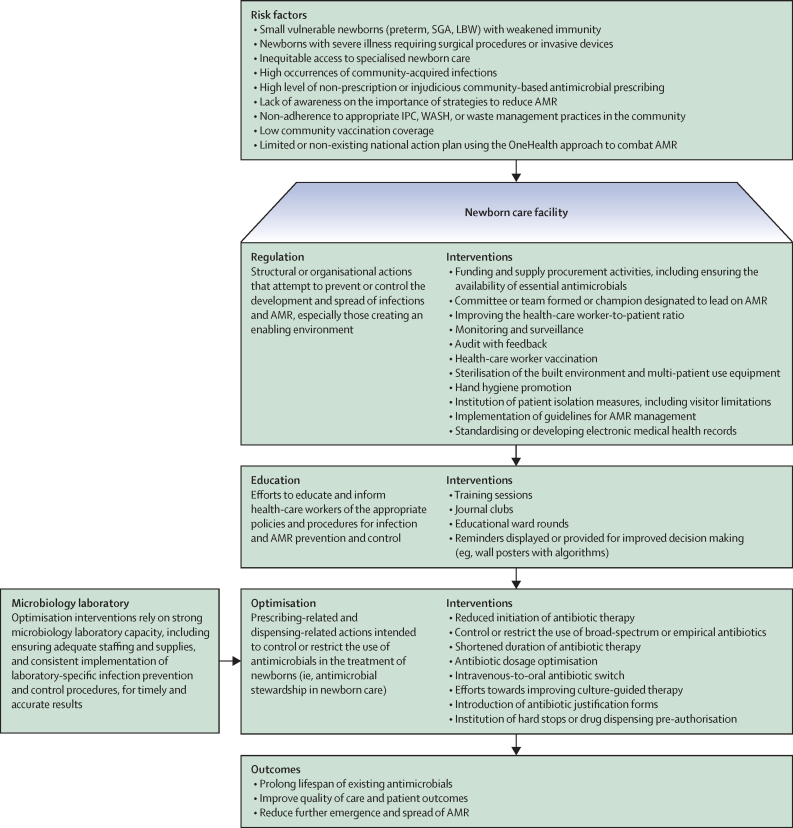
Conceptual framework for classification of interventions to reduce antimicrobial resistance as regulation, education, and optimisation AMR=antimicrobial resistance. IPC=infection prevention and control. LBW=low birthweight. SGA=small for gestational age. WASH=water, sanitation, and hygiene.

We classified AMR reduction strategies as either single-component or multi-component, and then categorised them as regulation, education, or optimisation interventions (figure 1). In developing this intervention classification framework, we consulted WHO's practical toolkit on antimicrobial stewardship programmes (ASPs) in LMIC-based health-care facilities,^18^ compiled a comprehensive list of prevention, detection, and mitigation actions against AMR, and grouped them by their overarching and most proximal intervention type. Categories consider structural or organisational actions targeting AMR management and surveillance (regulation); efforts towards health-care worker training (education); and efforts towards the judicious use of antimicrobials by clinicians (optimisation).

Meta-analysis data on strategies to reduce AMR contributed to a descriptive review on interventions to prevent and treat newborn infections in LMIC settings.^19^ We have since reframed intervention category terminology from restriction interventions to optimisation interventions, given the negative connotations associated with restricting use of antibiotics.

Meta-analyses were conducted and stored using Review Manager software.^20^ A minimum of two studies was required to conduct a meta-analysis, provided the studies could be meaningfully pooled. Anticipating clinical and methodological heterogeneity in the effect sizes between studies, we conducted random effects meta-analyses with inverse variance weighting. Pooled effect estimates are reported as risk ratios (RRs) for dichotomous outcomes and mean differences for continuous outcomes with associated 95% CIs. For assessing dichotomous outcomes, we chose to express RRs over risk differences for this effect measure's familiarity to health professionals and the general public, and for ease of interpretation. However, given that many of our included studies had uncontrolled before and after designs and expressing these results as risk differences might be helpful, we ran additional meta-analyses for a measure of absolute effect. For studies with repeated measurements (ie, point prevalence surveys), the most recent data were used for before and after intervention periods.

We conducted subgroup analyses by study design and level of care. Levels of care were defined as primary, secondary, and tertiary or higher. In instances where the level of care was not directly mentioned, we considered the description of the facilities’ human and material resources, infrastructure, and capacity for medical intervention. Primary care facilities were defined as health centres or outreach services for outpatient or ambulatory care. Secondary care facilities were hospitals located in districts or towns. Tertiary care facilities were hospitals in provincial or state capitals, usually affiliated with a teaching hospital and offering more sophisticated medical and surgical support. In assessing antibiotic usage, when possible, we performed subgroup analyses by classifying antibiotics or antibiotic classes into WHO's AWaRe antibiotics groups (appendix pp 31–32).

We conducted sensitivity analyses removing studies with a high risk of bias to assess the robustness of our effect estimates. We also evaluated the certainty of evidence for neonatal mortality outcomes using the GRADE criteria.^21^ If ten or more studies were included in the meta analysis, we investigated reporting biases (such as publication bias) using funnel plots and performed exploratory analyses to investigate any funnel plot asymmetry.

Acknowledging that meta-analysis alone might be inappropriate due to the heterogeneity in interventions and outcomes, we also conducted an evidence synthesis using effect direction plots.^22^, ^23^ These plots were generated to complement our meta-analysis by presenting the patterns in direction of effect as reported by included studies for each outcome. In an effect direction plot, up, down, and bidirectional arrows represent positive, negative, and conflicting or no health impacts, respectively. This synthesis method is based on vote counting, and neither statistical significance nor the magnitude of effect are considered, as per Cochrane guidance.^24^ Excluding studies with inconsistent effect direction (represented by bidirectional arrows), positive and negative effect direction arrows were counted and positive health effects were reported as a percentage of total positive and negative results.

We also performed a narrative synthesis thematically analysing barriers and facilitators to process and implementation, and grouping them according to the relevant WHO health system building blocks.^25^ We highlight evidence gaps and suggest future directions for effective AMR reduction strategy implementation and research.

### Role of the funding source

The funder of the study had no role in study design, data collection, data analysis, data interpretation, or writing of the report.

## Results

**Figure 2:**
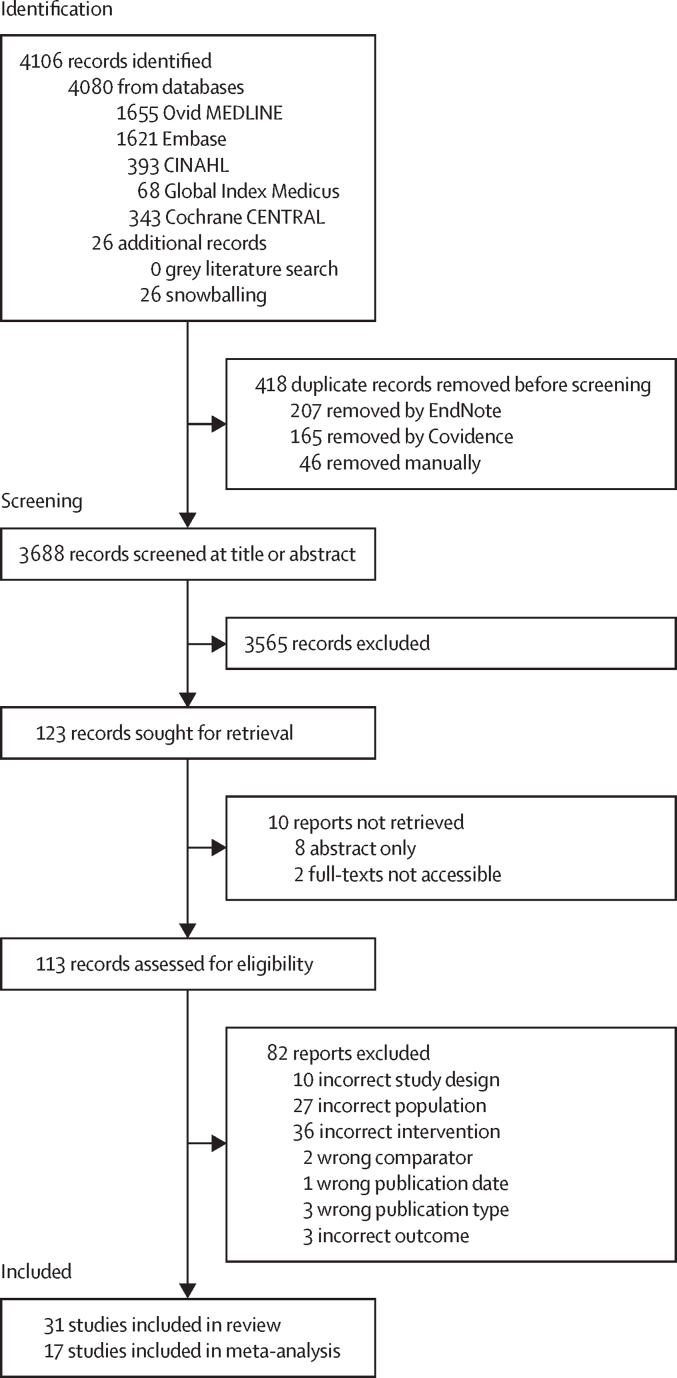
PRISMA flow diagram of studies in the review

**Figure 3:**
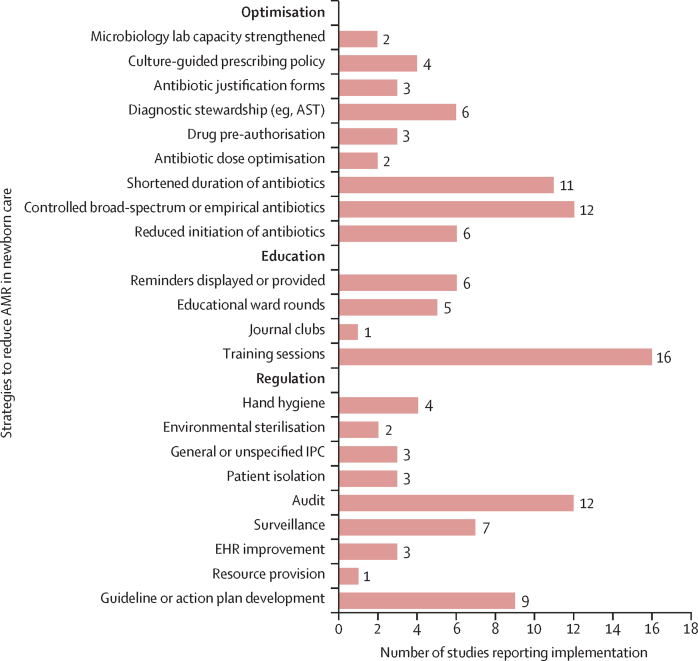
Number of studies reporting implementation of regulation, education, and optimisation interventions to reduce AMR in LMIC-based newborn care AMR=antimicrobial resistance. AST=antimicrobial susceptibility testing. EHR=electronic health record. IPC=infection prevention and control. LMIC=low-income and middle-income country.

Our literature search identified 4106 records. After removing 418 duplicates, 3688 records underwent title and abstract screening. Of these, 113 records were screened at full-text for eligibility against our pre-specified inclusion and exclusion criteria (figure 2). Ultimately, 31 studies were included in the review.^26^, ^27^, ^28^, ^29^, ^30^, ^31^, ^32^, ^33^, ^34^, ^35^, ^36^, ^37^, ^38^, ^39^, ^40^, ^41^, ^42^, ^43^, ^44^, ^45^, ^46^, ^47^, ^48^, ^49^, ^50^, ^51^, ^52^, ^53^, ^54^, ^55^, ^56^ Two studies (6·45%) focused exclusively on preterm (<37 weeks’ gestation) newborns^41^, ^49^ and one study (3·23%) exclusively on very low birthweight (<1500 g) newborns.^48^ Study duration ranged from 2 months^47^ to 5 years.^39^ One study took place in a low-income country^54^ (all others were conducted in upper-middle-income and lower-middle-income countries). No studies were conducted in community settings or solely in primary-care facilities (93·6% of studies were conducted in tertiary or higher care facilities; appendix pp 33–80). Ten studies (32·4%)^46^, ^35^, ^40^, ^42^, ^43^, ^45^, ^50^, ^51^, ^54^, ^56^ were single-component and 21 studies (67·7%)^26^, ^27^, ^28^, ^29^, ^30^, ^31^, ^32^, ^33^, ^34^, ^36^, ^37^, ^38^, ^39^, ^41^, ^44^, ^47^, ^48^, ^49^, ^52^, ^53^, ^55^ were multi-component. Among the ten single-component studies, three (30%)^46^, ^51^, ^56^ implemented regulation interventions, none (0%) implemented education interventions, and seven (70%)^35^, ^40^, ^42^, ^43^, ^45^, ^50^, ^54^ implemented optimisation interventions. Among the 21 multicomponent studies, three (14·3%)^27^, ^33^, ^47^ implemented regulation and education interventions, three (14·3%)^29^, ^37^, ^44^ implemented regulation and optimisation interventions, one (4·76%)^41^ implemented education and optimisation interventions, and 14 (66·7%)^26^, ^28^, ^30^, ^31^, ^32^, ^34^, ^36^, ^38^, ^39^, ^48^, ^49^, ^52^, ^53^, ^55^ implemented regulation, education, and optimisation interventions. The most frequent forms of regulation were audit (38·7%)^26^, ^27^, ^28^, ^30^, ^34^, ^36^, ^38^, ^39^, ^46^, ^47^, ^49^, ^52^ and guideline or policy development (29·0%; figure 3).^26^, ^28^, ^32^, ^34^, ^37^, ^39^, ^46^, ^48^, ^49^ Comparatively, IPC measures such as hand hygiene (12·9%),^27^, ^31^, ^33^, ^34^ environmental cleaning (6·45%),^33^, ^34^ and patient isolation (9·68%)^33^, ^44^, ^56^ were less frequently used. Health-care worker training sessions were the most common manner of education (51·6%),^27^, ^30^, ^31^, ^32^, ^33^, ^34^, ^36^, ^38^, ^39^, ^41^, ^47^, ^48^, ^49^, ^52^, ^53^, ^55^ and the predominant forms of optimisation were control of broad-spectrum or empirical antibiotic therapy (38·7%)^26^, ^30^, ^31^, ^35^, ^36^, ^37^, ^41^, ^43^, ^48^, ^49^, ^52^, ^54^ and shortened duration of antibiotic therapy (35·5%).^26^, ^32^, ^34^, ^36^, ^38^, ^39^, ^41^, ^49^, ^50^, ^52^, ^54^

Quality assessment of one RCT identified some risk of bias concerns.^45^ Quality assessment of 28 quasi-experimental studies identified six studies (21·4%) with a serious risk of bias^34^, ^36^ and 22 studies (78·6%) with a moderate risk of bias.^26^, ^27^, ^28^, ^29^, ^30^, ^31^, ^32^, ^33^, ^35^, ^37^, ^38^, ^39^, ^40^, ^42^, ^43^, ^49^ Quality assessment of two observational studies^40^, ^46^ determined them to be of fair quality (appendix pp 81–84). The GRADE certainty of evidence for three outcomes of neonatal mortality, including all-cause mortality and mortality due to nosocomial BSI, was very low (appendix pp 85–87).

Studies conducted pre-intervention and post-intervention comparisons, where pre-intervention involved usual newborn care and post-intervention involved newborn care with implemented strategies to reduce AMR (appendix pp 88–103).

Regulation interventions consisted of department antibiotic guidelines and a policy document developed by an antimicrobial stewardship committee, prospective audit with feedback,^46^ and weekly antibiotic surveillance rounds in the NICU.^51^ Regulation interventions did not significantly reduce the risk of neonatal sepsis or suspected sepsis (RR 1·35 [95% CI 0·52–3·46]; table).^46^, ^51^ The test for subgroup differences found a significant difference in neonatal sepsis or suspected sepsis by study design such that the risk was increased in a small observational study (2·40 [1·05–5·49])^46^ but a large quasi-experimental study showed a decrease that did not reach statistical significance (0·90 [0·79–1·03]).^51^ Of the two studies reporting sepsis, one did not report an outcome definition^51^ and the other defined sepsis as “sepsis, sepsis syndrome or septic shock with no clear anatomic site”.^46^ The reduction in risk of receiving at least one antimicrobial did not reach statistical significance (0·92 [0·64–1·31]).^46^, ^51^ However, the risk of receiving at least one antimicrobial was significantly reduced in the quasi-experimental study (0·79 [0·77–0·80]),^51^ an effect that was not observed in the observational study (1·13 [0·86–1·50]).^46^

**Table:** Results of meta-analysis for primary and secondary outcomes by strategy type

		**Number of studies (number of participants)**	**Effect estimate**	**Heterogeneity (*I*^2^)**	**p value**
**Single-component intervention: regulation**					
Neonatal sepsis or suspected sepsis (total)		2 (9269)	1·35 (0·52 to 3·46)	81%	0·54
	Observational	1 (38)	2·40 (1·05 to 5·49)	NA	0·020
	Quasi-experimental	1 (9231)	0·90 (0·79 to 1·03)	NA	..
Number of neonates receiving at least one antimicrobial (total)		2 (9269)	0·92 (0·64 to 1·31)	85%	0·64
	Observational	1 (38)	1·13 (0·86 to 1·50)	NA	0·010
	Quasi-experimental	1 (9231)	0·79 (0·77 to 0·80)	NA	..
**Single-component intervention: optimisation**					
All-cause neonatal mortality (total)		3 (1578)	1·12 (0·55 to 2·28)	0%	0·76
Culture-positive sepsis (total)		2 (1495)	0·68 (0·55 to 0·83)	0%	0·0002
Number of neonates on antibiotics (total)		3 (3947)	0·87 (0·78 to 0·98)	90%	0·020
AWaRe antibiotic usage (total)		3 (14 208)	0·82 (0·64 to 1·06)	97%	0·12
	Access	3 (4882)	0·81 (0·58 to 1·13)	98%	0·52
	Watch	3 (7831)	1·00 (0·61 to 1·63)	96%	..
	Mixed Access and Watch	2 (1495)	0·51 (0·16 to 1·57)	99%	..
**Multi-component intervention: regulation and optimisation**					
Neonatal mortality due to nosocomial bloodstream infection (total)		2 (1352)	0·62 (0·31 to 1·25)	0%	0·18
	Secondary	1 (273)	0·84 (0·05 to 13·37)	NA	0·82
	Tertiary or higher	1 (1079)	0·61 (0·30 to 1·25)	NA	..
**Multi-component intervention: regulation, education, and optimisation**					
All-cause neonatal mortality (total)		8 (28 928)	0·73 (0·57 to 0·93)	85%	0·010
All-cause neonatal mortality (sensitivity analysis; total)		7 (26 928)	0·73 (0·57 to 0·95)	87%	0·020
NEC (any Bell stage; total)		4 (22 585)	1·10 (0·95 to 1·27)	0%	0·22
NEC (Bell stage ≥ II; total)		3 (21 431)	1·13 (0·97 to 1·32)	0%	0·11
Neonatal sepsis (any; total)		4 (22 116)	0·46 (0·19 to 1·09)	95%	0·080
Late-onset sepsis (>72 h after birth; total)		3 (21 431)	0·64 (0·41 to 1·02)	56%	0·060
Culture-negative sepsis (total)		2 (21 245)	0·90 (0·81 to 0·99)	0%	0·040
Pneumonia (total)		2 (21 245)	0·88 (0·60 to 1·31)	91%	0·54
MDRO infections or colonisations (total)		2 (21 245)	0·71 (0·52 to 0·97)	0%	0·030
Bloodstream isolates of MRSA (total)		3 (1473)	1·96 (0·36 to 10·62)	0%	0·43
Bloodstream isolates of *Klebsiella* spp (total)		3 (1473)	0·68 (0·52 to 0·88)	0%	0·0040
Bloodstream isolates of *Acinetobacter* spp (total)		3 (1473)	1·03 (0·37 to 2·89)	1%	0·95
Bloodstream isolates of *Escherichia coli* (total)		3 (1473)	0·96 (0·15 to 6·11)	53%	0·96
Bloodstream isolates of *Enterobacter* spp (total)		3 (1473)	0·74 (0·30 to 1·85)	15%	0·52
Bloodstream isolates of CoNS (total)		3 (1473)	2·00 (1·08 to 3·73)	0%	0·030
Bloodstream isolates of *Pseudomonas* spp (total)		3 (1473)	0·25 (0·15 to 0·41)	0%	<0·0001
Bloodstream isolates of *Candida* spp (total)		2 (211)	0·15 (0·05 to 0·47)	0%	0·0010
Mean length of hospital stay in days (total)		2 (926)	7·42 (−13·37 to 28·20)	99%	0·48
Mean length of hospital stay in days (including Feng et al [2022]^30^*; total)		3 (8631)	4·92 (−6·34 to 16·18)	99%	0·39
Number of neonates receiving antibiotics (total)		5 (9863)	0·71 (0·61 to 0·81)	90%	<0·0001
Duration of antibiotic therapy >5 days (total)		2 (7891)	0·36 (0·14 to 0·93)	95%	0·030
Number of neonates with antibiotics discontinued after 48 h (total)		2 (13 673)	6·00 (0·75 to 47·88)	61%	0·090

AWaRe=Access, Watch, Reserve. CoNS=coagulase-negative *staphylococci*. MDRO=multidrug-resistant organism. MRSA=methicillin-resistant *Staphylococcus aureus*. NA=not applicable. NEC=necrotising enterocolitis.

* For Feng et al (2022),^30^ mean and SD were estimated from median (IQR), assuming a normal distribution, based on the given IQR, which appeared symmetrical around the median value.

No studies implemented education interventions without also incorporating regulation or optimisation.

Optimisation consisted of an antibiotic consumption protocol for empirical therapy of neonatal sepsis based on a review of NICU blood culture susceptibility data;^35^ an antibiotic stop policy;^50^ an antimicrobial justification form considering overall culture positivity rates, microbiological spectrum of organisms, and antimicrobial sensitivity;^42^ and an antibiotic stewardship protocol shifting from penicillin and first-generation and second-generation cephalosporins to more advanced antibiotics such as third-generation and fourth-generation cephalosporins, carbapenem, and glycopeptides.^43^

Optimisation interventions significantly reduced the risk of culture-positive sepsis by 32% (RR 0·68 [95% CI 0·55–0·83])^42^, ^43^ and the risk of receiving antibiotics by 13% (0·87 [0·78–0·98]),^35^, ^42^, ^43^ but had no significant effect on all-cause neonatal mortality (1·12 [0·55–2·28])^42^, ^43^, ^50^ or total antibiotics usage (0·82 [0·64–1·06]). The test for subgroup differences by the AWaRe antibiotics classification was not significant, and available data on reserve group antibiotics was not poolable, as only one study reported on use.^35^ No subgroup analyses by study design were conducted as all studies were quasi-experimental.

Three studies^27^, ^33^, ^47^ implemented regulation and education interventions; however, meta-analysis was not possible due to incomparable outcomes or outcome metrics.

Regulation interventions involved the application of a culture-based antibiotic policy and infection control measures, and the institution of an infection control checklist covering disinfection, cleaning, hand hygiene, and visitors.^29^ Regulation also included an infection control programme introducing the clustering of nursing care such that patients, rather than duties, were allocated; setting limits for invasive care procedures such as peripheral venous catheters and nasogastric feeding tubes to minimise the development of nosocomial BSI; and promoting the early discharge of neonates.^37^ Optimisation interventions included specifying ampicillin and cefotaxime as first-line therapy for early-onset sepsis (≤72 h after birth), vancomycin and imipenem for late-onset sepsis (>72 h after birth), and piperacillin-tazobactam and linezolid as second line therapy.^29^ Optimisation also included establishing an algorithm for empirical therapy of suspected early-onset sepsis.^37^ Regulation and optimisation interventions did not significantly reduce neonatal mortality risk due to nosocomial BSI (RR 0·62 [95% CI 0·31–1·25]).^29^, ^37^ In a subgroup analysis by level of care, the test for subgroup differences did not reach statistical significance. No subgroup analyses by study design were conducted as all studies were quasi-experimental.

One study^41^ implemented education and optimisation interventions; meta-analysis was therefore not possible.

Regulation consisted of high-level structural and organisational interventions such as hospital guideline or policy development, AMR surveillance, audit with feedback, and IPC measures. Specifically, interventions included creating a unit protocol for sepsis management and antibiotic prescribing;^26^ applying new guidelines to reduce infection rates, decrease in-hospital drug costs, and reduce length of hospital stay in the surgical NICU;^28^ guidelines for diagnosing and treating early-onset sepsis and late-onset sepsis;^48^ prospective audits with feedback^26^, ^28^, ^30^, ^34^, ^38^, ^39^, ^49^ and recording clinical manifestations on an observation form for neonatal infections;^30^ introducing drug-resistant pathogen surveillance and infection control measures with ethanol hand rub provision;^31^ introducing a department policy plan for starting antibiotics and adoption of universal aseptic precautionary measures;^34^ revising the antibiotic policy for stricter management and instituting a no-prick policy for asymptomatic and stable preterm newborns;^49^ forming a multidisciplinary team to monitor antibiotic use, conduct patient chart reviews and other oversight responsibilities; establishing guideline-based algorithms to direct treatment; and transitioning to an electronic health record system for more accurate drug administration and dispensing data.^39^

Education involved training sessions, educational rounds, and staff reminders. Specifically, these included training providers on appropriate diagnosis and treatment of sepsis,^48^ training on sepsis guidelines and appropriate antibiotic use,^30^, ^38^, ^39^ training through presentations and the display of reminder posters,^26^ training on good sampling to avoid the poly-microbial nature of the samples,^28^ preparatory workshops consisting of lectures on hand hygiene and infection control and interactive case discussions,^31^ confidence and team building sessions for preventing sepsis in the NICU,^34^ a lecture on antibiotic stewardship, weekly bedside rounds with case-based discussions, installation of a dashboard indicator displaying the number of infants receiving antibiotics with stopping orders,^49^ and ASP rounds and daily best practice alert messages sent to the prescribing team.^39^

Optimisation consisted of antimicrobial stewardship interventions for judicious drug prescribing and dispensing. Specifically, these included establishing checkpoints for starting and early stoppage of antibiotics, formulating a specific protocol to start vancomycin, and reviewing the yearly antibiotic policy as per antibiogram;^26^ introducing an ASP to control and limit antibiotic misuse^28^ or restrict use of empirical antibiotics;^48^ instituting preliminary reporting of direct gram-stained films before final reporting of sensitivity results and automation to decrease turnaround time of laboratory investigations;^28^ diagnostic stewardship measures involving antibiotic susceptibility testing with quality control procedures^31^ and early tracing of preliminary blood culture reports by 48 h;^49^ creating daily or monthly checklists asking staff to re-evaluate empirical antibiotic therapy;^28^, ^31^ ensuring empirical antibiotic therapy of no more than 48 h for suspected infections and five days for pneumonia and culture-negative sepsis^30^, ^38^ or, specifically, a hard-stop at 48 h for antibiotics prescribed for suspected early-onset sepsis;^39^ discontinuing antibiotics at 72 h and streamlining and de-escalation of antibiotics;^34^ de-escalating to the narrowest spectrum effective antibiotic in blood culture-positive cases;^49^ and implementing point-of-prescription interventions including drug pre-authorisation.^34^, ^38^

Regulation, education, and optimisation interventions significantly reduced the risk of all-cause neonatal mortality by 27% (RR 0·73 [95% CI 0·57–0·93]).^26^, ^28^, ^30^, ^31^, ^34^, ^38^, ^48^, ^49^ Sensitivity analysis in which we removed one study^34^ with a high risk of bias revealed no change in the estimated risk of neonatal mortality. Regulation, education, and optimisation also significantly reduced the risks of MDRO infection or colonisation by 29% (0·71 [0·52–0·97]),^30^, ^38^ culture-negative sepsis by 10% (0·90 [0·81–0·99]),^30^, ^38^ and culture-identified bloodstream isolates of *Candida* spp by 85% (0·15 [0·05–0·47]),^28^, ^49^ *Pseudomonas* spp by 75% (0·25 [0·15–0·41]),^28^, ^31^, ^49^ and *Klebsiella* spp by 32% (0·69 [0·52–0·88]).^28^, ^31^, ^49^ The risk of bloodstream isolates of coagulase-negative staphylococci increased (2·00 [1·08–3·73]),^28^, ^31^, ^49^ but there was no significant difference in the risks of methicillin-resistant *Staphylococcus aureus* (MRSA; 1·96 [0·36–10·62]),^28^, ^31^, ^49^ *Acinetobacter* spp (1·03 [0·37–2·89]),^28^, ^31^, ^49^ *Escherichia coli* (0·96 [0·15–6·11]),^28^, ^31^, ^49^ or *Enterobacter* spp (0·74 [0·30–1·85]).^28^, ^31^, ^49^ The effect of regulation, education, and optimisation did not reach statistical significance for outcomes of necrotising enterocolitis,^26^, ^30^, ^38^, ^48^ neonatal sepsis^30^, ^38^, ^39^, ^48^ (see forest plot footnotes for sepsis definitions; appendix p 95), late-onset sepsis,^30^, ^38^, ^48^ or pneumonia.^30^, ^38^

Implementing regulation, education, and optimisation interventions reduced the risk of receiving antibiotics by 29% (RR 0·71 [95% CI 0·61–0·81]).^26^, ^30^, ^39^, ^48^, ^49^ The risk of antibiotic therapy duration >5 days was also significantly reduced by 64% (0·36 [0·14–0·93]).^30^, ^48^ The risk of antibiotic therapy discontinuation after 48 h did not reach statistical significance (6·00 [0·75–47·88]).^38^, ^49^ There was also no significant difference in mean length of hospital stay (mean difference 7·42 days [95% CI –13·37 to 28·20]).^28^, ^39^ In a second meta-analysis of mean length of hospital stay, we included one large study^30^ reporting median length of hospital stay, with an IQR symmetrical around the median value. We could reasonably assume a normal distribution and calculated mean (SD) from the reported median (IQR), which confirmed no significant difference in length of hospital stay (mean difference 4·92 days [–6·34 to 16·18]). One other study^38^ reported median length of hospital stay but we could not reasonably assume a symmetrical distribution and the study was therefore not included in the meta-analysis. For studies implementing regulation, education, and optimisation interventions, no subgroup analyses by study design were conducted as all studies were quasi-experimental.

Forest plots for dichotomous outcomes expressed as risk differences can be found in the appendix (pp 104–119). Publication bias was not assessed as there were fewer than ten studies pooled in any single meta-analysis, so there was not enough power to detect bias.

**Figure 4:**
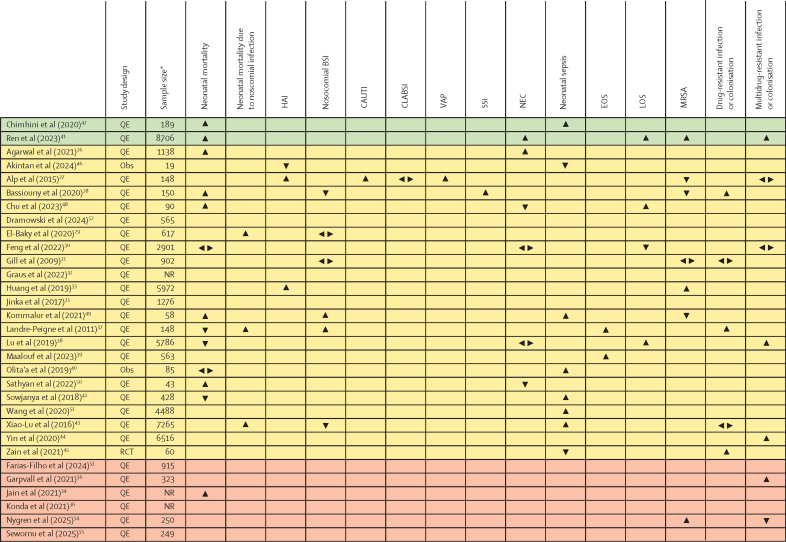
Effect direction plot summarising the effect of strategies to reduce AMR on primary outcomes of mortality and morbidity Upward arrows indicate a positive health effect (improvement), downward arrows indicate a negative health effect (deterioration), sideways arrows indicate no change, mixed effects, or conflicting findings. Study quality is denoted by row colour: green indicates low risk of bias, orange indicates some concerns, and red indicates high risk of bias. AMR=antimicrobial resistance. BSI=bloodstream infection. CAUTI=catheter-associated urinary tract infection. CLABSI=central line-associated bloodstream infection. EOS=early-onset sepsis. HAI=health-care-associated infection. LOS=late-onset sepsis. MRSA=methicillin-resistant *Staphylococcus aureus*. NEC=necrotising enterocolitis. NR=not reported. Obs=observational study. QE=quasi-experimental study. RCT=randomised controlled trial. SSI=surgical site infection. VAP=ventilator-assisted pneumonia. *Sample size represents the number of individuals in the intervention or exposed group.

Because meta-analysis was not possible for many intervention types and outcomes, we employed effect direction plots to summarise the impact of strategies to reduce AMR on primary outcomes (figure 4) and secondary outcomes (appendix pp 120–121). Excluding studies with inconsistent effect direction, there were predominantly positive health effects as a percentage of total positive and negative results for primary outcomes including all-cause neonatal mortality (eight [72·7%] of 11 studies), neonatal mortality due to nosocomial BSI (three [100·0%] of three studies), hospital-associated infections (two [66·7%] of three studies), neonatal sepsis (six [75·0%] of eight studies), early-onset sepsis (two [100·0%] of two studies), late-onset sepsis (three [75·0%] of four studies), drug-resistant infections or colonisations (three [100·0%] of three studies), and multidrug-resistant infections or colonisations (four [80·0%] of five studies). Only 50% improvement was observed for outcomes of nosocomial BSI (two of four studies), necrotising enterocolitis (two of four studies), and MRSA (three of six studies). A very small number of studies reported on catheter-associated urinary tract infections, central-line-associated bloodstream infections, ventilator-associated pneumonias, and surgical site infections. Among secondary outcomes, there were predominantly positive health effects as a percentage of total positive and negative results for overall antibiotic use (18 [94·7%] of 19 studies), access antibiotic group use (five [71·4%] of seven studies), watch antibiotic group use (eight [88·9%] of nine studies), and duration of antibiotic use (five [83·3%] of six studies). There was a very small number of studies reporting on reserve antibiotic group use, and only four (40·0%) of ten studies showed decreased lengths of hospital stay after strategies to reduce AMR were implemented.

**Figure 5:**
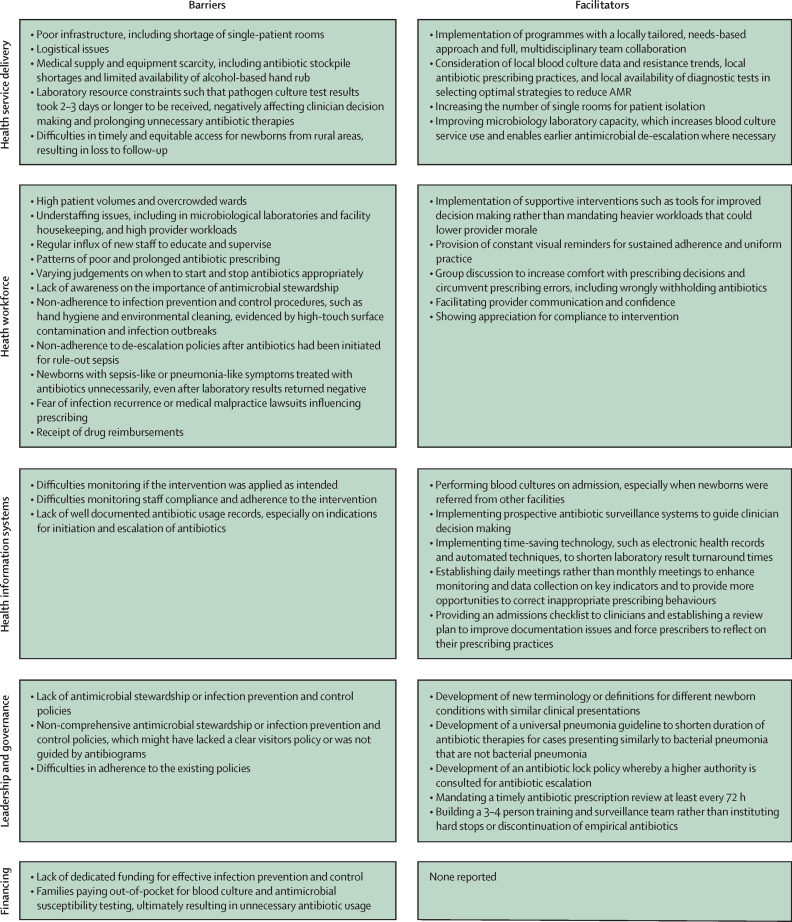
Barriers and facilitators to effective process and implementation of strategies to reduce AMR in LMIC-based newborn care, by WHO health system building blocks AMR=antimicrobial resistance. LMIC=low-income and middle-income country.

Barriers and facilitators to effective process and implementation of strategies to reduce AMR in LMIC-based newborn care by WHO health system building blocks of health service delivery, health workforce, health information systems, leadership and governance, and financing are detailed (figure 5).^25^ The most frequently reported barriers were delayed culture results, regular influxes of new staff requiring training and oversight, non-adherence to IPC procedures, and unnecessary antibiotic treatment among culture-negative newborns with sepsis-like symptoms. The most frequently reported facilitators were locally tailored approaches to AMR mitigation, constant visual reminders for staff, and admissions checklists to improve documentation. No studies reported on barriers or facilitators to procurement, distribution, or regulatory control of essential medicines.

## Discussion

To the best of our knowledge, this is the first evidence synthesis on strategies to reduce AMR in newborn care specifically concerning LMICs. We found that multi-component interventions incorporating regulation, education, and optimisation were most effective in improving newborn survival, with little evidence of adverse patient outcomes attributable to these interventions. Regulation, education, and optimisation interventions reduced the risk of all-cause neonatal mortality by 27%, culture-negative sepsis by 10%, MDRO colonisation and infection by 29%, and bloodstream isolates of common pathogens by 85% (*Candida* spp), 75% (*Pseudomonas* spp), and 32% (*Klebsiella* spp). Regulation, education, and optimisation interventions also reduced the risk of newborns receiving antibiotics by 29% and duration of antibiotic therapy >5 days by 64%. Although the risk of bloodstream isolates of coagulase-negative staphylococci was twice as likely, there was no significant difference in mean length of hospital stay pre-intervention versus post-intervention. Effect direction plots indicated that studies predominantly reported a positive effect for outcomes of neonatal mortality, neonatal mortality due to nosocomial BSI, neonatal sepsis, drug-resistant and multidrug-resistant colonisation and infection, overall antibiotic use, access and watch group antibiotic use, and duration of antibiotic use, after strategies to reduce AMR were implemented.

Our review synthesises the best and most up-to-date evidence on strategies to reduce AMR in newborns living in LMICs. We quantified the effect of these interventions for a comprehensive set of outcomes including mortality, morbidities, antibiotic use, and length of hospitalisation using meta-analysis. Additionally, we used an alternative synthesis method to show patterns of effect direction by outcome to elucidate individual study findings, especially those that were unable to be pooled in meta-analysis or might have had a non-statistically significant yet positive impact in overall direction of effect. Finally, our examination of the reported barriers and facilitators to effective process and implementation in LMICs indicate the urgent considerations needed to produce greater magnitude effects going forward.

However, there are several limitations that should be considered. First, in most meta-analyses, there was substantial to considerable levels of heterogeneity, probably due to the variation in interventions implemented and differences in sample sizes, regional contexts, and compliance and adherence to the interventions. Second, since the inconsistency in outcomes and outcome measures limited the meta-analyses possible, supplementary effect direction plots offered a clear visualisation of effect direction patterns. However, it is important to note that the effect direction plot does not account for statistical significance, magnitude of effect, or the relative sizes of studies.^24^ Third, few of the included studies were from low-income countries, which carry the greatest AMR burden, and most studies were single site, with 94% of studies conducted at the tertiary or higher level. Although we might attribute the greater proportion of tertiary-level studies to serious cases of suspected sepsis referred to facilities better equipped to support advanced care, these limitations might affect the generalisability of our findings to lower levels of the health-care system and the lowest-resourced countries among LMICs. Our review also found no evidence for community-based strategies to reduce AMR, although it has been well documented that antibiotics are frequently dispensed without a prescription in the community.^57^, ^58^, ^59^ Fourth, our review is limited by an absence of data from high-quality RCTs, given the practical and ethical challenges associated with this study design. Included studies also reported sparse narrative details on barriers and facilitators to implementation, highlighting the need for high-quality and mixed-method RCTs to be conducted in the future for a more reliable and extensive analysis of newborn care-specific challenges. Fifth, the presence of outborn neonates in included studies might suggest an underestimation of antibiotic usage, as empirical treatment could have occurred before arrival at the study facility. Finally, there was limited data collection and reporting on AWaRe antibiotic usage, especially of reserve antibiotics, and on drug-resistant organisms isolated from cultures, which might be indicative of the lack of antimicrobial surveillance and laboratory capacity observed in LMICs.

Previous systematic reviews, including one with global data from 114 countries on governance efforts to control AMR,^60^ found substantial variation in antiresistance interventions used.^60^, ^61^, ^62^ Similar reviews examining the evidence base for implementing ASPs in paediatric,^63^ newborn,^14^ and preterm newborn^61^ populations found that ASPs reduced inappropriate prescribing in children (aged 0–18 years) with mixed effects on antibiotic resistance, but more than half of the data originated from the USA alone.^63^ Further, ASPs reduced antibiotic consumption in hospitalised newborns, including newborns from HICs.^14^ In preterm newborns, ASPs targeting reduced initiation and shortened duration of antibiotic therapy appeared more effective than other organisational actions in reducing antibiotic consumption.^61^ Nevertheless, the review favoured multimodal interventions over unimodal ones, and further emphasised the importance of specific interventions over general ones.^61^ A recent meta-analysis on ASPs in neonates, including HIC data, found reduced initiation and duration of antibiotic therapy with no increase in adverse events.^62^ In contrast, our review focused on a broader range of AMR mitigation efforts (including IPC) and is aimed at supporting implementation in LMIC settings, where the infectious disease burden is greatest. Our review also provides a conceptual framework for classification of interventions into three overarching categories, which can help narrow down the set of interventions to carry out. For example, our findings suggest that implementing at least one intervention of regulation, education, and optimisation each might be more beneficial than implementing multiple kinds of regulation interventions alone. Notably, these three categories are interdependent; high-level organisational actions (regulation) require training prescribers (education) to implement the appropriate policies and procedures at the patient care level (optimisation). Thus, our review finds that all three intervention types are essential to a successful AMR reduction programme.

The barriers and facilitators identified in our review are consistent with the findings of other studies.^64^, ^65^ A scoping review evaluating barriers and facilitators to ASP implementation in LMICs found that 50% of studies reporting on barriers identified a shortage of human resources, lack of leadership, and unreliable laboratory support and infrastructure.^64^ The most common enablers to ASP implementation included establishing guidelines based on WHO's AWaRe classification and a dedicated ASP committee.^64^ Another review evaluating behaviour change interventions for improved antibiotic use in LMICs found that providers were most often challenged by resource and infrastructure constraints, even in tertiary care facilities.^65^ Providers also identified lack of national initiatives on antibiotic use and difficulties changing prescribing patterns as barriers.^65^ When interventions were well integrated into routine practice and when higher-level stakeholders were committed and engaged in the success of the ASP, providers felt enabled.^65^ Similarly, a review on the drivers of AMR in LMICs found that national action plans were hampered by lack of political will, insufficient funding, weak surveillance structures, lack of technical capacity, lack of provider training, and inadequate and inequitable access.^66^

To achieve our global target of 10% reduction in mortality attributable to AMR by 2030,^67^ policy makers and decision makers should consider that the estimated cost per capita of implementing a health-care facility-based package to address AMR is $1·40–9·40 adjusting for purchasing power parity. The return on investment is considerable at 4·7 times higher than the implementation costs, resulting in reduced strain on hospital resources, increased savings on health-care expenditures, and future gains in workforce productivity.^68^ Investment in strategies to combat AMR should prioritise prevention, scaling up coverage of interventions such as IPC, WASH, and vaccination, which are currently neglected. A recent modelling analysis showed that targeting these areas could have the greatest potential for reducing bacterial AMR in high-burden, low-resource settings.^69^

Furthermore, clinical interventions and ongoing education are unlikely to achieve population-level impacts unless the socioeconomic determinants of health are addressed.^70^ Although few studies examined health-care access or affordability, difficulties in patient follow-up and antibiotic shortages were noted, and these can also fuel AMR. Just as multifaceted interventions were more effective than any single intervention, the multisectoral One Health approach to antimicrobial stewardship and IPC is largely recognised to combat the development of AMR on multiple fronts,^4^, ^6^, ^66^ and efforts to incorporate such policies in LMICs are highly recommended, as highlighted in the recent UN General Assembly's Political Declaration on AMR.^71^

Our review reinforces that scaling up and sustaining AMR mitigation efforts will require overcoming substantial resource, infrastructure, capacity, and logistical constraints. These systemic challenges emphasise the urgent need for an integrated, whole-system approach of continuous cross-programme and inter-organisational coordination, collaboration, and shared knowledge in planning, implementation, and scale-up of strategies to reduce AMR. This approach will promote transparency, trust, and efficiency at all levels of the system, national to local.^72^

As laboratory capacity is often severely restricted by staff and supply shortages, delayed or absent culture results mean clinicians must prescribe based on clinical judgment alone when presented with the non-specific signs and symptoms of sepsis. For treatment to be safe and effective, there must first be consistent availability of blood culture bottles, culture media, supplies for manual or automated identification of pathogens, susceptibility testing, and prompt reporting of laboratory results to clinicians. For NICUs with high blood culture contamination rates, strengthening protocols in skin antisepsis, disinfection of bottle caps, and correct phlebotomy technique are necessary.^55^ When resources permit, collection of paired blood samples will improve accuracy in diagnosing true BSIs.^55^

Families of newborns found blood cultures unaffordable^46^, ^55^ or unavailable,^73^ indicating advancements in low-cost rapid diagnostic testing are urgently needed.^46^ Adoption of so-called leapfrog solutions have been proposed to help address these needs in LMICs.^72^, ^74^ Such innovations are rapid and low-maintenance, resilient against environmental stressors such as heat and humidity, and require minimal infrastructure and previous expertise.^75^ For example, prioritising the development of rapid, accurate, and affordable point-of-care testing over slower culture-based methods will help in contexts where specimens require transportation to a microbiology laboratory.^74^

Studies in this review also emphasised government prioritisation, investment in AMR reduction and IPC programmes, and high-level local leadership support as essential for health-care culture change.^27^, ^34^, ^39^ More effective re-allocation of existing resources at a system-wide level is needed;^27^ one study highlights nursing capacity and spatial capacity as vital to the success of their intervention.^47^ ASP implementation was more successful when facilities were not required to add extra cost, manpower, or equipment towards the programme,^26^ but rather used existing platforms such as ward rounds and weekly unit meetings.^47^ Proactive antibiotic management should be coupled with consistent, robust IPC practices.^35^, ^36^, ^46^, ^52^ Intervention bundles should consider including single-room isolation for high-risk newborns, especially to target NICUs with high MRSA transmission.^33^

Interventions requiring a behaviour change might only produce short-term improvement in compliance. For sustained improvement, clear and consistent messaging,^47^ continuous enforcement,^34^, ^46^ and more innovative methods should be considered to challenge previous beliefs and the overall prescribing culture.^27^, ^32^ Regular training and feedback sessions pose demands on the health-care system but effectively keep the team engaged in the long-term.^34^ Compulsory approval by senior staff or clinical managers for antibiotic continuation and escalation might benefit NICUs with a regular influx of inexperienced providers lacking the confidence to discontinue antibiotics for culture-negative sepsis.^34^, ^36^, ^41^ Audit with feedback was deemed particularly effective for clinicians to gauge their prescribing performance.^47^

Robust, multi-site studies with larger sample sizes^45^ and longer-term follow-ups are needed to track the effect of antimicrobial stewardship and IPC interventions on the emergence of genomic-level changes resulting in the development of bacterial resistance.^28^, ^43^ Many studies found the quality improvement approach simple, practical, and effective but lacking in the literature, and recommended their use in future research.^34^, ^36^, ^49^ Studies should consider the usefulness and accuracy of the antibiotic consumption metric used for neonatal and paediatric populations. For example, defined daily dose requires an assumption of average bodyweight, which varies widely in a paediatric population, although many included studies expressed antibiotic consumption using this metric.^35^, ^51^ Variation in antibiotic consumption metrics limited the comparison of findings across studies.

Our review found that strategies incorporating regulation, education, and optimisation interventions were most effective at reducing the risk of neonatal mortality and MDRO colonisation and infection. The risk of overall antibiotic usage and antibiotic therapy durations of >5 days was also reduced, without increasing mean length of hospitalisation. Our findings show that implementing these three interdependent strategy types is feasible even for LMIC contexts, as selections can be made from a diverse range of interventions within each intervention category. Although meta-analysis found that risk reductions for some outcomes did not reach statistical significance, the effect direction for most outcomes showed that positive heath impacts are still being achieved at some magnitude. Much greater progress can be made by addressing the barriers faced in these settings and filling the evidence gaps identified by our review. More robust, multi-site LMIC-based research, especially from low-income countries, is needed, with attention to adopting a standardised antibiotic consumption metric to facilitate comparison of intervention effectiveness in newborn care. We identified serious systemic challenges which necessitate a whole-system approach, with immense need for improvement in microbiology laboratory capacity strengthening. Immediate investment in preventive measures and capacity building will enable us to achieve our global target on AMR by 2030 and make greater strides in advancing newborn health and survival in LMICs.

### Contributors

### Data sharing

The data presented in this study are available in supplementary documents.

## Declaration of interests

DHH and SEC received funding for salary support and a technical advisory planning meeting from the Bill & Melinda Gates Foundation. SEC received funding for salary support and a study of neonatal sepsis in Botswana from the Centres for Disease Control and Prevention. All other authors declare no competing interests.

## References

[1] WHO (2019).

[2] Naghavi M, Vollset SE, Ikuta KS (2024). Global burden of bacterial antimicrobial resistance 1990–2021: a systematic analysis with forecasts to 2050. Lancet.

[3] World Bank (2017).

[4] Laxminarayan R, Matsoso P, Pant S (2016). Access to effective antimicrobials: a worldwide challenge. Lancet.

[5] Prusakov P, Goff DA, Wozniak PS (2021). A global point prevalence survey of antimicrobial use in neonatal intensive care units: the no-more-antibiotics and resistance (NO-MAS-R) study. EClinicalMedicine.

[6] Sulis G, Sayood S, Gandra S (2022). Antimicrobial resistance in low- and middle-income countries: current status and future directions. Expert Rev Anti Infect Ther.

[7] Costelloe C, Metcalfe C, Lovering A, Mant D, Hay AD (2010). Effect of antibiotic prescribing in primary care on antimicrobial resistance in individual patients: systematic review and meta-analysis. BMJ.

[8] Hsia Y, Lee BR, Versporten A (2019). Use of the WHO Access, Watch, and Reserve classification to define patterns of hospital antibiotic use (AWaRe): an analysis of paediatric survey data from 56 countries. Lancet Glob Health.

[9] WHO (2021).

[10] Dramowski A, Aucamp M, Beales E (2022). Healthcare-associated infection prevention interventions for neonates in resource-limited settings. Front Pediatr.

[11] Higgins JPT, Thomas J, Chandler J Cochrane Handbook for Systematic Reviews of Interventions version 6.5 (updated August 2024). https://www.cochrane.org/handbook2024.

[12] Page MJ, McKenzie JE, Bossuyt PM (2021). The PRISMA 2020 statement: an updated guideline for reporting systematic reviews. BMJ.

[13] Hamadeh N, Van Rompaey C, Metreau E (2024). World Bank Group country classifications by income level for FY24. https://blogs.worldbank.org/en/opendata/new-world-bank-group-country-classifications-income-level-fy24.

[14] Araujo da Silva AR, Marques A, Di Biase C (2020). Effectiveness of antimicrobial stewardship programmes in neonatology: a systematic review. Arch Dis Child.

[15] Sterne JAC, Savović J, Page MJ (2019). RoB 2: a revised tool for assessing risk of bias in randomised trials. BMJ.

[16] Sterne JA, Hernán MA, Reeves BC (2016). ROBINS-I: a tool for assessing risk of bias in non-randomised studies of interventions. BMJ.

[17] National Institutes of Health, National Heart, Lung, and Blood Institute (2021). Study quality assessment tools. https://www.nhlbi.nih.gov/health-topics/study-quality-assessment-tools.

[18] WHO (2019).

[19] Lee Him R, Rehman S, Sihota D (2024). Prevention and treatment of neonatal infections in facility and community settings of low- and middle-income countries: a descriptive review. Neonatology.

[20] Cochrane (2020).

[21] GRADEpro (2024).

[22] Boon MH, Thomson H (2021). The effect direction plot revisited: application of the 2019 Cochrane Handbook guidance on alternative synthesis methods. Res Synth Methods.

[23] Thomson HJ, Thomas S (2013). The effect direction plot: visual display of non-standardised effects across multiple outcome domains. Res Synth Methods.

[24] McKenzie JE, Brennan SE (2024). Synthesizing and presenting findings using other methods. https://www.training.cochrane.org/handbook.

[25] WHO (2010).

[26] Agarwal S, Patodia J, Mittal J, Singh Y, Agnihotri V, Sharma V (2021). Antibiotic stewardship in a tertiary care NICU of northern India: a quality improvement initiative. BMJ Open Qual.

[27] Alp E, Orhan T, Kürkcü CA, Ersoy S, McLaws ML (2015). The first six years of surveillance in pediatric and neonatal intensive care units in Turkey. Antimicrob Resist Infect Control.

[28] Bassiouny DM, Hassan RM, Shalaby A, Halim MMA, Wassef MA (2020). Establishment of an antimicrobial stewardship strategy on the surgical NICU at Cairo University specialized pediatric hospital. J Pediatr Surg.

[29] El-Baky RMA, Senosy EM, Omara W, Mohamed DS, Ibrahim RA (2020). The impact of the implementation of culture-based antibiotic policy on the incidence of nosocomial infections in neonates hospitalized in neonatal intensive care unit in a general Egyptian hospital in upper Egypt, 2016–2018. J Pure Appl Microbiol.

[30] Feng K, He Y, Liu W, Zhang X, Song P, Hua Z (2023). Evaluation of antibiotic stewardship among near-term and term infants admitted to a neonatal unit. Eur J Pediatr.

[31] Gill CJ, Mantaring JB, Macleod WB (2009). Impact of enhanced infection control at 2 neonatal intensive care units in the Philippines. Clin Infect Dis.

[32] Graus JM, Herbozo C, Hernandez R, Pantoja AF, Zegarra J (2022). Managing antibiotics wisely in a neonatal intensive care unit in a low resource setting. J Perinatol.

[33] Huang H, Ran J, Yang J, Li P, Zhuang G (2019). Impact of MRSA transmission and infection in a neonatal intensive care unit in China: a bundle intervention study during 2014–2017. BioMed Res Int.

[34] Jain M, Bang A, Meshram P (2021). Institution of an antibiotic stewardship programme for rationalising antibiotic usage: a quality improvement project in the NICU of a public teaching hospital in rural central India. BMJ Open Qual.

[35] Jinka DR, Gandra S, Alvarez-Uria G, Torre N, Tadepalli D, Nayakanti RR (2017). Impact of antibiotic policy on antibiotic consumption in a neonatal intensive care unit in India. Indian Pediatr.

[36] Konda KC, Singh H, Madireddy A, Poodari MMR (2021). Quality improvement initiative approach to decrease the unindicated usage of antibiotics in a neonatal intensive care unit of a tertiary care teaching hospital in Hyderabad, India. BMJ Open Qual.

[37] Landre-Peigne C, Ka AS, Peigne V, Bougere J, Seye MN, Imbert P (2011). Efficacy of an infection control programme in reducing nosocomial bloodstream infections in a Senegalese neonatal unit. J Hosp Infect.

[38] Lu C, Liu Q, Yuan H, Wang L (2019). Implementation of the smart use of antibiotics program to reduce unnecessary antibiotic use in a neonatal ICU: a prospective interrupted time-series study in a developing country. Crit Care Med.

[39] Maalouf FI, Saad T, Zakhour R, Yunis K (2023). Successful establishment and five-year sustainability of a neonatal-specific antimicrobial stewardship program in a low middle-income country. Front Pharmacol.

[40] Olita'a D, Barnabas R, Vali Boma G, Pameh W, Vince J, Duke T (2019). Simplified management protocol for term neonates after prolonged rupture of membranes in a setting with high rates of neonatal sepsis and mortality: a quality improvement study. Arch Dis Child.

[41] Ren Z, Yang S, Han J (2023). Reduction of antibiotic use and multi-drug resistance bacteria infection in neonates after improvement of antibiotics use strategy in a level 4 neonatal intensive care unit in southern China. Eur J Clin Microbiol Infect Dis.

[42] Sowjanya SVNS, Venugopalan L (2018). Restriction of antimicrobial usage in a tertiary care neonatal unit in south India: a before after trial. Int J Nematol.

[43] Liu XL, Yang J, Chen XH, Hua ZY (2016). Effects of antibiotic stewardship on neonatal bloodstream infections. Zhongguo Dang Dai Er Ke Za Zhi.

[44] Yin L, He L, Miao J (2020). Actively surveillance and appropriate patients placements' contact isolation dramatically decreased Carbapenem-Resistant Enterobacteriaceae infection and colonization in pediatric patients in China. J Hosp Infect.

[45] Zain J, Asim M, Firdos K, Laique T (2021). Different management strategies for term newborns delivered with premature rupture of membranes. Pak J Med Health Sci.

[46] Akintan P, Oshun P, Osuagwu C (2024). Point prevalence surveys of antibiotic prescribing in children at a tertiary hospital in a resource constraint, low-income sub-Saharan African country—the impact of an antimicrobial stewardship program. BMC Pediatr.

[47] Chimhini G, Chimhuya S, Madzudzo L (2020). Auditing use of antibiotics in Zimbabwean neonates. Infect Prev Pract.

[48] Chu M, Lin J, Wang M (2023). Restrictive use of empirical antibiotics is associated with improved short term outcomes in very low birth weight infants: a single center, retrospective cohort study from China. Antibiotics (Basel).

[49] Kommalur A, Baddadka V, Devadas S (2021). Decreasing antibiotic over-use by implementation of an antibiotic stewardship programme in preterm neonates in resource limited settings—a quality improvement initiative. Paediatr Int Child Health.

[50] Sathyan S, Pournami F, Prithvi AK, Nandakumar A, Prabhakar J, Jain N (2022). Optimizing antibiotic use in culture-negative healthcare-associated infection with a ‘stop’ policy: a descriptive analytical study. J Trop Pediatr.

[51] Wang B, Li G, Jin F (2020). Effect of weekly antibiotic round on antibiotic use in the neonatal intensive care unit as antibiotic stewardship strategy. Front Pediatr.

[52] Dramowski A, Prusakov P, Goff DA (2024). Prospective antimicrobial stewardship interventions by multidisciplinary teams to reduce neonatal antibiotic use in South Africa: the Neonatal Antimicrobial Stewardship (NeoAMS) study. Int J Infect Dis.

[53] Farias-Filho FA, Souza PV, Nascimento SNdR, Rodrigues RdC, Nascimento MMGd, Carvalho VdF (2024). Multiple-step antimicrobial stewardship approach in a neonatal intensive care unit: a quasi-experimental study. Rev Epidemiol Controle Infec.

[54] Nygren D, Andersson F, Barrow E (2025). Bloodstream infections and antimicrobial resistance in The Gambia: continuous surveillance from a tertiary care center. Am J Trop Med Hyg.

[55] Sewornu R, Boakye-Yiadom E, Ativi E (2025). Improved utilisation and quality of blood culture services following operational research in a tertiary hospital in Ghana. Trop Med Infect Dis.

[56] Garpvall K, Duong V, Linnros S (2021). Admission screening and cohort care decrease carbapenem resistant enterobacteriaceae in Vietnamese pediatric ICU's. Antimicrob Resist Infect Control.

[57] Pokharel S, Raut S, Adhikari B (2019). Tackling antimicrobial resistance in low-income and middle-income countries. BMJ Glob Health.

[58] Auta A, Hadi MA, Oga E (2019). Global access to antibiotics without prescription in community pharmacies: a systematic review and meta-analysis. J Infect.

[59] Do NTT, Vu HTL, Nguyen CTK (2021). Community-based antibiotic access and use in six low-income and middle-income countries: a mixed-method approach. Lancet Glob Health.

[60] Patel J, Harant A, Fernandes G (2023). Measuring the global response to antimicrobial resistance, 2020–21: a systematic governance analysis of 114 countries. Lancet Infect Dis.

[61] Rajar P, Saugstad OD, Berild D (2020). Antibiotic stewardship in premature infants: a systematic review. Neonatology.

[62] Mascarenhas D, Ho MSP, Ting J, Shah PS (2024). Antimicrobial stewardship programs in neonates: a meta-analysis. Pediatrics.

[63] Donà D, Barbieri E, Daverio M (2020). Implementation and impact of pediatric antimicrobial stewardship programs: a systematic scoping review. Antimicrob Resist Infect Control.

[64] Harun MGD, Sumon SA, Hasan I, Akther FM, Islam MS, Anwar MMU (2024). Barriers, facilitators, perceptions and impact of interventions in implementing antimicrobial stewardship programs in hospitals of low-middle and middle countries: a scoping review. Antimicrob Resist Infect Control.

[65] Wu S, Tannous E, Haldane V, Ellen ME, Wei X (2022). Barriers and facilitators of implementing interventions to improve appropriate antibiotic use in low- and middle-income countries: a systematic review based on the Consolidated Framework for Implementation Research. Implement Sci.

[66] Otaigbe II, Elikwu CJ (2023). Drivers of inappropriate antibiotic use in low- and middle-income countries. JAC Antimicrob Resist.

[67] Mendelson M, Lewnard JA, Sharland M (2024). Ensuring progress on sustainable access to effective antibiotics at the 2024 UN General Assembly: a target-based approach. Lancet.

[68] OECD (2023).

[69] Lewnard JA, Charani E, Gleason A (2024). Burden of bacterial antimicrobial resistance in low-income and middle-income countries avertible by existing interventions: an evidence review and modelling analysis. Lancet.

[70] Frieden TR (2010). A framework for public health action: the health impact pyramid. Am J Public Health.

[71] United Nations General Assembly Political declaration of the high-level meeting on antimicrobial resistance. https://www.un.org/pga/wp-content/uploads/sites/108/2024/09/FINAL-Text-AMR-to-PGA.pdf.

[72] Goldmann D, Rajan S, Udayakumar K (2024). Preventing and controlling global antimicrobial resistance - implementing a whole-system approach. N Engl J Med.

[73] Gleeson B, Ferreyra C, Palamountain K (2024). A call to bridge the diagnostic gap: diagnostic solutions for neonatal sepsis in low- and middle-income countries. BMJ Glob Health.

[74] Okeke IN, Feasey N, Parkhill J (2020). Leapfrogging laboratories: the promise and pitfalls of high-tech solutions for antimicrobial resistance surveillance in low-income settings. BMJ Glob Health.

[75] Orekan J, Barbé B, Oeng S (2021). Culture media for clinical bacteriology in low- and middle-income countries: challenges, best practices for preparation and recommendations for improved access. Clin Microbiol Infect.

