# Supplementary material for: Strategies to reduce antimicrobial resistance in newborns in low-income and middle-income countries: a systematic review and meta-analysis
Source: Lancet Glob Health. 2026 Mar 16;14(4):e524–38. doi: 10.1016/S2214-109X(25)00533-9 (PMC13000118; doi:10.1016/S2214-109X(25)00533-9)
Supplement: Supplementary appendix [file mmc1.pdf]

THE LANCET
Global Health

Supplementary appendix

This appendix formed part of the original submission and has been peer reviewed. We post it as supplied by the authors.

Supplement to: Lee Him R, Sihota D, Harrison L, et al. Strategies to reduce antimicrobial resistance in newborns in low-income and middle-income countries: a systematic review and meta-analysis. *Lancet Glob Health* 2026; **14:** e524–38.

Strategies to reduce AMR in newborns in LMICs

Supplementary Material

Table of Contents

Appendix A: PRISMA checklists 2

Appendix B: Search strategies 8

Appendix C: Eligibility criteria 20

Appendix D: Eligibility criteria, outcomes defined 22

Appendix E: List of excluded studies with reasons 27

Appendix F: WHO EMLc AWaRe classification 31

Appendix G: Characteristics of included studies 33

Appendix H: Geographical distribution of studies implementing strategies to reduce AMR 78

Appendix I: Key characteristics of included studies tabulated 79

Appendix J: Risk of bias assessment 81

Appendix K: GRADE certainty of evidence for neonatal mortality outcomes 85

Appendix L: Forest plots 88

Regulation 88
Optimization 90
Regulation and Optimization 92
Regulation, Education, and Optimization 93

Appendix M: Forest plots for risk differences (RDs) 104

Regulation 104
Optimization 105
Regulation and Optimization 108
Regulation, Education, and Optimization 109

Appendix N: Effect direction plot of secondary outcomes 120

References 122

Appendix A: PRISMA checklists

Table 1: **PRISMA 2020 checklist for reporting systematic reviews**

Section and Topic	Item #	Checklist item	Location where item is reported
**TITLE**			
Title	1	Identify the report as a systematic review.	Pg. 1
**ABSTRACT**			
Abstract	2	See the PRISMA 2020 for Abstracts checklist.	See Table 2: PRISMA 2020 checklist for abstracts
**INTRODUCTION**			
Rationale	3	Describe the rationale for the review in the context of existing knowledge.	Pg. 5
Objectives	4	Provide an explicit statement of the objective(s) or question(s) the review addresses.	Pg. 5
**METHODS**			
Eligibility criteria	5	Specify the inclusion and exclusion criteria for the review and how studies were grouped for the syntheses.	Pg. 5-6 (inclusion/exclusion criteria; also, Appendix C & D for details), Pg. 6-7 (grouping for synthesis)
Information sources	6	Specify all databases, registers, websites, organisations, reference lists and other sources searched or consulted to identify studies. Specify the date when each source was last searched or consulted.	Pg. 5
Search strategy	7	Present the full search strategies for all databases, registers and websites, including any filters and limits used.	Appendix B for full search strategies
Selection process	8	Specify the methods used to decide whether a study met the inclusion criteria of the review, including how many reviewers screened each record and each report retrieved, whether they worked independently, and if applicable, details of automation tools used in the process.	Pg. 5-6

Section and Topic	Item #	Checklist item	Location where item is reported
Data collection process	9	Specify the methods used to collect data from reports, including how many reviewers collected data from each report, whether they worked independently, any processes for obtaining or confirming data from study investigators, and if applicable, details of automation tools used in the process.	Pg. 6
Data items	10a	List and define all outcomes for which data were sought. Specify whether all results that were compatible with each outcome domain in each study were sought (e.g. for all measures, time points, analyses), and if not, the methods used to decide which results to collect.	Pg. 5-6 (Appendix D for full definitions)
	10b	List and define all other variables for which data were sought (e.g. participant and intervention characteristics, funding sources). Describe any assumptions made about any missing or unclear information.	Pg. 6
Study risk of bias assessment	11	Specify the methods used to assess risk of bias in the included studies, including details of the tool(s) used, how many reviewers assessed each study and whether they worked independently, and if applicable, details of automation tools used in the process.	Pg. 6
Effect measures	12	Specify for each outcome the effect measure(s) (e.g. risk ratio, mean difference) used in the synthesis or presentation of results.	Pg. 7
Synthesis methods	13a	Describe the processes used to decide which studies were eligible for each synthesis (e.g. tabulating the study intervention characteristics and comparing against the planned groups for each synthesis (item #5)).	Pg. 5-7
	13b	Describe any methods required to prepare the data for presentation or synthesis, such as handling of missing summary statistics, or data conversions.	Pg. 6
	13c	Describe any methods used to tabulate or visually display results of individual studies and syntheses.	Pg. 7 (for meta-analysis methods); Pg. 8 (for narrative synthesis methods)
	13d	Describe any methods used to synthesize results and provide a rationale for the choice(s). If meta-analysis was performed, describe the model(s), method(s) to identify the presence and extent of statistical heterogeneity, and software package(s) used.	Pg. 7 (meta-analysis); Pg. 8 (narrative synthesis)
	13e	Describe any methods used to explore possible causes of heterogeneity among study results	Pg. 7 (for subgroup

Section and Topic	Item #	Checklist item	Location where item is reported
		(e.g. subgroup analysis, meta-regression).	analysis methods)
	13f	Describe any sensitivity analyses conducted to assess robustness of the synthesized results.	Pg. 7
Reporting bias assessment	14	Describe any methods used to assess risk of bias due to missing results in a synthesis (arising from reporting biases).	Pg. 7
Certainty assessment	15	Describe any methods used to assess certainty (or confidence) in the body of evidence for an outcome.	Pg. 7 (GRADE COE for mortality outcomes)
**RESULTS**			
Study selection	16a	Describe the results of the search and selection process, from the number of records identified in the search to the number of studies included in the review, ideally using a flow diagram.	Pg. 8 & Figure 2 (for PRISMA flow diagram)
	16b	Cite studies that might appear to meet the inclusion criteria, but which were excluded, and explain why they were excluded.	Pg. 6; Appendix E
Study characteristics	17	Cite each included study and present its characteristics.	Pg. 8; Appendix G-I for characteristics
Risk of bias in studies	18	Present assessments of risk of bias for each included study.	Pg. 9; Appendix J
Results of individual studies	19	For all outcomes, present, for each study: (a) summary statistics for each group (where appropriate) and (b) an effect estimate and its precision (e.g. confidence/credible interval), ideally using structured tables or plots.	Pg. 12-13; Figure 4 & Appendix N (ED plots)
Results of syntheses	20a	For each synthesis, briefly summarise the characteristics and risk of bias among contributing studies.	Pg. 9-12
	20b	Present results of all statistical syntheses conducted. If meta-analysis was done, present for each the summary estimate and its precision (e.g. confidence/credible interval) and measures of statistical heterogeneity. If comparing groups, describe the direction of the effect.	Pg. 9-12; Appendix L & M for forest plots
	20c	Present results of all investigations of possible causes of heterogeneity among study results.	Pg. 9-12; Appendix L & M for forest plots

Section and Topic	Item #	Checklist item	Location where item is reported
	20d	Present results of all sensitivity analyses conducted to assess the robustness of the synthesized results.	Pg. 12; Appendix L (see neonatal mortality outcome for regulation, education, and optimization)
Reporting biases	21	Present assessments of risk of bias due to missing results (arising from reporting biases) for each synthesis assessed.	N/A due to insufficient number of studies in meta-analysis
Certainty of evidence	22	Present assessments of certainty (or confidence) in the body of evidence for each outcome assessed.	Pg. 9; Appendix K
**DISCUSSION**			
Discussion	23a	Provide a general interpretation of the results in the context of other evidence.	Pg. 13-15
	23b	Discuss any limitations of the evidence included in the review.	Pg. 14
	23c	Discuss any limitations of the review processes used.	Pg. 14
	23d	Discuss implications of the results for practice, policy, and future research.	Pg. 16-17
**OTHER INFORMATION**			
Registration and protocol	24a	Provide registration information for the review, including register name and registration number, or state that the review was not registered.	Pg. 3
	24b	Indicate where the review protocol can be accessed, or state that a protocol was not prepared.	Pg. 3
	24c	Describe and explain any amendments to information provided at registration or in the protocol.	N/A
Support	25	Describe sources of financial or non-financial support for the review, and the role of the funders or sponsors in the review.	Pg. 3, 18
Competing interests	26	Declare any competing interests of review authors.	Pg. 18

Section and Topic	Item #	Checklist item	Location where item is reported
Availability of data, code and other materials	27	Report which of the following are publicly available and where they can be found: template data collection forms; data extracted from included studies; data used for all analyses; analytic code; any other materials used in the review.	Pg. 18

From: Page MJ, McKenzie JE, Bossuyt PM, Boutron I, Hoffmann TC, Mulrow CD, et al. The PRISMA 2020 statement: an updated guideline for reporting systematic reviews. BMJ 2021;372:n71. doi: 10.1136/bmj.n71. This work is licensed under CC BY 4.0. To view a copy of this license, visit https://creativecommons.org/licenses/by/4.0/

Table 2: **PRISMA 2020 checklist for abstracts**

Section and Topic	Item #	Checklist item	Reported (Yes/No)
**TITLE**			
Title	1	Identify the report as a systematic review.	Yes
**BACKGROUND**			
Objectives	2	Provide an explicit statement of the main objective(s) or question(s) the review addresses.	Yes
**METHODS**			
Eligibility criteria	3	Specify the inclusion and exclusion criteria for the review.	Yes
Information sources	4	Specify the information sources (e.g. databases, registers) used to identify studies and the date when each was last searched.	Yes
Risk of bias	5	Specify the methods used to assess risk of bias in the included studies.	Yes
Synthesis of results	6	Specify the methods used to present and synthesise results.	Yes
**RESULTS**			
Included studies	7	Give the total number of included studies and participants and summarise relevant characteristics of studies.	Yes
Synthesis of results	8	Present results for main outcomes, preferably indicating the number of included studies and participants for each. If meta-analysis was done, report the summary estimate and confidence/credible interval. If comparing groups, indicate the direction of the effect (i.e. which group is favoured).	Yes
**DISCUSSION**			

Section and Topic	Item #	Checklist item	Reported (Yes/No)
Limitations of evidence	9	Provide a brief summary of the limitations of the evidence included in the review (e.g. study risk of bias, inconsistency and imprecision).	Yes
Interpretation	10	Provide a general interpretation of the results and important implications.	Yes
**OTHER**			
Funding	11	Specify the primary source of funding for the review.	Yes
Registration	12	Provide the register name and registration number.	Yes

From: Page MJ, McKenzie JE, Bossuyt PM, Boutron I, Hoffmann TC, Mulrow CD, et al. The PRISMA 2020 statement: an updated guideline for reporting systematic reviews. BMJ 2021;372:n71. doi: 10.1136/bmj.n71. This work is licensed under CC BY 4.0. Appendix B: Search strategies

Appendix B: Search strategies

Table 3: **Search strategy for Ovid MEDLINE, with date of final search on November 20, 2025**

**1**	(newborn* or neonat* or infant* or baby or babies or birth* or deliver* or labo?r).ti.
**2**	exp Infant, Newborn/ or Neonatology/ or Perinatology/ or Intensive Care, Neonatal/
**3**	1 or 2
**4**	(((antibiotic* or antimicrobial*) and (resistan* or stewardship)) or AMR).ti.
**5**	Drug Resistance, Microbial/ or exp Drug Resistance, Bacterial/ or Drug Resistance, Multiple, Bacterial/ or Antimicrobial Stewardship/
**6**	4 or 5
**7**	("bacterial infection*" or "nosocomial infection*" or HAI* or "healthcare-associated infection*" or "health care-associated infection*" or sepsis or coloni#* or hygien* or cleaning or disinfect* or sterili$* or saniti$* or sanitary or susceptibility or culture* or antibiogram* or surveillance or monitoring or management or administration or utilization or usage or overuse or over-use or prescribing or prescription* or prophylaxis or therapy or treatment* or regimen*).ti.
**8**	exp Bacterial Infections/ or exp Cross Infection/ or exp Infection Control/ or Intensive Care Units, Neonatal/ or Antibiotic Prophylaxis/ or exp Microbial Sensitivity Tests/
**9**	7 or 8
**10**	(afghanistan or albania or algeria or "american samoa" or angola or "antigua and barbuda" or antigua or barbuda or argentina or armenia or armenian or aruba or azerbaijan or bahrain or bangladesh or barbados or "republic of belarus" or belarus or byelarus or belorussia or byelorussian or belize or "british honduras" or benin or dahomey or bhutan or bolivia or "bosnia and herzegovina" or bosnia or herzegovina or botswana or bechuanaland or brazil or brasil or bulgaria or "burkina faso" or "burkina fasso" or "upper volta" or burundi or urundi or "cabo verde" or "cape verde" or cambodia or kampuchea or "khmer republic" or cameroon or cameron or cameroun or "central african republic" or "ubangi shari" or chad or chile or china or colombia or comoros or "comoro islands" or "iles comores" or mayotte or "democratic republic of the congo" or "democratic republic congo" or congo or zaire or "costa rica" or "cote d’ivoire" or "cote d’ ivoire" or "cote divoire" or "cote d ivoire" or "ivory coast" or croatia or cuba or cyprus or "czech republic" or czechoslovakia or djibouti or "french somaliland" or dominica or "dominican republic" or ecuador or egypt or "united arab republic" or "el salvador" or "equatorial guinea" or "spanish guinea" or eritrea or estonia or eswatini or swaziland or ethiopia or fiji or gabon or "gabonese republic" or gambia or "georgia (republic)" or georgian or ghana or "gold coast" or gibraltar or greece or grenada or guam or guatemala or guinea or "guinea bissau" or guyana or "british guiana" or haiti or hispaniola or honduras or hungary or india or indonesia or timor or iran or iraq or "isle of man" or jamaica or jordan or kazakhstan or kazakh or kenya or "democratic people’s republic of

	korea" or "republic of korea" or "north korea" or "south korea" or korea or kosovo or kyrgyzstan or kirghizia or kirgizstan or "kyrgyz republic" or kirghiz or laos or "lao pdr" or "lao people's democratic republic" or latvia or lebanon or "lebanese republic" or lesotho or basutoland or liberia or libya or "libyan arab jamahiriya" or lithuania or macau or macao or "republic of north macedonia" or macedonia or madagascar or "malagasy republic" or malawi or nyasaland or malaysia or "malay federation" or "malaya federation" or maldives or "indian ocean islands" or "indian ocean" or mali or malta or micronesia or "federated states of micronesia" or kiribati or "marshall islands" or nauru or "northern mariana islands" or palau or tuvalu or mauritania or mauritius or mexico or moldova or moldovian or mongolia or montenegro or morocco or ifni or mozambique or "portuguese east africa" or myanmar or burma or namibia or nepal or "netherlands antilles" or nicaragua or niger or nigeria or oman or muscat or pakistan or panama or "papua new guinea" or "new guinea" or paraguay or peru or philippines or philipines or phillipines or phillippines or poland or "polish people's republic" or portugal or "portuguese republic" or "puerto rico" or romania or russia or "russian federation" or rwanda or ruanda or samoa or "pacific islands" or polynesia or "samoan islands" or "navigator island" or "navigator islands" or "sao tome and principe" or "saudi arabia" or senegal or serbia or seychelles or "sierra leone" or slovakia or "slovak republic" or slovenia or melanesia or "solomon island" or "solomon islands" or "norfolk island" or "norfolk islands" or somalia or "south africa" or "south sudan" or "sri lanka" or ceylon or "saint kitts and nevis" or "st. kitts and nevis" or "saint lucia" or "st. lucia" or "saint vincent and the grenadines" or "saint vincent" or "st. vincent" or grenadines or sudan or suriname or surinam or "dutch guiana" or "netherlands guiana" or syria or "syrian arab republic" or tajikistan or tadjikistan or tadzhikistan or tadzhik or tanzania or tanganyika or thailand or siam or "timor leste" or "east timor" or togo or "togolese republic" or tonga or "trinidad and tobago" or trinidad or tobago or tunisia or turkey or turkmenistan or turkmen or uganda or ukraine or uruguay or uzbekistan or uzbek or vanuatu or "new hebrides" or venezuela or vietnam or "viet nam" or "middle east" or "west bank" or gaza or palestine or yemen or yugoslavia or zambia or zimbabwe or "northern rhodesia" or "global south" or "africa south of the sahara" or "sub-saharan africa" or "subsaharan africa" or "africa, central" or "central africa" or "africa, northern" or "north africa" or "northern africa" or magreb or maghrib or sahara or "africa, southern" or "southern africa" or "africa, eastern" or "east africa" or "eastern africa" or "africa, western" or "west africa" or "western africa" or "west indies" or "indian ocean islands" or caribbean or "central america" or "latin america" or "south and central america" or "south america" or "asia, central" or "central asia" or "asia, northern" or "north asia" or "northern asia" or "asia, southeastern" or "southeastern asia" or "south eastern asia" or "southeast asia" or "south east asia" or "asia, western" or "western asia" or "europe, eastern" or "east europe" or "eastern europe" or "developing country" or "developing countries" or "developing nation?" or "developing population?" or "developing world" or "less developed countr*" or "less developed nation?" or "less developed population?" or "less developed world" or "lesser developed countr*" or "lesser developed nation?" or "lesser developed population?" or "lesser developed world" or "under developed countr*" or "under developed nation?" or "under developed population?" or "under

	developed world" or "underdeveloped countr*" or "underdeveloped nation?" or "underdeveloped population?" or "underdeveloped world" or "middle income countr*" or "middle income nation?" or "middle income population?" or "low income countr*" or "low income nation?" or "low income population?" or "lower income countr*" or "lower income nation?" or "lower income population?" or "underserved countr*" or "underserved nation?" or "underserved population?" or "underserved world" or "under served countr*" or "under served nation?" or "under served population?" or "under served world" or "deprived countr*" or "deprived nation?" or "deprived population?" or "deprived world" or "poor countr*" or "poor nation?" or "poor population?" or "poor world" or "poorer countr*" or "poorer nation?" or "poorer population?" or "poorer world" or "developing econom*" or "less developed econom*" or "lesser developed econom*" or "under developed econom*" or "underdeveloped econom*" or "middle income econom*" or "low income econom*" or "lower income econom*" or "low gdp" or "low gnp" or "low gross domestic" or "low gross national" or "lower gdp" or "lower gnp" or "lower gross domestic" or "lower gross national" or lmic or lmics or "third world" or "lami countr*" or "transitional countr*" or "emerging economies" or "emerging nation?" or "low-resource setting*" or "low-resource nation*" or "low-resource countr*" or "resource-limiting setting*" or "resource-limiting nation*" or "resource-limiting countr*").ti,sh.
**11**	3 and 6 and 9 and 10

Table 4: **Search strategy for Ovid EMBASE, with date of final search on November 20, 2025**

**1**	(newborn* or neonat* or infant* or baby or babies or birth* or deliver* or labo?r).ti.
**2**	Newborn/ or exp Neonatology/ or Newborn Intensive Care/
**3**	1 or 2
**4**	(((antibiotic* or antimicrobial*) and (resistan* or stewardship)) or AMR).ti.
**5**	exp Antibiotic Resistance/ or Multidrug Resistance/ or Antimicrobial Stewardship/
**6**	4 or 5
**7**	("bacterial infection*" or "nosocomial infection*" or HAI* or "healthcare-associated infection*" or "health care-associated infection*" or sepsis or coloni#* or hygien* or cleaning or disinfect* or sterili$* or saniti$* or sanitary or susceptibility or culture* or antibiogram* or surveillance or monitoring or management or administration or utilization or usage or overuse or over-use or prescribing or prescription* or prophylaxis or therapy or treatment* or regimen*).ti.
**8**	exp Bacterial Infection/ or Cross Infection/ or exp Infection Control/ or Neonatal Intensive Care Unit/ or Antibiotic Prophylaxis/ or Antibiotic Therapy/ or Microbial Sensitivity Test/
**9**	7 or 8
**10**	(afghanistan or albania or algeria or american samoa or angola or "antigua and barbuda" or antigua or barbuda or argentina or armenia or armenian or aruba or azerbaijan or bahrain or bangladesh or barbados or republic of belarus or belarus or

	byelarus or belorussia or byelorussian or belize or british honduras or benin or dahomey or bhutan or bolivia or "bosnia and herzegovina" or bosnia or herzegovina or botswana or bechuanaland or brazil or brasil or bulgaria or burkina faso or burkina fasso or upper volta or burundi or urundi or cabo verde or cape verde or cambodia or kampuchea or khmer republic or cameroon or cameron or cameroun or central african republic or ubangi shari or chad or chile or china or colombia or comoros or comoro islands or iles comores or mayotte or democratic republic of the congo or democratic republic congo or congo or zaire or costa rica or "cote d'ivoire" or "cote d' ivoire" or cote divoire or cote d ivoire or ivory coast or croatia or cuba or cyprus or czech republic or czechoslovakia or djibouti or french somaliland or dominica or dominican republic or ecuador or egypt or united arab republic or el salvador or equatorial guinea or spanish guinea or eritrea or estonia or eswatini or swaziland or ethiopia or fiji or gabon or gabonese republic or gambia or "georgia (republic)" or georgian or ghana or gold coast or gibraltar or greece or grenada or guam or guatemala or guinea or guinea bissau or guyana or british guiana or haiti or hispaniola or honduras or hungary or india or indonesia or timor or iran or iraq or isle of man or jamaica or jordan or kazakhstan or kazakh or kenya or "democratic people's republic of korea" or republic of korea or north korea or south korea or korea or kosovo or kyrgyzstan or kirghizia or kirgizstan or kyrgyz republic or kirghiz or laos or lao pdr or "lao people's democratic republic" or latvia or lebanon or lebanese republic or lesotho or basutoland or liberia or libya or libyan arab jamahiriya or lithuania or macau or macao or republic of north macedonia or macedonia or madagascar or malagasy republic or malawi or nyasaland or malaysia or malay federation or malaya federation or maldives or indian ocean islands or indian ocean or mali or malta or micronesia or federated states of micronesia or kiribati or marshall islands or nauru or northern mariana islands or palau or tuvalu or mauritania or mauritius or mexico or moldova or moldovian or mongolia or montenegro or "montenegro (republic)" or morocco or ifni or mozambique or portuguese east africa or myanmar or burma or namibia or nepal or netherlands antilles or nicaragua or niger or nigeria or oman or muscat or pakistan or panama or papua new guinea or new guinea or paraguay or peru or philippines or philipines or phillipines or phillippines or poland or "polish people's republic" or portugal or portuguese republic or puerto rico or romania or russia or russian federation or rwanda or ruanda or samoa or pacific islands or polynesia or samoan islands or navigator island or navigator islands or "sao tome and principe" or saudi arabia or senegal or serbia or seychelles or sierra leone or slovakia or slovak republic or slovenia or melanesia or solomon island or solomon islands or norfolk island or norfolk islands or somalia or south africa or south sudan or sri lanka or ceylon or "saint kitts and nevis" or "st. kitts and nevis" or saint lucia or "st. lucia" or "saint vincent and the grenadines" or saint vincent or "st. vincent" or grenadines or sudan or suriname or surinam or dutch guiana or netherlands guiana or syria or syrian arab republic or tajikistan or tadjikistan or tadzhikistan or tadzhik or tanzania or tanganyika or thailand or siam or timor leste or east timor or togo or togolese republic or tonga or "trinidad and tobago" or trinidad or tobago or tunisia or "turkey (republic)" or turkey or turkmenistan or turkmen or uganda or ukraine or uruguay or uzbekistan or uzbek or vanuatu or new hebrides or venezuela or vietnam or viet nam or middle east or west bank or gaza or

	palestine or yemen or yugoslavia or zambia or zimbabwe or northern rhodesia or global south or africa south of the sahara or "sub saharan africa" or subsaharan africa or africa, central or central africa or africa, northern or north africa or northern africa or magreb or maghrib or sahara or africa, southern or southern africa or africa, eastern or east africa or eastern africa or africa, western or west africa or western africa or west indies or indian ocean islands or caribbean region or caribbean islands or caribbean or central america or latin america or "south and central america" or south america or asia, central or central asia or asia, northern or north asia or northern asia or asia, southeastern or southeastern asia or south eastern asia or southeast asia or south east asia or asia, western or western asia or europe, eastern or east europe or eastern europe or developing country or developing countries or developing nation? or developing population? or developing world or less developed countr* or less developed nation? or less developed population? or less developed world or lesser developed countr* or lesser developed nation? or lesser developed population? or lesser developed world or under developed countr* or under developed nation? or under developed population? or under developed world or underdeveloped countr* or underdeveloped nation? or underdeveloped population? or underdeveloped world or middle income countr* or middle income nation? or middle income population? or low income countr* or low income nation? or low income population? or lower income countr* or lower income nation? or lower income population? or underserved countr* or underserved nation? or underserved population? or underserved world or under served countr* or under served nation? or under served population? or under served world or deprived countr* or deprived nation? or deprived population? or deprived world or poor countr* or poor nation? or poor population? or poor world or poorer countr* or poorer nation? or poorer population? or poorer world or developing econom* or less developed econom* or lesser developed econom* or under developed econom* or underdeveloped econom* or middle income econom* or low income econom* or lower income econom* or low gdp or low gnp or low gross domestic or low gross national or lower gdp or lower gnp or lower gross domestic or lower gross national or lmic or lmics or third world or lami countr* or transitional countr* or emerging economies or emerging nation? or "low-resource setting*" or "low-resource nation*" or "low-resource countr*" or "resource-limiting setting*" or "resource-limiting nation*" or "resource-limiting countr*").ti,sh.
**11**	3 and 6 and 9 and 10

Table 5: **Search strategy for EBSCO CINAHL Plus, with date of final search on November 20, 2025**

**S1**	TI (newborn* or neonat* or infant* or baby or babies or birth* or deliver* or labo?r)
**S2**	(MH “Infant, Newborn+”) or (MH “Neonatology”) or (MH “Perinatology”) or (MH “Intensive Care, Neonatal”)
**S3**	S1 or S2
**S4**	TI (((antibiotic* or antimicrobial*) and (resistan* or stewardship)) or AMR)
**S5**	(MH “Drug Resistance, Microbial+”) or (MH “Antimicrobial Stewardship”)

**S6**	S4 or S5
**S7**	TI ("bacterial infection*" or "nosocomial infection*" or HAI* or "healthcare-associated infection*" or "health care-associated infection*" or sepsis or coloni#* or hygien* or cleaning or disinfect* or sterili$* or saniti$* or sanitary or susceptibility or culture* or antibiogram* or surveillance or monitoring or management or administration or utilization or usage or overuse or over-use or prescribing or prescription* or prophylaxis or therapy or treatment* or regimen*)
**S8**	(MH “Bacterial Infections+”) or (MH “Cross Infection+”) or (MH “Infection Control+”) or (MH “Intensive Care Units, Neonatal”) or (MH “Antibiotic Prophylaxis”) or (MH “Microbial Culture and Sensitivity Tests”)
**S9**	S7 or S8
**S10**	TI (afghanistan or albania or algeria or "american samoa" or angola or "antigua and barbuda" or antigua or barbuda or argentina or armenia or armenian or aruba or azerbaijan or bahrain or bangladesh or barbados or "republic of belarus" or belarus or byelarus or belorussia or byelorussian or belize or "british honduras" or benin or dahomey or bhutan or bolivia or "bosnia and herzegovina" or bosnia or herzegovina or botswana or bechuanaland or brazil or brasil or bulgaria or "burkina faso" or "burkina fasso" or "upper volta" or burundi or urundi or "cabo verde" or "cape verde" or cambodia or kampuchea or "khmer republic" or cameroon or cameron or cameroun or "central african republic" or "ubangi shari" or chad or chile or china or colombia or comoros or "comoro islands" or "iles comores" or mayotte or "democratic republic of the congo" or "democratic republic congo" or congo or zaire or "costa rica" or "cote d’ivoire" or "cote d’ ivoire" or "cote divoire" or "cote d ivoire" or "ivory coast" or croatia or cuba or cyprus or "czech republic" or czechoslovakia or djibouti or "french somaliland" or dominica or "dominican republic" or ecuador or egypt or "united arab republic" or "el salvador" or "equatorial guinea" or "spanish guinea" or eritrea or estonia or eswatini or swaziland or ethiopia or fiji or gabon or "gabonese republic" or gambia or "georgia (republic)" or georgian or ghana or "gold coast" or gibraltar or greece or grenada or guam or guatemala or guinea or "guinea bissau" or guyana or "british guiana" or haiti or hispaniola or honduras or hungary or india or indonesia or timor or iran or iraq or "isle of man" or jamaica or jordan or kazakhstan or kazakh or kenya or "democratic people’s republic of korea" or "republic of korea" or "north korea" or "south korea" or korea or kosovo or kyrgyzstan or kirghizia or kirgizstan or "kyrgyz republic" or kirghiz or laos or "lao pdr" or "lao people's democratic republic" or latvia or lebanon or "lebanese republic" or lesotho or basutoland or liberia or libya or "libyan arab jamahiriya" or lithuania or macau or macao or "republic of north macedonia" or macedonia or madagascar or "malagasy republic" or malawi or nyasaland or malaysia or "malay federation" or "malaya federation" or maldives or "indian ocean islands" or "indian ocean" or mali or malta or micronesia or "federated states of micronesia" or kiribati or "marshall islands" or nauru or "northern mariana islands" or palau or tuvalu or mauritania or mauritius or mexico or moldova or moldovian or mongolia or montenegro or morocco or ifni or mozambique or "portuguese east africa" or myanmar or burma or namibia or nepal or "netherlands antilles" or nicaragua or niger or nigeria or oman or muscat or pakistan or panama or "papua new guinea" or "new guinea" or paraguay or peru or philippines

	or philipines or phillipines or phillippines or poland or "polish people's republic" or portugal or "portuguese republic" or "puerto rico" or romania or russia or "russian federation" or rwanda or ruanda or samoa or "pacific islands" or polynesia or "samoan islands" or "navigator island" or "navigator islands" or "sao tome and principe" or "saudi arabia" or senegal or serbia or seychelles or "sierra leone" or slovakia or "slovak republic" or slovenia or melanesia or "solomon island" or "solomon islands" or "norfolk island" or "norfolk islands" or somalia or "south africa" or "south sudan" or "sri lanka" or ceylon or "saint kitts and nevis" or "st. kitts and nevis" or "saint lucia" or "st. lucia" or "saint vincent and the grenadines" or "saint vincent" or "st. vincent" or grenadines or sudan or suriname or surinam or "dutch guiana" or "netherlands guiana" or syria or "syrian arab republic" or tajikistan or tadjikistan or tadzhikistan or tadzhik or tanzania or tanganyika or thailand or siam or "timor leste" or "east timor" or togo or "togolese republic" or tonga or "trinidad and tobago" or trinidad or tobago or tunisia or turkey or turkmenistan or turkmen or uganda or ukraine or uruguay or uzbekistan or uzbek or vanuatu or "new hebrides" or venezuela or vietnam or "viet nam" or "middle east" or "west bank" or gaza or palestine or yemen or yugoslavia or zambia or zimbabwe or "northern rhodesia" or "global south" or "africa south of the sahara" or "sub-saharan africa" or "subsaharan africa" or "africa, central" or "central africa" or "africa, northern" or "north africa" or "northern africa" or magreb or maghrib or sahara or "africa, southern" or "southern africa" or "africa, eastern" or "east africa" or "eastern africa" or "africa, western" or "west africa" or "western africa" or "west indies" or "indian ocean islands" or caribbean or "central america" or "latin america" or "south and central america" or "south america" or "asia, central" or "central asia" or "asia, northern" or "north asia" or "northern asia" or "asia, southeastern" or "southeastern asia" or "south eastern asia" or "southeast asia" or "south east asia" or "asia, western" or "western asia" or "europe, eastern" or "east europe" or "eastern europe" or "developing country" or "developing countries" or "developing nation?" or "developing population?" or "developing world" or "less developed countr*" or "less developed nation?" or "less developed population?" or "less developed world" or "lesser developed countr*" or "lesser developed nation?" or "lesser developed population?" or "lesser developed world" or "under developed countr*" or "under developed nation?" or "under developed population?" or "under developed world" or "underdeveloped countr*" or "underdeveloped nation?" or "underdeveloped population?" or "underdeveloped world" or "middle income countr*" or "middle income nation?" or "middle income population?" or "low income countr*" or "low income nation?" or "low income population?" or "lower income countr*" or "lower income nation?" or "lower income population?" or "underserved countr*" or "underserved nation?" or "underserved population?" or "underserved world" or "under served countr*" or "under served nation?" or "under served population?" or "under served world" or "deprived countr*" or "deprived nation?" or "deprived population?" or "deprived world" or "poor countr*" or "poor nation?" or "poor population?" or "poor world" or "poorer countr*" or "poorer nation?" or "poorer population?" or "poorer world" or "developing econom*" or "less developed econom*" or "lesser developed econom*" or "under developed econom*" or "underdeveloped econom*" or "middle income econom*" or "low income econom*" or "lower income

	econom*" or "low gdp" or "low gnp" or "low gross domestic" or "low gross national" or "lower gdp" or "lower gnp" or "lower gross domestic" or "lower gross national" or lmic or lmics or "third world" or "lami countr*" or "transitional countr*" or "emerging economies" or "emerging nation?" or "low-resource setting*" or "low-resource nation*" or "low-resource countr*" or "resource-limiting setting*" or "resource-limiting nation*" or "resource-limiting countr*")
**S11**	MH (afghanistan or albania or algeria or "american samoa" or angola or "antigua and barbuda" or antigua or barbuda or argentina or armenia or armenian or aruba or azerbaijan or bahrain or bangladesh or barbados or "republic of belarus" or belarus or byelarus or belorussia or byelorussian or belize or "british honduras" or benin or dahomey or bhutan or bolivia or "bosnia and herzegovina" or bosnia or herzegovina or botswana or bechuanaland or brazil or brasil or bulgaria or "burkina faso" or "burkina fasso" or "upper volta" or burundi or urundi or "cabo verde" or "cape verde" or cambodia or kampuchea or "khmer republic" or cameroon or cameron or cameroun or "central african republic" or "ubangi shari" or chad or chile or china or colombia or comoros or "comoro islands" or "iles comores" or mayotte or "democratic republic of the congo" or "democratic republic congo" or congo or zaire or "costa rica" or "cote d'ivoire" or "cote d' ivoire" or "cote divoire" or "cote d ivoire" or "ivory coast" or croatia or cuba or cyprus or "czech republic" or czechoslovakia or djibouti or "french somaliland" or dominica or "dominican republic" or ecuador or egypt or "united arab republic" or "el salvador" or "equatorial guinea" or "spanish guinea" or eritrea or estonia or eswatini or swaziland or ethiopia or fiji or gabon or "gabonese republic" or gambia or "georgia (republic)" or georgian or ghana or "gold coast" or gibraltar or greece or grenada or guam or guatemala or guinea or "guinea bissau" or guyana or "british guiana" or haiti or hispaniola or honduras or hungary or india or indonesia or timor or iran or iraq or "isle of man" or jamaica or jordan or kazakhstan or kazakh or kenya or "democratic people's republic of korea" or "republic of korea" or "north korea" or "south korea" or korea or kosovo or kyrgyzstan or kirghizia or kirgizstan or "kyrgyz republic" or kirghiz or laos or "lao pdr" or "lao people's democratic republic" or latvia or lebanon or "lebanese republic" or lesotho or basutoland or liberia or libya or "libyan arab jamahiriya" or lithuania or macau or macao or "republic of north macedonia" or macedonia or madagascar or "malagasy republic" or malawi or nyasaland or malaysia or "malay federation" or "malaya federation" or maldives or "indian ocean islands" or "indian ocean" or mali or malta or micronesia or "federated states of micronesia" or kiribati or "marshall islands" or nauru or "northern mariana islands" or palau or tuvalu or mauritania or mauritius or mexico or moldova or moldovian or mongolia or montenegro or morocco or ifni or mozambique or "portuguese east africa" or myanmar or burma or namibia or nepal or "netherlands antilles" or nicaragua or niger or nigeria or oman or muscat or pakistan or panama or "papua new guinea" or "new guinea" or paraguay or peru or philippines or philipines or phillipines or phillippines or poland or "polish people's republic" or portugal or "portuguese republic" or "puerto rico" or romania or russia or "russian federation" or rwanda or ruanda or samoa or "pacific islands" or polynesia or "samoan islands" or "navigator island" or "navigator islands" or "sao tome and principe" or "saudi arabia" or senegal or serbia or seychelles or "sierra leone" or slovakia or "slovak republic" or slovenia or melanesia or "solomon island" or

	"solomon islands" or "norfolk island" or "norfolk islands" or somalia or "south africa" or "south sudan" or "sri lanka" or ceylon or "saint kitts and nevis" or "st. kitts and nevis" or "saint lucia" or "st. lucia" or "saint vincent and the grenadines" or "saint vincent" or "st. vincent" or grenadines or sudan or suriname or surinam or "dutch guiana" or "netherlands guiana" or syria or "syrian arab republic" or tajikistan or tadjikistan or tadzhikistan or tadzhik or tanzania or tanganyika or thailand or siam or "timor leste" or "east timor" or togo or "togolese republic" or tonga or "trinidad and tobago" or trinidad or tobago or tunisia or turkey or turkmenistan or turkmen or uganda or ukraine or uruguay or uzbekistan or uzbek or vanuatu or "new hebrides" or venezuela or vietnam or "viet nam" or "middle east" or "west bank" or gaza or palestine or yemen or yugoslavia or zambia or zimbabwe or "northern rhodesia" or "global south" or "africa south of the sahara" or "sub-saharan africa" or "subsaharan africa" or "africa, central" or "central africa" or "africa, northern" or "north africa" or "northern africa" or magreb or maghrib or sahara or "africa, southern" or "southern africa" or "africa, eastern" or "east africa" or "eastern africa" or "africa, western" or "west africa" or "western africa" or "west indies" or "indian ocean islands" or caribbean or "central america" or "latin america" or "south and central america" or "south america" or "asia, central" or "central asia" or "asia, northern" or "north asia" or "northern asia" or "asia, southeastern" or "southeastern asia" or "south eastern asia" or "southeast asia" or "south east asia" or "asia, western" or "western asia" or "europe, eastern" or "east europe" or "eastern europe" or "developing country" or "developing countries" or "developing nation?" or "developing population?" or "developing world" or "less developed countr*" or "less developed nation?" or "less developed population?" or "less developed world" or "lesser developed countr*" or "lesser developed nation?" or "lesser developed population?" or "lesser developed world" or "under developed countr*" or "under developed nation?" or "under developed population?" or "under developed world" or "underdeveloped countr*" or "underdeveloped nation?" or "underdeveloped population?" or "underdeveloped world" or "middle income countr*" or "middle income nation?" or "middle income population?" or "low income countr*" or "low income nation?" or "low income population?" or "lower income countr*" or "lower income nation?" or "lower income population?" or "underserved countr*" or "underserved nation?" or "underserved population?" or "underserved world" or "under served countr*" or "under served nation?" or "under served population?" or "under served world" or "deprived countr*" or "deprived nation?" or "deprived population?" or "deprived world" or "poor countr*" or "poor nation?" or "poor population?" or "poor world" or "poorer countr*" or "poorer nation?" or "poorer population?" or "poorer world" or "developing econom*" or "less developed econom*" or "lesser developed econom*" or "under developed econom*" or "underdeveloped econom*" or "middle income econom*" or "low income econom*" or "lower income econom*" or "low gdp" or "low gnp" or "low gross domestic" or "low gross national" or "lower gdp" or "lower gnp" or "lower gross domestic" or "lower gross national" or lmic or lmics or "third world" or "lami countr*" or "transitional countr*" or "emerging economies" or "emerging nation?" or "low-resource setting*" or "low-resource nation*" or "low-resource countr*" or "resource-limiting setting*" or "resource-limiting nation*" or "resource-limiting countr*")

**S12**	S10 or S11
**S13**	S3 and S6 and S9 and S12

Table 6: **Search strategy for the World Health Organization Global Index Medicus (GIM), with date of final search on November 20, 2025**

**Title-Only Search:**
(newborn* or neonat* or infant* or baby or babies or birth* or deliver* or labo?r) and (((antibiotic* or antimicrobial*) and (resistan* or stewardship)) or AMR)

Table 7: **Search strategy for Cochrane Library's Central Register of Controlled Trials (CENTRAL), with date of final search on November 20, 2025**

**#1**	(newborn* or neonat* or infant* or baby or babies or birth* or deliver* or labo?r):ti,ab,kw
**#2**	MeSH descriptor: [Infant, Newborn] explode all trees
**#3**	MeSH descriptor: [Neonatology] explode all trees
**#4**	MeSH descriptor: [Perinatology] explode all trees
**#5**	MeSH descriptor: [Intensive Care, Neonatal] explode all trees
**#6**	#1 or #2 or #3 or #4 or #5
**#7**	(((antibiotic* or antimicrobial*) and (resistan* or stewardship)) or AMR):ti,ab,kw
**#8**	MeSH descriptor: [Drug Resistance, Bacterial] explode all trees
**#9**	MeSH descriptor: [Antimicrobial Stewardship] explode all trees
**#10**	#7 or #8 or #9
**#11**	("bacterial infection*" or "nosocomial infection*" or HAI* or "healthcare-associated infection*" or "health care-associated infection*" or sepsis or coloni#* or hygien* or cleaning or disinfect* or sterili$* or saniti$* or sanitary or susceptibility or culture* or antibiogram* or surveillance or monitoring or management or administration or utilization or usage or overuse or over-use or prescribing or prescription* or prophylaxis or therapy or treatment* or regimen*):ti,ab,kw
**#12**	MeSH descriptor: [Bacterial Infections] explode all trees
**#13**	MeSH descriptor: [Cross Infection] explode all trees
**#14**	MeSH descriptor: [Infection Control] explode all trees
**#15**	MeSH descriptor: [Intensive Care Units, Neonatal] explode all trees
**#16**	MeSh descriptor: [Antibiotic Prophylaxis] explode all trees
**#17**	MeSH descriptor: [Microbial Sensitivity Tests] explode all trees

**#18**	#11 or #12 or #13 or #14 or #15 or #16 or #17
**#19**	(afghanistan or albania or algeria or "american samoa" or angola or "antigua and barbuda" or antigua or barbuda or argentina or armenia or armenian or aruba or azerbaijan or bahrain or bangladesh or barbados or "republic of belarus" or belarus or byelarus or belorussia or byelorussian or belize or "british honduras" or benin or dahomey or bhutan or bolivia or "bosnia and herzegovina" or bosnia or herzegovina or botswana or bechuanaland or brazil or brasil or bulgaria or "burkina faso" or "burkina fasso" or "upper volta" or burundi or urundi or "cabo verde" or "cape verde" or cambodia or kampuchea or "khmer republic" or cameroon or cameron or cameroun or "central african republic" or "ubangi shari" or chad or chile or china or colombia or comoros or "comoro islands" or "iles comores" or mayotte or "democratic republic of the congo" or "democratic republic congo" or congo or zaire or "costa rica" or "cote d'ivoire" or "cote d' ivoire" or "cote divoire" or "cote d ivoire" or "ivory coast" or croatia or cuba or cyprus or "czech republic" or czechoslovakia or djibouti or "french somaliland" or dominica or "dominican republic" or ecuador or egypt or "united arab republic" or "el salvador" or "equatorial guinea" or "spanish guinea" or eritrea or estonia or eswatini or swaziland or ethiopia or fiji or gabon or "gabonese republic" or gambia or "georgia (republic)" or georgian or ghana or "gold coast" or gibraltar or greece or grenada or guam or guatemala or guinea or "guinea bissau" or guyana or "british guiana" or haiti or hispaniola or honduras or hungary or india or indonesia or timor or iran or iraq or "isle of man" or jamaica or jordan or kazakhstan or kazakh or kenya or "democratic people's republic of korea" or "republic of korea" or "north korea" or "south korea" or korea or kosovo or kyrgyzstan or kirghizia or kirgizstan or "kyrgyz republic" or kirghiz or laos or "lao pdr" or "lao people's democratic republic" or latvia or lebanon or "lebanese republic" or lesotho or basutoland or liberia or libya or "libyan arab jamahiriya" or lithuania or macau or macao or "republic of north macedonia" or macedonia or madagascar or "malagasy republic" or malawi or nyasaland or malaysia or "malay federation" or "malaya federation" or maldives or "indian ocean islands" or "indian ocean" or mali or malta or micronesia or "federated states of micronesia" or kiribati or "marshall islands" or nauru or "northern mariana islands" or palau or tuvalu or mauritania or mauritius or mexico or moldova or moldovian or mongolia or montenegro or morocco or ifni or mozambique or "portuguese east africa" or myanmar or burma or namibia or nepal or "netherlands antilles" or nicaragua or niger or nigeria or oman or muscat or pakistan or panama or "papua new guinea" or "new guinea" or paraguay or peru or philippines or philipines or phillipines or phillippines or poland or "polish people's republic" or portugal or "portuguese republic" or "puerto rico" or romania or russia or "russian federation" or rwanda or ruanda or samoa or "pacific islands" or polynesia or "samoan islands" or "navigator island" or "navigator islands" or "sao tome and principe" or "saudi arabia" or senegal or serbia or seychelles or "sierra leone" or slovakia or "slovak republic" or slovenia or melanesia or "solomon island" or "solomon islands" or "norfolk island" or "norfolk islands" or somalia or "south africa" or "south sudan" or "sri lanka" or ceylon or "saint kitts and nevis" or "st. kitts and nevis" or "saint lucia" or "st. lucia" or "saint vincent and the grenadines" or "saint vincent" or "st. vincent" or grenadines or sudan or suriname or surinam or "dutch guiana" or "netherlands guiana" or

	syria or "syrian arab republic" or tajikistan or tadjikistan or tadzhikistan or tadzhik or tanzania or tanganyika or thailand or siam or "timor leste" or "east timor" or togo or "togolese republic" or tonga or "trinidad and tobago" or trinidad or tobago or tunisia or turkey or turkmenistan or turkmen or uganda or ukraine or uruguay or uzbekistan or uzbek or vanuatu or "new hebrides" or venezuela or vietnam or "viet nam" or "middle east" or "west bank" or gaza or palestine or yemen or yugoslavia or zambia or zimbabwe or "northern rhodesia" or "global south" or "africa south of the sahara" or "sub-saharan africa" or "subsaharan africa" or "africa, central" or "central africa" or "africa, northern" or "north africa" or "northern africa" or magreb or maghrib or sahara or "africa, southern" or "southern africa" or "africa, eastern" or "east africa" or "eastern africa" or "africa, western" or "west africa" or "western africa" or "west indies" or "indian ocean islands" or caribbean or "central america" or "latin america" or "south and central america" or "south america" or "asia, central" or "central asia" or "asia, northern" or "north asia" or "northern asia" or "asia, southeastern" or "southeastern asia" or "south eastern asia" or "southeast asia" or "south east asia" or "asia, western" or "western asia" or "europe, eastern" or "east europe" or "eastern europe" or "developing country" or "developing countries" or "developing nation?" or "developing population?" or "developing world" or "less developed countr*" or "less developed nation?" or "less developed population?" or "less developed world" or "lesser developed countr*" or "lesser developed nation?" or "lesser developed population?" or "lesser developed world" or "under developed countr*" or "under developed nation?" or "under developed population?" or "under developed world" or "underdeveloped countr*" or "underdeveloped nation?" or "underdeveloped population?" or "underdeveloped world" or "middle income countr*" or "middle income nation?" or "middle income population?" or "low income countr*" or "low income nation?" or "low income population?" or "lower income countr*" or "lower income nation?" or "lower income population?" or "underserved countr*" or "underserved nation?" or "underserved population?" or "underserved world" or "under served countr*" or "under served nation?" or "under served population?" or "under served world" or "deprived countr*" or "deprived nation?" or "deprived population?" or "deprived world" or "poor countr*" or "poor nation?" or "poor population?" or "poor world" or "poorer countr*" or "poorer nation?" or "poorer population?" or "poorer world" or "developing econom*" or "less developed econom*" or "lesser developed econom*" or "under developed econom*" or "underdeveloped econom*" or "middle income econom*" or "low income econom*" or "lower income econom*" or "low gdp" or "low gnp" or "low gross domestic" or "low gross national" or "lower gdp" or "lower gnp" or "lower gross domestic" or "lower gross national" or lmic or lmics or "third world" or "lami countr*" or "transitional countr*" or "emerging economies" or "emerging nation?" or "low-resource setting*" or "low-resource nation*" or "low-resource countr*" or "resource-limiting setting*" or "resource-limiting nation*" or "resource-limiting countr*"):ti,ab,kw
**#20**	#6 and #10 and #18 and #19

Appendix C: Eligibility criteria

Table 8**: Eligibility criteria for this review**

Category	Inclusion criteria	Exclusion criteria
**Population**	Preterm and term newborns (in the first 28 days after birth)	Infants, children, and adults
**Intervention**	Any intervention, policy, or strategy designed to promote antimicrobial stewardship and/or mitigate the development and spread of antimicrobial resistance	Studies not reporting on an intervention, policy, or strategy related to antimicrobial resistance prevention or reduction
**Comparator**	Standard practices, or no intervention	Any other comparator
**Outcome**	Studies reporting at least one of the following primary or secondary outcomes:<br>Primary outcomes:<br>• All-cause neonatal mortality<br>• Lab confirmed and suspected early onset sepsis (community or hospital onset)<br>• Lab confirmed and suspected late onset sepsis (community or hospital onset)<br>• Localized infections (e.g., omphalitis, urinary tract infection, meningitis)<br>• Localized infections due to multidrug-resistant organisms<br>• Confirmed blood stream infections<br>• Confirmed blood stream infections due to multidrug-resistant organisms<br>• Colonization with multidrug-resistant bacteria<br>Secondary outcomes:<br>• Duration of antibiotic therapy<br>• Proportion of neonates receiving any antibiotic<br>• Length of hospital stay (inpatient newborns)	Studies reporting only on behaviour change or outcomes not related to the health of the newborn, such as handwashing practices or kangaroo mother care

	• Use of WHO access, watch, and reserve antimicrobials	
**Study design**	Randomized trials, quasi-randomized trials, observational studies, program evaluations, or implementation studies	Reviews, animal studies, conference abstracts, or modelling studies
**Study setting**	Studies conducted in facility- or community-based LMIC settings, as defined by World Bank cut-offs at the time of search	Studies conducted in HIC settings, as defined by World Bank cut-offs at the time of search
**Report characteristics**	Papers describing the results of primary research both peer reviewed and grey literature	Protocols, conference abstracts, editorials, reviews, or opinion pieces
**Publication date**	2000 to present	Studies with a publication date before 2000
**Publication language**	Non-English studies will be included	Records that are not identifiable using database searches with English keywords
**Publication status**	Fully published or in-press	Studies under review or reports from trials still underway

Abbreviations: HIC, high-income country; LMIC, low- and middle-income country; WHO, World Health Organization

Appendix D: Eligibility criteria, outcomes defined

Table 9: **Outcomes defined according to our eligibility criteria**

Outcome	Definition
**Primary outcomes**	
**All-cause neonatal mortality**	All-cause neonatal mortality is defined as death from all causes during the first 28 days of life.
**Stillbirth**	Stillbirth is defined as the number of babies born with no signs of life at 28 weeks or more of gestation.
**Infection-attributable neonatal mortality**	Infection-attributable neonatal mortality is defined as death within 3 days of infection onset or blood culture during the first 28 days of life.
**Early onset sepsis (EOS)**	EOS is defined as laboratory confirmed or clinically suspected sepsis occurring at <72 hours of life.<br><br>*Laboratory Confirmation & Clinical Suspicion of EOS*<br>Confirmed EOS may be determined using C-reactive protein and procalcitonin, blood cultures, or molecular methods (i.e., PCR). Confirmed EOS determined by a positive laboratory test result or determined by culture-positive sepsis status at <72 hours of life. Suspected EOS may be pragmatically decided by clinicians' choice to prescribe an antibiotic based on clinical suspicion of sepsis in the absence of confirmed diagnosis.<br><br>*Acquisition of EOS*<br>EOS may be acquired through the community setting (home births) or through the hospital setting (facility births). Facility births may be further categorized as "admitted from birth at this facility", "referred from another facility", or "referred from home"[1]. If specifying place of birth as a variable, similar categories of "born at this facility", "born at another facility", or "born at home" could be used.
**Late onset sepsis (LOS)**	LOS is defined as laboratory confirmed or clinically suspected sepsis occurring at ≥72 hours of life.<br><br>*Laboratory Confirmation & Clinical Suspicion of LOS*<br>Confirmed LOS may be determined using C-reactive protein and procalcitonin, blood cultures, or molecular methods (i.e., PCR). Confirmed LOS determined by a positive lab test result or determined by culture-positive sepsis status at ≥72 hours of life. Suspected LOS may be pragmatically decided by clinicians' choice to prescribe an antibiotic based on clinical suspicion of sepsis, in the absence of confirmed diagnosis.

	Acquisition of LOS<br>LOS may be acquired through the community setting (home births) or through the hospital setting (facility births). Facility births may be further categorized as "admitted from birth at this facility", "referred from another facility", or "referred from home" [1]. If specifying place of birth as a variable, similar categories of "born at this facility", "born at another facility", or "born at home" could be used.<br><br>*New and Recurrent Cases*<br>New cases of LOS are defined as infections identified >7 days after stopping antimicrobial treatment. Recurrent cases of late onset sepsis are defined as infections identified <7 days after stopping antimicrobial treatment.
**Localized infections (e.g., omphalitis, UTI, meningitis)**	Localized infection is defined as laboratory confirmed infection affecting a single body part or organ (e.g., umbilical cord or urinary tract) during the first 28 days of life. Localized infections are identified through a culture or tissue sample from the affected organ or body part. Where possible, localized infections by day during the first week of life (days 0-6) will be presented.
***Mycobacterium tuberculosis* infection**	Infection with *Mycobacterium tuberculosis* (*M. tuberculosis*) is defined as laboratory confirmed *M. tuberculosis* infection during the first 28 days of life.<br><br>*Laboratory Confirmation of M. tuberculosis Infection*<br>Laboratory confirmation of *M. tuberculosis* infection occurs through the result of tuberculin skin test (TST), IFN-γ release assays (IGRAs), chest radiograph, or from bodily fluid or tissue samples for acid-fast bacteria (AFB) smear or culture.<br><br>*Acquisition of M. tuberculosis*<br>*M. tuberculosis* may be acquired in the community or the facility setting through airborne transmission of aerosolized droplets.
**Drug-resistant *Mycobacterium tuberculosis* infection**	Infection with drug-resistant *Mycobacterium tuberculosis* (*M. tuberculosis*) is defined as laboratory confirmed drug-resistant *M. tuberculosis* infection during the first 28 days of life. Drug resistance is defined as *M. tuberculosis* that is untreatable with one or more antibiotics, including multidrug-resistant tuberculosis (MDR TB) and extensively drug-resistant tuberculosis (XDR TB).

	Laboratory Confirmation of Drug-Resistant TB<br>Laboratory confirmation of drug-resistant *M. tuberculosis* may be determined through drug susceptibility testing, molecular testing methods, or culture results.<br><br>*Acquisition of Drug-Resistant M. tuberculosis*<br>*M. tuberculosis* may be acquired in the community or the facility setting through airborne transmission of aerosolized droplets.<br><br>*MDRO Outbreaks*<br>Multidrug-resistant infections can be further specified as outbreak-related, where outbreaks are defined as the occurrence of cases with a frequency in excess of normal expectancy in a defined population.
**Localized infections due to multidrug-resistant organisms (MDROs)**	Multidrug-resistant localized infections are defined as laboratory confirmed infections affecting a single organ or body part during the first 28 days of life, in which the organism is resistant to one or more classes of antimicrobial agents. Multidrug-resistant organisms of interest include methicillin-resistant *Staphylococcus aureus* (MRSA), vancomycin-resistant enterococci (VRE), extended-spectrum beta-lactamase-producing Enterobacterales, carbapenem-resistant Enterobacteriaceae (CRE), and *Klebsiella pneumoniae* carbapenemase (KPC).<br><br>*Laboratory Confirmation of Multidrug-Resistant Localized Infection*<br>Multidrug-resistant organisms (MDROs) are identified through lab cultures (tissue or urine sample; nasal and/or rectum swab), or antimicrobial susceptibility testing (AST), depending on laboratory testing resources and where the infection is located.<br><br>*Acquisition of Multidrug-Resistant Localized Infection*<br>MDROs may be acquired in the community or healthcare facility setting. MDROs are commonly spread through direct person to newborn contact or through newborn contact with a contaminated surface.<br><br>*New and Recurrent Multidrug-Resistant Localized Infection*<br>New localized infections due to multidrug-resistant organisms are defined as infections identified >7 days after stopping antimicrobial treatment. Recurrent localized infections due to multidrug-resistant organisms are defined as infections identified <7 days after stopping antimicrobial treatment.

	MDRO Outbreaks<br>Multidrug-resistant localized infections can be further specified as outbreak-related, where outbreaks are defined as the occurrence of cases with a frequency in excess of normal expectancy in a defined population.
**Confirmed bloodstream infections**	Neonatal bloodstream infections are defined as any infections detected in the bloodstream during the first 28 days of life and laboratory confirmed through blood culture, excluding contaminated blood cultures.
**Confirmed central-line-associated bloodstream infections (CLABSIs)**	Central-line associated bloodstream infections (CLABSIs) are defined as infections which enter the blood through a newborn's central line (i.e., catheter) during the first 28 days of life and are laboratory confirmed through blood culture, excluding contaminated blood cultures. CLABSIs are a common type of healthcare-associated infection (HAI).
**Confirmed ventilator-associated pneumonias (VAPs)**	Ventilator-associated pneumonias (VAPs) are defined as pneumonia which enters the lungs through a ventilator during the first 28 days of life and are laboratory confirmed through radiographic or culture methods. VAPs are another common type of healthcare-associated infection (HAI).
**Confirmed bloodstream infections due to MDROs**	Multidrug-resistant blood stream infections are defined as the presence of multidrug-resistant organisms causing infection in the bloodstream, confirmed by blood culture, and excluding contaminated blood cultures. Multidrug-resistant organisms cause infections that cannot be treated with one or more classes of antimicrobial agents.<br><br>*New and Recurrent Multidrug-Resistant Bloodstream Infections*<br>New bloodstream infections due to multidrug-resistant organisms is defined as infections identified >7 days after stopping antimicrobial treatment. Recurrent bloodstream infections due to multidrug-resistant organisms is defined as infections identified <7 days after stopping antimicrobial treatment.<br><br>*MDRO Outbreaks*<br>Multidrug-resistant bloodstream infections can be further specified as outbreak-related, where outbreaks are defined as the occurrence of cases with a frequency in excess of normal expectancy in a defined population.

**Colonization with multidrug-resistant bacteria**	Colonization with multidrug-resistant bacteria is defined as the presence of multidrug-resistant bacteria in or on the newborn's body without causing symptoms of infection or illness.
**Secondary outcomes**	
**Duration of antibiotic therapy**	Duration of antibiotic therapy is defined as the total number of days an antibiotic treatment was prescribed. If reported, duration of antibiotic therapy may be given as days of therapy (DOT).
**Proportion of neonates receiving any antibiotic**	Proportion of neonates receiving antibiotic is defined as the number of neonates who received any antibiotic treatment, and the denominator could be the number of neonates in the study population or the number of neonates being treated with any antibiotic, depending on what is reported.
**Length of hospital stay (inpatient newborns)**	Length of hospital stay is defined as the number of hours or days newborns remained admitted to hospital after birth. Where reported, reasons for neonatal hospital stay will be presented.
**Use of WHO Access, Watch, or Reserve antimicrobials**	The number of antimicrobials used during the first 28 days of life classified according to the World Health Organization's Access, Watch, or Reserve (AWaRe) categories. Where available, the number of antimicrobials used by day for the first week of life (days 0-6) classified according to the World Health Organization's Access, Watch, or Reserve categories will be presented.<br><br>*AWaRe Classification*<br>Access group antibiotics are usually first or second choice antibiotics which target many commonly encountered pathogens. Access group antibiotics are predominantly narrow spectrum. Most bacteria have lower levels of resistance potential to treatment with Access group antibiotics compared to antibiotics in the other classification groups. Watch group antibiotics include first or second choice antibiotics, and this class of antibiotics act against bacteria with a higher risk of resistance selection potential compared to the Access group. Watch group antibiotics are predominantly broad-spectrum. Reserve group antibiotics are antibiotics which should be used as a last resort and saved for use against confirmed or suspected multidrug-resistant organisms (MDROs).

Abbreviations: PCR, polymerase chain reaction; WHO, World Health Organization

Appendix E: List of excluded studies with reasons

Table 10: **List of excluded studies with rationale**

No.	Excluded study	Study title	Exclusion reason
**1**	Araujo da Silva 2020[2]	Patterns of antimicrobial consumption in neonatal and pediatric intensive care units in Germany and Brazil	Study compared outcome data from Germany (a high-income country) and Brazil (an upper-middle income country)
**2**	Azab 2015[3]	Reducing ventilator-associated pneumonia in neonatal intensive care unit using "VAP prevention bundle": a cohort study	Intervention was not designed for resistance/stewardship purposes but to decrease ventilator-associated pneumonia rate
**3**	Balla 2018[4]	Decreasing central line-associated bloodstream infections through quality improvement initiative	Intervention was not designed for resistance/stewardship purposes but to decrease bloodstream infections and central line-associated blood stream infections rates
**4**	Bazan 2025[5]	Long-lasting effects of control measures on trends in incidence in neonatal late-onset sepsis due to multiresistant bacteria in a Brazilian neonatal unit	Only long-term sepsis trends reported since intervention implementation (i.e., no eligible comparator)
**5**	Dramowski 2021[6]	NeoCLEAN: a multimodal strategy to enhance environmental cleaning in a resource-limited neonatal unit	Intervention was not designed for resistance/stewardship purposes but to improve hospital cleaning practices
**6**	Hussain 2020[7]	CLABSI reduction using evidence based interventions and nurse empowerment: a quality improvement initiative from a tertiary care NICU in Pakistan	Intervention was not designed for resistance/stewardship purposes but was meant to reduce facility central line-associated blood stream infections rates
**7**	Iqbal 2025[8]	Assessment of an Antimicrobial Stewardship Program for Enhancing Clinical Knowledge in Neonatal Care Settings With High Antimicrobial Resistance	Reported only on healthcare worker behaviour change or outcomes not related to the health of the newborn (i.e., wrong outcomes)

**8**	Johnson 2022[9]	Implementation of the comprehensive unit-based safety program to improve infection prevention and control practices in four neonatal intensive care units in Pune, India	Intervention was not designed for resistance/stewardship purposes but to improve infection prevention and control practices
**9**	Khan 2025[10]	Assessing the Use of Neonatal Sepsis Guidelines and Antibiotic Prescription With Large-Scale Prospective Data From Zimbabwe and Malawi	Compared neonatal outcomes among those who should have received antibiotics as per guideline with those who actually received them (i.e., wrong comparison)
**10**	Kulali 2019[11]	Impact of central line bundle for prevention of umbilical catheter-related bloodstream infections in a neonatal intensive care unit: A pre-post intervention study	Intervention was not designed for resistance/stewardship purposes but to prevent bloodstream infections associated with umbilical venous catheters
**11**	Lloyd 2025[12]	Theoretical impact of a bedside decision-making tool on antibiotic use for suspected neonatal healthcare-associated infection: an observational study	Compared observed versus theoretical impact of the intervention on antibiotic length of therapy (i.e., a predictive modelling study)
**12**	Mwananyanda 2019[13]	Preventing bloodstream infections and death in Zambian neonates: Impact of a low-cost infection control bundle	Intervention was not designed for resistance/stewardship purposes but to prevent hospital-associated sepsis and death
**13**	Navoa-Ng 2011[14]	Device-associated infections rates in adult, pediatric, and neonatal intensive care units of hospitals in the Philippines: International Nosocomial Infection Control Consortium (INICC) findings	Intervention was not designed for AMR-related reasons but to decrease device-associated infections only
**14**	Picheansathian 2008[15]	The effectiveness of a promotion programme on hand hygiene compliance and nosocomial infections in a neonatal intensive care unit	Intervention was not designed for AMR-related reasons but to improve hygiene and prevent hospital-acquired infections
**15**	Rahim 2009[16]	Reducing nosocomial infection in neonatal intensive care: an intervention study	Intervention was not designed for resistance/stewardship purposes but to prevent hospital-acquired infections

**16**	Resende 2011[17]	Reduction of catheter-associated bloodstream infections through procedures in newborn babies admitted in a university hospital intensive care unit in Brazil	Intervention was not designed for resistance/stewardship purposes but to prevent catheter-associated bloodstream infections
**17**	Rosenthal 2012[18]	Findings of the international nosocomial infection control consortium (INICC), part ii: impact of a multidimensional strategy to reduce ventilator-associated pneumonia in neonatal intensive care units in 10 developing countries	Intervention was not designed for resistance/stewardship purposes but to decrease ventilator-associated pneumonia rate
**18**	Rosenthal 2013[19]	Findings of the international nosocomial infection control consortium (INICC), part iii: effectiveness of a multidimensional infection control approach to reduce central line-associated bloodstream infections in the neonatal intensive care units of 4 developing countries	Intervention was not designed for resistance/stewardship purposes but to decrease central line-associated bloodstream infection rate
**19**	Tagare 2009[20]	Routine antibiotic use in preterm neonates: a randomised controlled trial	Intervention was not designed for resistance/stewardship purposes but to decrease sepsis incidence
**20**	Tran 2018[21]	Early essential newborn care is associated with reduced adverse neonatal outcomes in a tertiary hospital in Da Nang, Viet Nam: a pre-post- intervention study	Intervention designed not for AMR reduction purposes but to improve clinical practices, reduce NICU admissions, admissions with hypothermia and sepsis, and increase rates of exclusive breastfeeding and kangaroo mother care
**21**	Urzúa 2017[22]	Strategies to reduce infections and antibiotic use and its effects in a neonatal care unit	Study was conducted in Chile, a high-income country
**22**	Wang 2015[23]	Prevention of peripherally inserted central line-associated blood stream infections in very low-birth-weight infants by using a	Intervention was not designed for resistance/stewardship purposes but to prevent catheter-associated infections

		central line bundle guideline with a standard checklist: a case control study

Appendix F: WHO EMLc AWaRe classification

Table 11: **Description and examples of Access, Watch, and Reserve group antibiotics included in the World Health Organization (WHO) Essential Medicines List for Children (EMLc)[24]**

**Access group antibiotics**	
• **Amikacin**<br>• **Amoxicillin**<br>• **Ampicillin**<br>• **Amoxicillin–clavulanic acid**<br>• **Benzathine benzylpenicillin**<br>• **Benzylpencillin**<br>• **Cefazolin**<br>• **Chloramphenicol**<br>• **Clindamycin**<br>• **Cloxacillin**<br>• **Doxycycline**<br>• **Gentamicin**<br>• **Metronidazole**<br>• **Nitrofurantoin**<br>• **Phenoxymethylpencillin**<br>• **Procaine pencillin**<br>• **Spectinomycin**<br>• **Sulfamethoxazole–trimethoprim**	This group includes antibiotics and antibiotic classes that have activity against a wide range of commonly encountered susceptible pathogens while showing lower resistance potential than antibiotics in Watch and Reserve groups. Access antibiotics should be widely available, affordable and quality-assured to improve access and promote appropriate use.<br>Selected Access group antibiotics are included on the WHO Essential Medicines List for Children (EMLc) as essential first-choice or second-choice empirical treatment options for specific infectious syndromes.[25]
**Watch group antibiotics**	
• **Azithromycin**<br>• **Cefixime**<br>• **Ceftriaxone**<br>• **Cefotaxime**	This group includes antibiotics and antibiotic classes that have higher resistance potential and includes most of the highest priority agents among the Critically Important Antimicrobials

• **Ceftazidime**<br>• **Cefuroxime**<br>• **Vancomycin**<br>• **Ciprofloxacin**<br>• **Clarithromycin**<br>• **Meropenem**<br>• **Piperacillin–tazobactam**	(CIA) for Human Medicine and/or antibiotics that are at relatively high risk of selection of bacterial resistance. Watch group antibiotics should be prioritized as key targets of national and local stewardship programmes and monitoring. Selected Watch group antibiotics are included on the WHO EMLc as essential first-choice or second-choice empirical treatment options for a limited number of specific infectious syndromes.[25]
**<u>Reserve group antibiotics</u>**<br>• **Fosfomycin (intravenous)**<br>• **Linezolid**<br>• **Colistin**<br>• **Polymyxin B**<br>• **Ceftazidime–avibactam**<br>• **Meropenem–vaborbactam**<br>• **Plazomicin**	This group includes antibiotics and antibiotic classes that should be reserved for treatment of confirmed or suspected infections due to multi drug-resistant organisms, and treated as “last-resort” options. Their use should be tailored to highly specific patients and settings, when all alternatives have failed or are not suitable. They could be protected and prioritized as key targets of national and international stewardship programmes, involving monitoring and utilization reporting, to preserve their effectiveness. Selected Reserve group antibiotics are included on the WHO EMLc when they have a favourable risk-benefit profile and proven activity against “Critical Priority” or “High Priority” pathogens identified by the WHO Priority Pathogens List, notably carbapenem-resistant Enterobacteriaceae.[25]

Appendix G: Characteristics of included studies

Table 12: **Characteristics of included studies summarized**

No.	Author year	Study design	Setting	Level of care	Participants	Intervention classification	Intervention(s) detailed	Outcome(s) reported
**1**	**Agarwal 2021**[26]	Quasi-experimental (uncontrolled before-and-after PDSA QI study)	Jaipur, India – urban	Facility – level 3 NICU at tertiary referral hospital	N=2292; preterm and term outborn neonates (in the first 28 days of life) admitted to NICU	Regulation, education, and optimization	-Interventions included: creating a unit protocol for sepsis management and antibiotic prescribing; educating nurses and doctors through presentations and the display of reminder posters regarding sepsis guidelines, antibiotic abuse, and emerging antibiotic resistance; establishing 48-hour and 7-day check points for starting and early stoppage of antibiotics so as to restrict initiation and stop therapy in non-septic babies (i.e., If blood culture was negative and two C-reactive protein tests were negative 24 hours	Neonatal mortality; NEC; AUR; AUR in culture-negative newborns; AUR for vancomycin; prolonged antibiotic use

							apart, the patient being asymptomatic, then antibiotics would be stopped. Similarly, antibiotics in culture-negative and screen-positive patients were not given for more than 7 days.); formulating a specific protocol to start vancomycin; reviewing the yearly antibiotic policy as per antibiogram; epidemiological and microbiological surveillance showing the emergence of MDROs in the unit; and regular audit with feedback system in monthly statistical meetings<br>-Study duration: 10 months	
**2**	**Akintan 2024[27]**	Observational (serial cross-section	Lagos, Nigeria – urban	Facility – tertiary referral hospital	N=90 neonates; inborn and outborn patients aged 0-17 years	Regulation	-Antibiotic guidelines for the pediatric department; an antibiotic policy document developed	Patients treated for at least one HAI; patients diagnosed with sepsis among

		al study using point prevalence surveys)			admitted before 8 am to pediatric ward and receiving antibiotic medications were surveyed (data disaggregated for neonates and children)		by the AMS committee; prospective audit with feedback<br>-Duration: 4 years	those prescribed antibiotics; number of patients prescribed at least one antimicrobial; use of WHO access, watch, and reserve antimicrobials
**3**	**Alp 2015[28]**	Quasi-experimental (uncontrolled before-and-after study)	Central Anatolian region, Turkey – urban/rural not reported	Facility – level 3 NICU at tertiary referral hospital	N=27661; neonates – inborn/outborn not reported	Regulation and education	-Multimodal HH education promotion campaign and infection control program including the provision of alcohol-based hand rub at every bedside, chlorhexidine containing soap, and HH compliance audits with feedback<br>-Duration: 4 years	HAI; CAUTI; CLABSI; VAP; multiple drug-resistant *A. baumanni;* multiple drug-resistant *K. pneumoniae;* multiple drug-resistant *P. aeruginosa;* multiple drug-resistant gram-negative bacteria; MRSA; mean length of NICU stay
**4**	**Bassiouny 2020[29]**	Quasi-experimental	Cairo, Egypt – urban	Facility – tertiary (surgical	N=210; outborn neonates admitted to the	Regulation, education,	-Antimicrobial stewardship program to control and limit	Neonatal mortality; SSI; resistant strains

		(uncontrolled before-and-after study)	NICU at specialized pediatric hospital)	ICU for surgical interventions	and optimization	antibiotic misuse by applying new guidelines to reduce infection rates; decreasing hospital drug costs; and reducing the lengths of hospital stay in the ICU. The program also initiated training on good sampling to avoid poly-microbial nature of the samples and a pediatric clinical pharmacist attended patient care rounds to reinforce key ASP concepts; and they introduced preliminary reporting of direct gram-stained films before final reporting of sensitivity results; and automation to decrease turnaround time.<br>-Duration: 6 months	isolated (from wound cultures, from blood cultures, from other cultures); blood culture isolates (*Klebsiella* spp., *Candida* spp., *Acinetobacter* spp., CoNS, *E. coli*, *Enterobacter* spp., MRSA, alpha and beta hemolytic *Streptococcus* spp., *Enterococcus* spp., *Pseudomonas* spp.); length of hospital stay; mean DOT (amoxicillin/clavulanate, ampicillin sulbactam, gentamicin, amikacin, cefotaxime, imipenem, meropenem,

								metronidazole, fluconazole, vancomycin, polymyxin, cefuroxime, ciprofloxacin, teicoplanin, linezolid, levofloxacin, piperacillin-tazobactam, amphotericin, ceftriaxone, trimethoprim-sulfamethoxazole, cefepime)
**5**	**Chimhini 2020[30]**	Quasi-experimental (uncontrolled before-and-after study)	Harare, Zimbabwe – urban	Facility – tertiary (accept nation-wide surgical referrals)	N=648; all admissions to the neonatal unit – inborn and outborn included	Regulation and education	-Audit with feedback; a training programme, including consultant-led unit induction sessions for interns and weekly unit meetings of nursing and medical staff where key messages were disseminated such as discontinuing empiric prescriptions of prophylactic oral amoxicillin on admission for babies	Death; diagnosis of sepsis at admission; duration of admission in days (median)

							not on IV antibiotics and at discharge; neonatal sepsis guidelines displayed on the unit; regular antimicrobial stewardship consultant-led ward-rounds introduced to reinforce differentiating between babies 'at risk of' versus 'with' clinically-suspected sepsis such that asymptomatic babies with one risk factor for sepsis were to be admitted for observation of vital signs for 24 hours before starting antibiotics<br>-Duration: 2 months	
**6**	**Chu 2023[31]**	Quasi-experimental (uncontrolled before-and-	Hunan Province, China – urban	Facility – tertiary (level 4 perinatal center)	N=249; all eligible VLBW infants (birth weight <1500 grams) born from 1 January 2014 to 31 December 2016	Regulation, education, and optimization	-Introduction of guidelines for diagnosing and treating early- and late-onset sepsis; provider education for the diagnosis and treatment of sepsis in	Death; LOS; NEC; antibiotic usage rate; antibiotic usage duration; length of hospitalization

		after study)*			– inborn/outborn not reported		VLBW infants; and ASP emphasizing restrictive use of empirical antibiotics<br>-Duration: 3 years	
**7**	**Dramowski 2024[32]**	Quasi-experimental (uncontrolled before-and-after study)	South Africa – urban/rural not reported	Facility – mixed primary, secondary, and tertiary	N=565 neonates enrolled from 14 neonatal admission units (7 public sector, 7 private sector)	Regulation, education, and optimization	NeoAMS intervention:<br>-A multidisciplinary collaborative method known as the Breakthrough Series, incorporating 7 online AMS real-time training and progress feedback sessions using standardized templates, weekday pharmacist audit of neonatal antibiotic prescriptions with real-time, and face-to-face feedback and AMS recommendations given to the treating clinician and the multidisciplinary team.<br>-Each team was provided with a previously validated neonatal AMS training toolkit developed by the study faculty. The	Mean antibiotic LOT

			toolkit aimed to provide non-specialized pharmacists with essential skills and tools for neonatal AMS, including primers on neonatal sepsis, culture-negative sepsis, and recommended duration of antibiotic therapy. Weekly NeoAMS study team meetings were held, with completion of a site baseline survey and participation in several interactive, online learning sessions.<br>-Study pharmacists were required to spend at least one hour each weekday auditing antibiotic prescriptions and providing feedback on AMS recommendations to the treating clinician/s in the neonatal wards and/or neonatal intensive care unit	

			(NICU), collectively referred to as the neonatal unit. Recommendations to the prescribers included, e.g., to stop antibiotics for culture-negative sepsis after five days, improve hangtime, make dose/dosing frequency changes, perform therapeutic drug monitoring, change therapy for bug-drug mismatch, or de-escalate to a narrow spectrum antibiotic agent.<br>-All NeoAMS team members participated in interdisciplinary discussions regarding antibiotic use and microbiology reports. When encountering difficult AMS cases or in cases of disagreement on AMS recommendations, the site microbiologist was	

							consulted to provide guidance on stopping, switching, de-escalating or escalating antibiotic therapy.<br>-Duration: 20 weeks	
**8**	**El-Baky 2020**[33]	Quasi-experimental (uncontrolled before-and-after study)	Minia, Egypt – urban	Facility - tertiary	N=1079; term and pre-term neonates with nosocomial infection – inborn/outborn not reported	Regulation and optimization	-A review of infection control measures and antibiotic policy was done; an infection control checklist covering disinfection, cleaning, hand hygiene, and visitors was instituted; and a culture-based antibiotic policy with daily and weekly follow-up for the implementation of the recommended infection control guidelines was applied. The antibiotic policy specified ampicillin and cefotaxime as first line therapy for EOS, and vancomycin and imipenem for LOS and piperacillin-tazobactam	Neonatal mortality due to nosocomial infection; blood sample isolates (*E. coli, S. aureus*, CoNS, *Streptococcus pneumoniae*, *K. pneumoniae*, *Enterobacter* spp., *Citrobacter* spp., *A. baumannii*, *P. aeruginosa*); length of hospital stay (3-7 days); length of hospital stay (7+ days)

							and linezolid as second line therapy.<br>-Duration: 9 months	
**9**	**Farias-Filho 2024[34]**	Quasi-experimental (uncontrolled before and after study)	Rio de Janeiro, Brazil – urban	Facility – tertiary	N=1,783 newborns in 60-bed NICU of private maternity hospital – mostly inborn	Regulation, education, and optimization	Implementation of antimicrobial stewardship interventions were delivered in stages:<br>-In Stage I, the profile of newborns was mapped.<br>-In Stage II, pharmacotherapeutic protocols for parenteral medications were developed.<br>-In Stage III, parenteral medication management for the Neonatal Intensive Care Unit was implemented.<br>-In Stage IV, an electronic dosage calculator was integrated into the Computerized Physician Order Entry.<br>-In Stage V, physicians and professionals responsible for	Number of antimicrobial prescription orders; incidence of prescribing errors

							dispensing medications were trained by clinical pharmacists.<br>-Duration: 14 months	
**10**	**Feng 2022[35]**	Quasi-experimental (uncontrolled before-and-after study)	Chongqing, China	Facility – tertiary referral hospital	N=7705; neonates with suspected infections, pneumonia, CNS with GA or corrected GA ≥ 34 weeks and length of hospital stay ≥ 24 hours were included – inborn/outborn not reported	Regulation, education, and optimization	-Evidence-based antibiotic stewardship was applied to neonates with suspected infections, pneumonia, and CNS. Interventions included recording clinical manifestations on an observation form for neonatal infections; antibiotic therapy of no more than 48 hours for suspected infections, and 5 days for pneumonia and CNS; prospective audits, staff education on AMS, and feedback.<br>-Duration: 1 year	Neonatal mortality; infection with MDROs; LOS; NEC; total mean DOT; antibiotic exposure; DOT (culture-negative EOS, culture-negative LOS, pneumonia, culture-proven infections); antibiotic use by indication (culture-negative EOS, culture-negative LOS, pneumonia, culture-proven infections); duration of antibiotic use (<2 days, ~5 days, >5 days); DOT (cephalexin, cefazoxime,

								penicillin, amoxicillin clavulanate, piperacillin, meropenem, other cephalosporins, metronidazole, vancomycin, ticoranin, other antibiotics); median duration of antibiotic days in treatment of pneumonia; newborns treated in ≤ 5 days for pneumonia; median duration of antibiotic days in newborns with CNS; newborns with CNS treated in ≤ 5 days; median length of stay; re-initiation of antibiotics within 14 days
**11**	**Garpvall 2021[36]**	Quasi-experi mental	Hanoi, Vietna	Facility - tertiary	N=323 newborns	Regulation	-Continuous CRE screenings via fecal sample (re-screening	CRE acquisition (i.e., colonization

		(uncontrolled before-and-after study)	m – urban				every 7 days of ICU care for the CRE-negative cohort) and cohort care (each ward divided into three sections: for newly arrived patients awaiting screening results; for patients screened CRE-negative and for patients screened CRE-positive) -Duration: 3 months	during hospitalization)
**12**	**Gill 2009**[37]	Quasi-experimental (uncontrolled before-and-after study)	Manila, Philippines – urban	Facility – tertiary (2 level 3 NICUs, one public teaching hospital and one obstetrical and gynecological charity hospital)	N=1827; inborn neonates admitted to NICU 1 or 2 during the study period	Regulation, education, and optimization	-Simplified package of infection control measures consisting of ethanol hand rub provision and staff hand hygiene assessments (i.e., one-hour observations were performed intermittently during the day or night shift at a 2:1 ratio); daily infection control checklists for NICU teams to select one checklist item per day, given the large number of checklist	Neonatal mortality; colonization (all drug-resistant, MRSA, VRE, all drug-resistant gram-negative bacilli, all enteric gram-negative bacilli, all non-enteric gram-negative bacilli, ceftazidime-resistant gram-negative bacilli, gentamicin-resistant gram-negative bacilli,

			items overall (e.g., items included asking staff to re-evaluate empirical antibiotic therapy); NICU attending physicians' monthly infection control checklists; preparatory workshops for key staff at each unit including lectures on hand hygiene and infection control, interactive case discussions, and a collaborative critique of a video of patient-staff interactions and infection-control activities at one of the NICUs; antimicrobial susceptibility testing using Kirby-Bauer disk diffusion; quality control procedures established in the laboratory using standard American Type Culture Collection strains; screening	gram-negative bacilli resistant to both ceftazidime and gentamicin, MRSA); bloodstream isolates (all gram-positive cocci, MRSA, VRE, CoNS, all gram-negative bacilli, all enteric gram-negative bacilli, all non-enteric gram-negative bacilli)

			for drug-resistant gram-negative bacilli and gram-positive cocci for all neonates according to a standard schedule (culture of perianal swab and/or stool specimens for surveillance of gram-negative bacilli and VRE, and umbilical and anterior nare swab specimens for surveillance of MRSA). Surveillance for drug-resistant gram-negative bacilli commenced within 16 hours after admission to NICU (on day 0 to detect colonization prior to admission) and was performed on days 2 and 7, every 7 days thereafter, and on the day of hospital discharge. The same schedule was used for screening for MRSA, omitting the day-0 cultures.	

							-Duration: 5-10 months	
**13**	**Graus 2022[38]**	Quasi-experimental (uncontrolled before-and-after PDSA QI study)	Lima, Peru - urban	Facility – level 3 NICU at tertiary pediatric subspecialty and surgery hospital	N=858; inborn neonate admissions to the NICU	Regulation, education, and optimization	-Antibiotic stewardship program for EOS in the NICU consisting of: blood culture collection standardization and reporting (volume of 1 mL drawn for every blood culture; culture data registry created; daily communication with the microbiology laboratory for timely information); optimal use of antibiotics (through staff education sessions including discussion of cases, capacity development in prevention and management of infections, and training in adequate antibiotic prescription and rationale for stewardship); discontinuation of antibiotics at 48 hours (standardized daily	AUR

			communication with microbiology laboratory to check culture results and establish a timeout or antibiotic use; after team discussion, if antibiotics were not discontinued by 48 hours when blood culture was negative, the rationale for that decision was documented in the medical record; for cases with positive culture results, the team determined appropriate antibiotic coverage); monthly monitoring and review of AUR (monthly meetings to review data on AUR and discuss further ideas for improvement; AUR information posted to main location for a visual display of AUR changes over time); education for new	

							residents and fellows and journal club meetings (educational sessions on neonatal sepsis, optimal antibiotic use and antibiotic stewardship were held; weekly journal club to review current evidence regarding the best approach to infants born to mothers with intrauterine infection, the approach to EOS, the use of biomarkers for the diagnosis of sepsis, etc.); and others (clinical practice guideline for EOS updated).<br>-Duration: 2 years	
**14**	**Huang 2019[39]**	Quasi-experimental (uncontrolled before-and-	Xiamen, China - urban	Facility – level 3 NICU at tertiary academic hospital	N=11277; outborn neonates admitted to the NICU	Regulation and education	-Bundle intervention measures were performed including screening for MRSA; isolation precautions (neonates with high risk factors for MRSA	HAI; healthcare-associated MRSA infection; hospital-acquired MRSA infection; community-

		after study)		were placed in a private room and screened; isolation methods also included alcohol disinfection of the bed, hanging blue contact isolation mark on the door and bed of isolation ward, "MRSA" sign on the patient's electronic medical record, and yellow button on the wristband)); training of HH (i.e., on proper prevention and how to best control MRSA transmission in a hospital setting, and HH activities such as the WHO's "Five Moments for Hand Hygiene" were adopted to improve compliance); and cleaning protocols and decontamination of the isolation ward (medical supplies of each neonate with MRSA	acquired MRSA infection

							were used solely for that patient (e.g., stethoscope); the bed unit was wiped by nurses with disinfectant three times daily)<br>-Duration: 1 year	
**15**	**Jain 2021**[40]	Quasi-experimental (uncontrolled before-and-after PDSA QI study)	Sewagram, India – rural	Facility – tertiary public teaching hospital	N not reported; inborn neonates	Regulation, education, and optimization	-Antibiotic stewardship program which included: introducing a department policy plan for starting antibiotics (Broad-spectrum antibiotics like ampicillin, or cefotaxime and gentamicin were standardized for empirical antimicrobial treatment for all admissions. Criteria for starting antibiotic, dose, dosing interval and duration were clearly mentioned. Vancomycin and amikacin were standardized as second-line for sick	Average antibiotic exposure rate; median antibiotic days per newborn

			babies. Preauthorisation was made mandatory for residents before starting antibiotics to stop the empirical antibiotic treatment policy.); adaptation of universal aseptic precautionary measures (e.g., frequent sanitization, minimum handling and use of nursing barriers, infection control nurse appointed to conduct hand washing drills, assess aseptic measures; hand washing was also taught to mothers visiting their babies in the NICU); discontinuation of antibiotics at 72 hours (a strict hard stop to all antibiotics beyond 72 hours and tracing of culture report telephonically by 72 hours if not validated	

			by then); streamlining/de-escalation of antibiotics (through team discussion to reach a consensus); dissemination of responsibilities (e.g., transferring responsibility for stopping antibiotics at 72 hours from medical residents to nursing staff; improvement of work culture in the NICU); confidence building of nursing staff and residents (two 45-minute sessions for team building activities to improve communication among all working staff and emphasize everyone’s role in preventing sepsis in the NICU; team meetings convened fortnightly with all NICU staff to discuss any issues together); and	

							continuous feedback with regular, weekly audits<br>-Duration: 6 months	
**16**	**Jinka 2017[41]**	Quasi-experimental (uncontrolled before-and-after study)	Bathalapalli, India – rural	Facility – NICU of tertiary hospital	N=2452; inborn and outborn neonates admitted to the NICU	Optimization	-An antibiotic consumption protocol for empirical therapy of neonatal sepsis was developed based on review of NICU blood culture susceptibility data (Ampicillin and gentamicin were considered as first-line for community-acquired infections, and a combination of amikacin and ciprofloxacin were considered if deterioration on first-line antibiotics occurred. For HAIs, a combination of amikacin and ciprofloxacin was first-line. Meropenem was recommended for empiric therapy only in very severe cases of HAIs. Third generation	Mean length of hospital stay; total DDDs; DDD (amikacin, ampicillin, chloramphenicol, ciprofloxacin, gentamicin, meropenem, piperacillin-tazobactam, vancomycin, colistin, 3rd generation cephalosporins), number of newborns (ampicillin or gentamicin, amikacin, ciprofloxacin, colistin, meropenem, piperacillin-tazobactam, 3rd generation cephalosporins);

							cephalosporins were recommended only when an intracranial infection was suspected. Empirical treatment choices were adjusted subsequently to narrow spectrum antibiotics whenever possible based on culture and antibiotic susceptibility results.)<br>-Duration: 1 year	overall number of newborns on antibiotics
**17**	**Kommalur 2021**[42]	Quasi-experimental (uncontrolled before-and-after QI PDSA study)	Bangalore, India – urban	Facility – tertiary government referral hospital	N=413; all neonates admitted to the preterm unit during the study period – inborn and outborn included	Regulation, education, and optimization	-Revision of antibiotic policy; strict management of antibiotics (e.g., first-line ampicillin and gentamicin strictly managed; third-line drug use was restricted only to culture-proven cases and empirical therapy with meropenem was stopped; empirical use of cephalosporins and prophylactic antifungal therapy, although not used routinely, was	Mortality rate; number of neonates with blood culture-positive sepsis; organisms isolated in blood culture; antibiotic usage expressed as DOT/1000 patient days

			stopped); diagnostic stewardship measures (e.g., early tracing of preliminary blood culture reports by 48 hours); no-prick policy for asymptomatic and stable preterm newborns; a lecture on antibiotic stewardship, case-based discussion, and weekly bedside rounds, especially for junior residents who wrote treatment orders; newborn classification based on signs and symptoms for careful management, also based on blood culture and C-reactive protein test results; de-escalation to the narrowest spectrum effective antibiotic in blood culture-positive cases; installation of a dashboard indicator for displaying census and number of infants receiving antibiotics	

							and stopping orders; daily antibiotic ward rounds; periodic audit with feedback<br>-Duration: 6 months	
**18**	**Konda 2021[43]**	Quasi-experimental (uncontrolled before-and-after PDSA QI study)	Hyderabad, India – urban	Facility – tertiary government teaching hospital	N not reported; babies admitted to inborn NICU	Regulation, education, and optimization	-QI approach to decrease unindicated usage of antibiotics consisting of: revision of antibiotic policy based on culture sensitivity data (and reinforced by displaying the policy in the unit as a reminder, copies sent to staff's phones electronically via WhatsApp); staff training sessions on stewardship (i.e., six 30-minute meetings conducted over one week with staff asked to attend in turns so that patient care was not hampered. The importance and need for antibiotic stewardship was discussed, especially with regard to	Newborns with unindicated antibiotic usage

			switching to the narrowest spectrum drug available or discontinuation whenever feasible.); documentation required for initiation of antibiotic use (i.e., initiation of a new antibiotic required compulsory documentation of the indication or justification in the case records); implementation of a “lock model” where approval is required for use of second- and third-line drugs; de-escalation considered every 72 hours after drug initiation for neonates with sepsis-like symptoms; and continuous once weekly team meetings, feedback, and motivation<br>-Duration: 10 weeks	

**19**	**Landre-Peigne 2021**[44]	Quasi-experimental (uncontrolled before-and-after study)	Dakar, Senegal – urban	Facility – secondary care NICU at military teaching hospital	N=273; neonates admitted to NICU – inborn/outborn not reported	Regulation and optimization	-Multi-faceted hospital infection control program introduced new measures including: clustering of nursing care such that patients rather than duties were allocated; establishing an algorithm for empirical therapy of suspected EOS; setting limits for invasive care procedures such as peripheral venous catheters and nasogastric feeding tubes to minimize the development of nosocomial bloodstream infections; and promoting the early discharge of neonates<br>-Duration: not reported	Nosocomial bloodstream infection; neonatal mortality; neonatal mortality after 48 hours; neonatal mortality due to nosocomial bloodstream infection; proven or possible EOS; number of neonates treated with antibiotics for suspected EOS; bacterial resistance; newborns treated with antibiotics for suspected EOS
**20**	**Lu 2019**[45]	Quasi-experimental (interrupted time	Shanghai, China – urban	Facility – level 3-4 NICU at tertiary children'	N=13540; outborn neonates who received antibiotics	Regulation, education and optimization	-SMAP summarized as: one team (SMAP team), two goals (safety and quality), three groups (administrative,	Neonatal mortality; LOS; NEC (Bell stage I); NEC (Bell stage ≥ II); multidrug-resistant

		series analysis comparing before and after implementation)		s referral hospital	during their hospital stay		supervision, and implementation), four strategies (audit, feedback, prior authorization, and point-of-prescription interventions), and five main measured outcomes. Antibiotics were divided into non-restricted (also called “first line”), such as penicillin and ampicillin, restricted (“second line”), such as third-generation cephalosporin, and special use or selected (“third line”) grades, such as meropenem and linezolid. Each grade is an exact match with the corresponding prescribing privileges of the physicians. In specific cases, such as during emergencies, physicians are permitted to use these special antimicrobials for no more than 1 day.	organism colonization; carbapenem-resistant *Enterobacteriaceae* infection; clinical or proven infection; culture-proven infection; early onset culture-proven infection; late onset culture-proven infection; pneumonia; congenital syphilis; skin or soft-tissue infection; perioperative prophylaxis; total DOT; antibiotic use [(DOT by grade: nonrestricted, restricted, selected), (DOT by indications: suspected EOS, suspected LOS, pneumonia, NEC

							Daily reviews of the electronic medical records of each patient in our NICU was performed, and an analysis of all antibiotic use was continued until discharge. A 48-hour automatic stop order was introduced, in which empirical antibiotic therapy was discontinued automatically unless reordered by a provider. Neonatologists and fellows were informed and specially trained about the SMAP interventions. -Duration: 1 year	(≥ stage II), CPS)]; newborns treated for pneumonia; newborns treated for pneumonia ≥ 5 days; newborns treated for CNS; newborns with CNS treated for ≥5 days; median length of hospital stay
**21**	**Maalouf 2023**[46]	Quasi-experimental (uncontrolled before-and-	Beirut, Lebanon – urban	Facility - level 4 NICU at tertiary referral hospital	N=716; inborn and outborn neonates admitted to the NICU and receiving intravenous or	Regulation, education, and optimization	-NS-ASP implemented using a multidisciplinary approach of interventions which included: the formation of a team to	EOS; median AUR; AUR of ampicillin; AUR of gentamicin; AUR of meropenem; AUR of vancomycin;

		after PDSA QI study)	intramuscular antimicrobials including antibacterials and antifungals during their hospitalization	monitor antibiotic usage, conduct patient chart reviews, deliver educational sessions to faculty and staff on the benefit of NS-ASP and harm of antibiotic overuse, and conduct biweekly audit and feedback rounds; establishment of guideline-based algorithms to guide treatment; implementation of an electronic health record system for more accurate drug administration and dispensing data; and a review of laboratory data, implementation of a hard-stop at 48 hours for antibiotics prescribed for suspected EOS, and daily Best Practice Alert messages sent to the prescribing team<br>-Duration: 5 years	mean length of hospital stay

**22**	**Nygren 2025**[47]	Quasi-experimental (uncontrolled before-and-after study)	Banjul, The Gambia - urban	Facility – tertiary, at the only referral hospital offering specialist care, dialysis, and intensive care in the country	N=518 neonates*<br><br>*Pre-intervention period described in separate publication by *Rahden 2025*[48]	Optimization	-Microbiological testing capabilities and capacity, and AMS initiatives, have been supported through increased blood culture and AST availability and increased use of microbiological diagnostics; AMS initiatives included reduced initiation of antimicrobial therapy, limited use of broad-spectrum or empirical antibiotics, and shortened duration of antimicrobial therapy<br>-Duration: 12 months	Blood culture positivity rate; AMR rates (MRSA, ESBL)
**23**	**Olita'a 2019**[49]	Observational (descriptive case series)	Port Moresby, Papua New Guinea	Facility – tertiary referral hospital	N=133; newborns whose mothers had PROM more than 12 hours before delivery, whether prelabour or intrapartum Newborns delivered via normal vaginal	Optimization	-A protocol for minimal or no antibiotics in term babies born after PROM<br>-Duration: 9 months	Neonatal mortality; neonatal sepsis; time when clinical signs of sepsis was evident (within 24 hours, 24 to 48 hours, 48 to 72 hours, 72 hours to 7 days); neonatal sepsis

					delivery, caesarean section or assisted instrumental delivery, with good Apgar scores (>7 at 1 minutes), birth weight of 2 kg or more, and with no clinical signs of sepsis at birth were included. Newborns excluded if born before arrival at hospital (i.e., newborns inborn).			
**24**	**Ren 2022[50]**	Quasi-experimental (uncontrolled before-and-after study)	Guangzhou, China – urban	Facility - level 4 NICU at tertiary women and children's hospital	N=17040; inborn and outborn neonates younger than 37 weeks' gestation or weighing less than 2500 g at birth who were	Education and optimization	-Three QI areas targeted: discontinuation of antibiotic use in ruled-out sepsis within 72 hours; treatment duration for culture-negative pneumonia less than 7 days; and vancomycin or	Neonatal mortality in neonates exposed to antibiotics; LOS in neonates less than 32 weeks' gestation; NEC (Bell stage ≥ II) in neonates less

			admitted to the NICU	meropenem was not used unless the cultured bacteria was only susceptible to them. A training program was also held every month since there were new rotating residents and nurses monthly.<br>-Duration: 1 year and 10 months	than 32 weeks' gestation; suspected or proven infection <5 days; pneumonia < 7 days; purulent meningitis in neonates less than 32 weeks' gestation; neonates with CPS (multidrug-resistant bacteria, *Staphylococcus epidermis*, *K. pneumoniae*, *E. coli,* MRSA); total antibiotic use in DOT; use of meropenem or vancomycin; median DOT (for suspected or proven infection, rule-out sepsis, pneumonia, purulent meningitis, CNS, CPS, EOS, LOS, urinary system

								infection, NEC ≥ stage II); median antibiotic use duration (suspected or proven infection, rule-out sepsis, pneumonia, purulent meningitis, CNS, CPS, EOS, LOS, urinary system infection, NEC ≥ stage II); median length of hospital stay in all patients exposed to antibiotics; median length of hospital stay in infants less than 32 weeks' gestation
**25**	**Sathyan 2022[51]**	Quasi-experimental (uncontrolled before-and-	Kerala, India	Facility – tertiary care teaching hospital (level IIIB unit)	N=83; all inborn and outborn neonates who received NICU care in our unit or any other hospital (for any indication as per	Optimization	-Antibiotic stop policy (Neonates with suspected HAIs and started on antibiotics were classified as one of: (i) 'no Infection' based on cultures negative, CBC	Mortality; NEC stage ≥2; antibiotic overuse days

		after study)			admission policy) for at least 48 hours and started on new antibiotics (for the first time or upgraded from lower antibiotics) for suspected HAI		not suggestive and CRP <10 mg/l at 48 hours of therapy. In these cases, antibiotics stop criteria was at 2 days of therapy, or (ii) ‘probable infection’—any one of: CBC suggesting infection at start of antibiotics (including total leukocyte count <5000 cell/mm3 or a platelet count <1 lakh/ll)/CRP > 10 mg/l at 48 hours of antibiotics/if chest X-ray suggested infection, in which case antibiotics stop criteria was 5 days.)<br>-Duration: approximately 2 years, 5 months	
**26**	**Sewornu 2025**[52]	Quasi-experimental (uncontrolled before-and-	Volta and Oti regions, Ghana	Facility – tertiary, receiving referrals from primary and secondar	N=249 neonates with suspected BSI*<br><br>*Pre-intervention period described in	Regulation, education and optimization	Interventions were implemented to improve the utilisation and quality of blood culture services for diagnosing BSI including:<br>-sensitization	Neonatal sepsis

		after study)	y health facilities for the management of specialised and complicated cases	separate publication by *Boakye-Yiadom 2023*[53]		and training of the treating physicians and nurses such that they develop a high index of suspicion for BSI and use the correct techniques for collecting blood samples<br>-the use of automated incubation systems and improving the availability of laboratory consumables for blood culture and drug susceptibility testing<br>-the laboratory information management system and the EHR were strengthened to better capture key variables such as antimicrobial resistance data and hospital exit outcomes<br>-Duration: 1 year	

**27**	**Sowjanya 2018[54]**	Quasi-experimental (uncontrolled before-and-after study)	Chennai, India – urban	Facility - level 3 NICU at tertiary hospital	N=857; inborn and outborn neonates admitted to the NICU	Optimization	-An antimicrobial justification form devised after discussion with the HIC team based on the overall culture positivity rates, microbiological spectrum of organisms and the antimicrobial sensitivity. Monthly review of the forms was done by the HIC committee.<br>-Duration: 6 months	Neonatal mortality; CPS; new onset severe sepsis, worsening sepsis in spite of empirical therapy; number of newborns on restricted group antibiotics; total duration of restricted group antibiotics; number of newborns started on any antibiotic; number of newborns deescalated from restricted group; mean total duration of first line antibiotics; empirical 1st line antibiotic usage; ampicillin usage; piperacillin-tazobactam usage; amikacin usage; empirical 2nd line antibiotic

								usage; no antibiotic usage
**28**	**Wang 2020**[55]	Quasi-experimental (uncontrolled before-and-after study)†	Beijing, China	Facility-tertiary (level III NICU)	N=9297; all neonates admitted to the NICU – all outborn	Regulation	-Weekly antibiotic surveillance round in the NICU (Each Monday afternoon for 1-1.5 hours, the departmental physician-in-chief led the round, a fixed group of six attending staff neonatologists and one pharmacist attended. All patients who were on antibiotics, including anti-bacterial agents and anti-fungal agents, were reviewed individually. The final decision regarding the use of antibiotics for an individual patient was made on the basis of group discussion.)<br>-Duration: 4 years	Sepsis/suspected sepsis; median length of hospital stay; antibiotic use rate; pediatric conserve antibiotics use rate; DDD; number of patients who received any kinds of antibiotics per 1000 hospital days
**29**	**Xiao-Lu 2016**[56]	Quasi-experimental (uncontrolled	Chongqing, China – urban	Facility – NICU of tertiary children'	N=13310; among patients admitted to the neonatal ward, those who were	Optimization	-Antibiotic stewardship protocol shifting from penicillin and 1st and 2nd generation of cephalosporin to more	Neonatal mortality after discharge for bloodborne infection;

		before-and-after study)	s hospital	diagnosed as neonatal sepsis, purulent cerebral meningitis, and acute hematogenous osteomyelitis (collectively referred to as "bloodborne infections") were included – inborn/outborn not reported		advanced antibiotics, e.g., 3rd and 4th generation of cephalosporin, carbapenem, and glycopeptides<br>-Duration: 1 year	bloodborne infection; nosocomial infection; sepsis (among neonates with bloodborne infection, with positive blood culture); gram-positive bacterial infection; gram-negative bacterial infection; gram-negative fungal infection; drug resistance detected in blood culture from neonatal sepsis (hemolytic staphylococcus: MRS, hemolytic staphylococcus: β-lactamase positive, *Staphylococcus epidermidis*: MRS, *Staphylococcus epidermidis*: β-lactamase positive,

			Klebsiella pneumoniae: ESBL positive, *E. coli*: ESBL positive); percentage of newborns receiving antibiotics; intensity of antibiotic usage (DDD); median length (antibiotic usage, hospitalization of patients with bloodborne infection); bloodborne infection cases with application (penicillin, 1st and 2nd generation cephalosporins, 3rd and 4th generation cephalosporins, carbapenems, glycopeptides)

**30**	**Yin 2020[57]**	Quasi-experimental (uncontrolled before and after study)‡	Shanghai, China – urban	Facility – NICU of tertiary children's hospital	N=13223; inborn and outborn neonates with CRE colonization	Regulation and optimization	-Active CRE surveillance (including colonization screening, culture, and antimicrobial susceptibility testing) and patient placement in contact isolation (4 types: 'single room placement,' meaning one patient in one room; 'same room placement,' meaning patients infected or colonized with the same CRE were placed together in the same room when single-patient rooms were in short supply; 'same area placement,' meaning patients with the same CRE were placed as a cohort in the same area of a big ward, with a partition barrier; or 'no cohort placement,' meaning patients with or without CRE or with	CRE nosocomial infection incidence in neonatal wards; CRE nosocomial infection incidence in the NICU

							different CRE were in the same room, curtains between each bed, without any cohort placement.)<br>-Duration: 1 year	
**31**	**Zain 2021**[58]	RCT	Rawalpindi, Pakistan – urban	Facility – tertiary hospital	N=120; asymptomatic term neonates (37+ weeks) of either gender with PROM>18 hours – inborn/outborn not reported	Optimization	-Prophylactic antibiotic treatment (group 1) versus selective antibiotic treatment (group 2) in term newborns born after PROM>18 hours in terms of neonatal sepsis and resistance of neonatal flora<br>-Duration: not reported	Neonatal sepsis; resistant neonatal flora

Abbreviations: *A. baumanni, Acinetobacter baumanni*; AMS, antimicrobial stewardship; ASP, antimicrobial stewardship program; AST, antimicrobial susceptibility testing; AUR, antibiotic usage rate; BSI, bloodstream infections; CAUTI, catheter-associated urinary tract infection; CBC, complete blood count; CLABSI, central line-associated bloodstream infection; CNS, culture-negative sepsis; CoNS, coagulase-negative *Staphylococci*; CPS, culture-positive sepsis; CRE, carbapenem-resistant *Enterobacterales*; CRP, C-reactive protein; DDD, defined daily dose; DOT, days of therapy; *E. coli, Escherichia coli*; EHR, electronic health record; EOS, early-onset sepsis; ESBL, extended-spectrum β-lactamase; GA, gestational age; HAI, hospital-acquired/healthcare-associated infection; HH, hand hygiene; HIC, hospital infection control; ICU, intensive care unit; IV, intravenous; *K. pneumonia, Klebsiella pneumoniae*; kg, kilogram; LOS, late-onset sepsis; LOT, length of therapy; MDROs, multidrug-resistant organisms; MRS, methicillin-resistant *Staphylococcus*; MRSA, methicillin-resistant *Staphylococcus aureus*; NEC, necrotizing enterocolitis; NICU, neonatal intensive care unit; NS-ASP, neonatal-specific antimicrobial stewardship program; *P. aeruginosa, Pseudomonas aeruginosa*; PDSA, Plan-Do-Study-Act; PROM, prolonged rupture of membranes; QI, quality improvement; RCT, randomized controlled trial; *S. aureus, Staphylococcus aureus*; SMAP, Smart Use of Antibiotics Program; SSI, surgical site infection; VAP, ventilator-associated pneumonia; VLBW, very low birth weight; VRE, vancomycin-resistant *Enterococci*; WHO, World Health Organization

*Chu 2023 reported their study design as "a single-centre, retrospective cohort study;" however, we have re-classified it as an uncontrolled before-and-after design based on a careful assessment of the methodological features of the study.

†Wang 2020 reported their study design as a "retrospective observational study;" however, we have re-classified it as an uncontrolled before-and-after design based on a careful assessment of the methodological features of the study.

‡Yin 2020 reported their study design as a "single-centre, cross-sectional retrospective study;" however, we have re-classified it as an uncontrolled before-and-after design based on a careful assessment of the methodological features of the study.

Appendix H: Geographical distribution of studies implementing strategies to reduce AMR

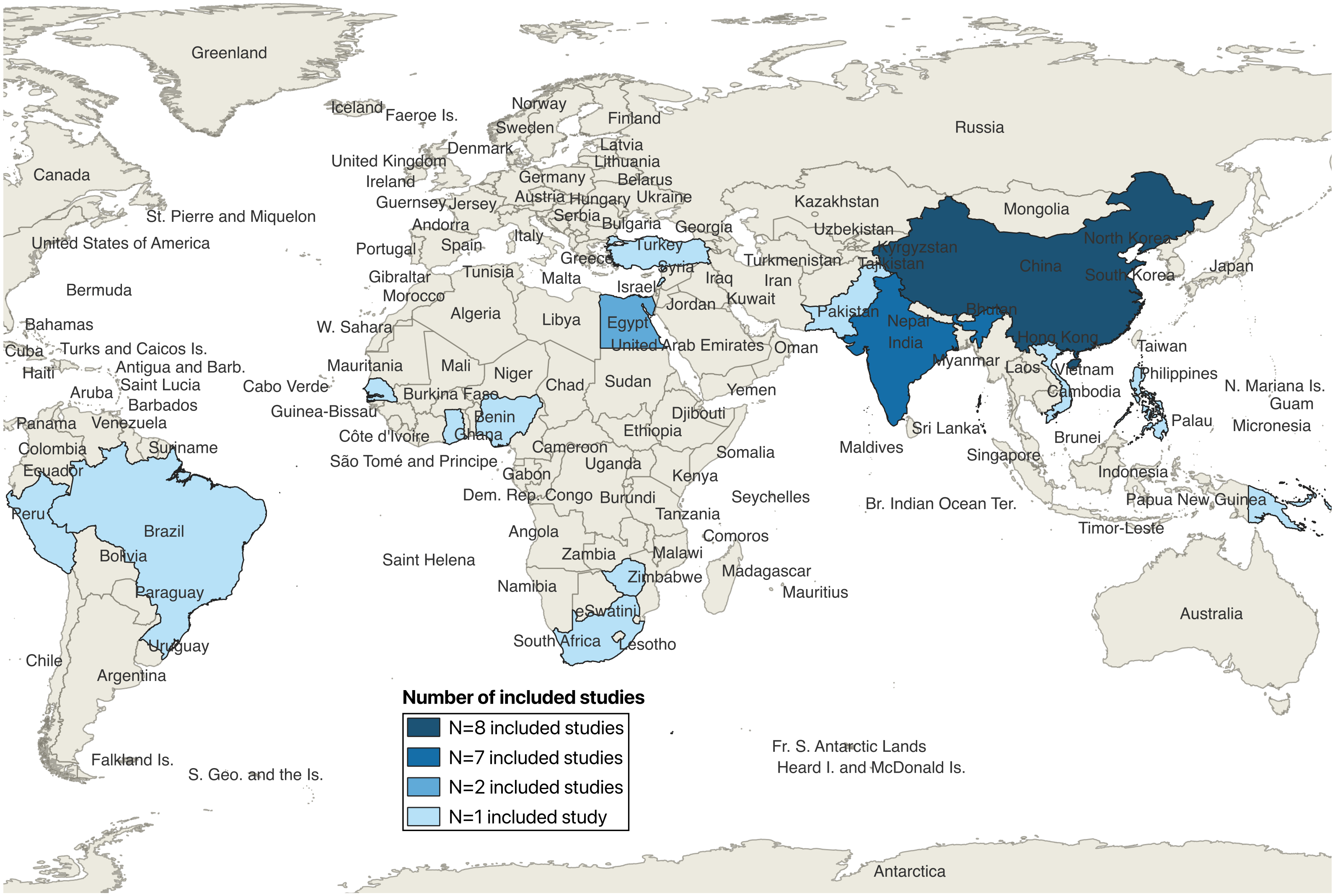

Figure 1: **Geographical distribution of included studies which implemented strategies to reduce antimicrobial resistance in newborn care**

Appendix I: Key characteristics of included studies tabulated

Table 13: **Counts of key characteristics of included studies**

Characteristics	No. (%) of studies (n=31)
**Study design:**	
**Randomized controlled trials**	1 (3.23)
**Quasi-experimental studies**	28 (90.32)
**Observational studies**	2 (6.45)
**WHO region:**	
**Africa**	6 (19.35)
**Americas**	2 (6.45)
**Eastern Mediterranean**	4 (12.90)
**Europe**	1 (3.23)
**South-East Asia**	7 (22.58)
**Western Pacific**	11 (35.48)
**World Bank country income level:**	
**Upper-middle income**	12 (38.71)
**Lower-middle income**	18 (58.06)
**Low income**	1 (3.23)
**Level of care:**	
**Facility**	31 (100)
**Tertiary or higher only**	29 (93.55)
**Secondary only**	1 (3.23)
**Primary only**	0
**Mixed primary, secondary, and tertiary or higher**	1 (3.23)
**Community**	0
**Study population:**	
**Inborn only**	5 (16.13)
**Outborn only**	5 (16.13)

**Mixed inborn and outborn**	11 (35.48)
**Not reported**	10 (32.26)
**Intervention categories:**	
**Single-component**	10 (32.36)
**Regulation**	3 (30)
**Education**	0
**Optimization**	7 (70)
**Multi-component**	21 (67.74)
**Regulation and education**	3 (14.29)
**Regulation and optimization**	3 (14.29)
**Education and optimization**	1 (4.76)
**Regulation, education, and optimization**	14 (66.67)

Abbreviations: WHO, World Health Organization

Appendix J: Risk of bias assessment

Table 14: **Risk of bias for randomized trials using the RoB-2 assessment tool (n=1 study)**

No.	Study	Randomization process	Deviations from the intended interventions	Missing outcome data	Measurement of the outcome	Selection of the reported result	Overall*
1	**Zain 2021[58]**	Some concerns	Some concerns	Low risk	Some concerns	Some concerns	Some concerns

*Overall risk of bias judgement: 'Low risk' indicates the study is judged to be at low risk of bias for all domains for this result. 'Some concerns' indicate the study is judged to raise some concerns in at least one domain for this result, but not to be at high risk of bias for any domain. 'High risk' indicates the study is judged to be at high risk of bias in at least one domain for this result or the study is judged to have some concerns for multiple domains in a way that substantially lowers confidence in the result.[59]

Table 15: **Risk of bias for non-randomized, quasi-experimental studies using the ROBINS-I assessment tool (n=28 studies)**

No.	Study	Bias due to confounding	Bias in selection of participants into the study	Bias in classification of interventions	Bias due to deviations from intended interventions	Bias due to missing data	Bias in measurement of outcomes	Bias in selection of the reported results	Overall bias*
1	**Agarwal 2021[26]**	Moderate risk	Low risk	Low risk	Low risk	Low risk	Low risk	Low risk	Moderate risk
2	**Alp 2015[28]**	Moderate risk	Moderate risk	Moderate risk	Low risk	Low risk	Low risk	Low risk	Moderate risk
3	**Bassiouny 2020[29]**	Moderate risk	Low risk	Moderate risk	Low risk	Low risk	Low risk	Low risk	Moderate risk
4	**Chimhini 2020[30]**	Moderate risk	Moderate risk	Moderate risk	Moderate risk	Low risk	Moderate risk	Low risk	Moderate risk
5	**Chu 2023[31]**	Moderate risk	Moderate risk	Moderate risk	Low risk	Low risk	Low risk	Low risk	Moderate risk
6	**Dramowski 2024[32]**	Moderate risk	Moderate risk	Low risk	Low risk	Low risk	Low risk	Low risk	Moderate risk
7	**El-Baky 2020[33]**	Moderate risk	Moderate risk	Low risk	Moderate risk	Low risk	Low risk	Low risk	Moderate risk
8	**Farias-Filho 2024[34]**	Moderate risk	Serious risk	Moderate risk	Low risk	Low risk	Serious risk	Low risk	Serious risk
9	**Feng 2022[35]**	Moderate risk	Moderate risk	Low risk	Low risk	Low risk	Low risk	Low risk	Moderate risk

**10**	**Garpvall 2021**[36]	Serious risk	Moderate risk	Low risk	Serious risk	Moderate risk	Serious risk	Low risk	Serious risk
**11**	**Gill 2008**[37]	Moderate risk	Low risk	Moderate risk	Low risk	Low risk	Low risk	Low risk	Moderate risk
**12**	**Graus 2022**[38]	Moderate risk	Moderate risk	Low risk	Low risk	Low risk	Low risk	Low risk	Moderate risk
**13**	**Huang 2019**[39]	Moderate risk	Moderate risk	Low risk	Low risk	Low risk	Low risk	Low risk	Moderate risk
**14**	**Jain 2021**[40]	Moderate risk	Serious risk	Low risk	Serious risk	Low risk	Low risk	Low risk	Serious risk
**15**	**Jinka 2017**[41]	Moderate risk	Moderate risk	Low risk	Low risk	Low risk	Low risk	Low risk	Moderate risk
**16**	**Kommalur 2021**[42]	Moderate risk	Low risk	Low risk	Moderate risk	Low risk	Low risk	Low risk	Moderate risk
**17**	**Konda 2021**[43]	Moderate risk	Serious risk	Low risk	Serious risk	Low risk	Low risk	Low risk	Serious risk
**18**	**Landre-Peigne 2021**[44]	Moderate risk	Moderate risk	Low risk	Low risk	Low risk	Low risk	Low risk	Moderate risk
**19**	**Lu 2019**[45]	Moderate risk	Moderate risk	Low risk	Low risk	Low risk	Low risk	Low risk	Moderate risk
**20**	**Maalouf 2023**[46]	Moderate risk	Moderate risk	Low risk	Low risk	Low risk	Low risk	Low risk	Moderate risk
**21**	**Nygren 2025**[47]	Serious risk	Serious risk	Moderate risk	Moderate risk	Low risk	Moderate risk	Low risk	Serious risk
**22**	**Ren 2022**[50]	Moderate risk	Low risk	Low risk	Low risk	Low risk	Low risk	Low risk	Moderate risk
**23**	**Sathyan 2022**[51]	Moderate risk	Moderate risk	Moderate risk	Low risk	Low risk	Moderate risk	Low risk	Moderate risk
**24**	**Sewornu 2025**[52]	Serious risk	Moderate risk	Moderate risk	Moderate risk	Serious risk	Low risk	Low risk	Serious risk
**25**	**Sowjanya 2018**[54]	Moderate risk	Moderate risk	Low risk	Low risk	Low risk	Low risk	Low risk	Moderate risk
**26**	**Wang 2020**[55]	Moderate risk	Moderate risk	Moderate risk	Low risk	Low risk	Low risk	Low risk	Moderate risk
**27**	**Xiao-Lu 2016**[56]	Moderate risk	Moderate risk	Moderate risk	Low risk	Low risk	Low risk	Low risk	Moderate risk

**28**	**Yin 2020[57]**	Moderate risk	Moderate risk	Moderate risk	Low risk	Low risk	Low risk	Low risk	Moderate risk

*Overall risk of bias judgement: 'Low risk' indicates the study is judged to be at low risk of bias for all domains. This means the study is comparable to a well-performed randomized trial. 'Moderate risk' indicates the study is judged to be at low or moderate risk of bias for all domains. This means the study provides sound evidence for a non-randomized study but cannot be considered comparable to a well-performed randomized trial. 'Serious risk' indicates the study is judged to be at serious risk of bias in at least one domain, but not at critical risk of bias in any domain. This means the study has some important problems. 'Critical risk' indicates the study is judged to be at critical risk of bias in at least one domain. This means the study is too problematic to provide any useful evidence and should not be included in any synthesis. 'No information' indicates there is no clear indication that the study is at serious or critical risk of bias and there is a lack of information in one or more key domains of bias (a judgement is required for this). This means there was no information on which to base a judgement about risk of bias.[60]

Table 16: **Risk of bias for observational studies using the NIH assessment tool (n=2 studies)**

No.	Study	Q1	Q2	Q3	Q4	Q5	Q6	Q7	Q8	Q9	Q10	Q11	Q12	Q13	Q14	Overall*
1	**Akintan 2024[27]***	Yes	Yes	Yes	Yes	No	No	Yes	N/A	Yes	Yes	Yes	No	CD	No	Fair
2	**Olita'a 2019[49]†**	Yes	Yes	No	CD	Yes	CD	Yes	Yes	Yes						Fair

**NIH assessment tool criteria for observational cohort and cross-sectional studies***	
**Q1**	Was the research question or objective in this paper clearly stated?
**Q2**	Was the study population clearly specified and defined?
**Q3**	Was the participation rate of eligible persons at least 50%?
**Q4**	Were all the subjects selected or recruited from the same or similar populations (including the same time period)? Were inclusion and exclusion criteria for being in the study prespecified and applied uniformly to all participants?
**Q5**	Was a sample size justification, power description, or variance and effect estimates provided?
**Q6**	For the analyses in this paper, were the exposure(s) of interest measured prior to the outcome(s) being measured?
**Q7**	Was the timeframe sufficient so that one could reasonably expect to see an association between exposure and outcome if it existed?
**Q8**	For exposures that can vary in amount or level, did the study examine different levels of the exposure as related to the outcome (e.g., categories of exposure, or exposure measured as continuous variable)?
**Q9**	Were the exposure measures (independent variables) clearly defined, valid, reliable, and implemented consistently across all study participants?
**Q10**	Was the exposure(s) assessed more than once over time?
**Q11**	Were the outcomes measures (dependent variables) clearly defined, valid, reliable, and implemented consistently across all study participants?
**Q12**	Were the outcome assessors blinded to the exposure status of participants?
**Q13**	Was loss to follow-up after baseline 20% or less?
**Q14**	Were key potential confounding variables measured and adjusted statistically for their impact on the relationship between exposure(s) and outcome(s)?

**NIH assessment tool criteria for case series studies†**	
**Q1**	Was the study question or objective clearly stated?
**Q2**	Was the study population clearly and fully described, including a case definition?
**Q3**	Were the cases consecutive?
**Q4**	Were the subjects comparable?
**Q5**	Was the intervention clearly described?
**Q6**	Were the outcome measures clearly defined, valid, reliable, and implemented consistently across all study participants?
**Q7**	Was the length of follow-up adequate?
**Q8**	Were the statistical methods well-described?
**Q9**	Were the results well-described?

Abbreviations: CD, cannot determine; N/A, not applicable

*Overall rating may be considered good, fair, or poor. In general, a "good" study has the least risk of bias and results are considered to be valid, a "fair" study is susceptible to some bias deemed not sufficient to invalidate its results, and a "poor" rating indicates significant risk of bias.[61]

Appendix K: GRADE certainty of evidence for neonatal mortality outcomes

The certainty of evidence was rated (very low, low, moderate, high) for each outcome in accordance with the GRADE framework[62]. Evidence was downgraded based on five factors: risk of bias, inconsistency, indirectness, imprecision, and publication bias. The criteria and reasons for downgrading were provided in explanatory footnotes in the GRADE tables below.

Table 17: **GRADE evidence profile for optimization interventions compared to usual care for reducing all-cause neonatal mortality**
Question: Optimization interventions compared to usual care for reducing all-cause neonatal mortality
Setting: Low- and middle-income countries

Certainty assessment							№ of patients		Effect		Certainty	Importance
№ of studies	Study design	Risk of bias	Inconsistency	Indirectness	Imprecision	Other considerations	Optimization interventions	Usual care	Relative (95% CI)	Absolute (95% CI)		
**All-cause neonatal mortality**												
3	non-randomized studies[a]	serious[b]	not serious[c]	not serious	serious[d]	none	18/941 (1.9%)	13/637 (2.0%)	**RR 1.12** (0.55 to 2.28)	**2 more per 1,000** (from 9 fewer to 26 more)	⨁◯◯◯ Very low	CRITICAL

Abbreviations: CI: confidence interval; **RR:** risk ratio

Explanations

[a]*Sathyan 2022*, *Sowjanya 2018*, and *Xiao-Lu 2016* have uncontrolled before-and-after quasi-experimental study designs.

[b]Downgraded by one level as crucial limitations exist for one or more criteria. All studies were judged to have a moderate risk of bias using the ROBINS-I tool. *Sathyan 2022* was judged to have a moderate risk of bias for concerns over confounding, selection, and outcome measurement biases, given extraneous changes which could have influenced the outcome, and differing methods of outcome assessment before and after the intervention (i.e., retrospective data collection in the pre-intervention period and prospective data collection in the post-intervention period). A conflict-of-interest statement was also not reported. *Sowjanya 2018* was judged to have a moderate risk of bias for concerns over the lack of reporting on various study characteristics including the study eligibility criteria and characteristics of included newborns. The study also did not provide statements on funding sources or conflicts of interest. *Xiao-Lu 2016* was judged to have a moderate risk of bias due to confounding bias, the intervention not being clearly defined, and the choice of outcome measure (i.e., defined daily dose) not being

explained given defined daily dose (DDD) is a metric normally used to measure antibiotic consumption in an adult population, not an infant population. The study also did not provide a conflict-of-interest statement.
[c]Inconsistency not serious (I^2=0%).
[d]Downgraded by one level as 95% CI captures the null value.

Table 18: **GRADE evidence profile for regulation and optimization interventions compared to usual care for reducing neonatal mortality due to monomial bloodstream infections**
Question: Regulation and optimization interventions compared to usual care for reducing neonatal mortality due to nosocomial bloodstream infections
Setting: Low- and middle-income countries

Certainty assessment							№ of patients		Effect		Certainty	Importance
№ of studies	Study design	Risk of bias	Inconsistency	Indirectness	Imprecision	Other considerations	regulation and restriction interventions	usual care	Relative (95% CI)	Absolute (95% CI)		
Neonatal mortality due to nosocomial bloodstream infection												
2	non-randomized studies	serious[a]	not serious[b]	not serious	not serious[c]	none	14/765 (1.8%)	17/587 (2.9%)	RR 0.62 (0.31 to 1.25)	11 fewer per 1,000 (from 20 fewer to 7 more)	⨁◯◯◯ Very low	CRITICAL

Abbreviations: CI: confidence interval; **RR:** risk ratio

Explanations
[a]Downgraded by one level as crucial limitations exist for one or more criteria. *El-Baky 2020* was judged to have a moderate risk of bias using the ROBINS-I tool due to potential confounding bias, lack of reporting on inclusion criteria and concerns about intervention compliance. *Landre-Peigne 2011* was judged to have a moderate risk of bias using the ROBINS-I tool due to potential confounding bias and lack of clear reporting in some instances.
[b]Inconsistency not serious (I^2=0%).
[c]95% CI captures the null value.

Table 19: **GRADE evidence profile for regulation, education, and optimization interventions compared to usual care for reducing all-cause neonatal mortality**
Question: Regulation, education, and optimization interventions compared to usual care for reducing all-cause neonatal mortality
Setting: Low- and middle-income countries

Certainty assessment							№ of patients		Effect		Certainty	Importance
№ of studies	Study design	Risk of bias	Inconsistency	Indirectness	Imprecision	Other considerations	Regulation, education, and optimization interventions	Usual care	Relative (95% CI)	Absolute (95% CI)		
**All-cause neonatal mortality**												
8	non-randomized studies[a]	serious[b]	serious[c]	not serious	not serious[d]	none	1136/12849 (8.8%)	1470/16079 (9.1%)	**RR 0.73** (0.57 to 0.93)	**25 fewer per 1,000** (from 39 fewer to 6 fewer)	⨁◯◯◯ Very low	CRITICAL

Abbreviations: CI: confidence interval; **RR:** risk ratio

Explanations

[a]All studies have quasi-experimental study designs.

[b]Downgraded by one level as crucial limitations exist for one or more criteria. 7/8 studies judged as having a moderate risk of bias. 1/8 studies judged as having a serious risk of bias due to potential confounding bias, reported non-adherence to the intervention, and lack of reporting on several study characteristics including characteristics of included newborns; however, sensitivity analysis omitting the serious risk of bias study indicated the findings were robust.

[c]Downgraded by one level for high between-study heterogeneity (I^2=85%), though the direction of effect consistently favors post-intervention.

[d]95% CI does not capture the null value.

Appendix L: Forest plots

Regulation

Outcome: Sepsis/suspected sepsis (by study design)

Study or Subgroup	After Intervention Events	After Intervention Total	Before Intervention Events	Before Intervention Total	Weight	Risk Ratio IV, Random, 95% CI
**1.2.1 Observational**						
Akintan 2024 (1)	12	19	5	19	40.9%	2.40 [1.05, 5.49]
**Subtotal (95% CI)**		**19**		**19**	**40.9%**	**2.40 [1.05, 5.49]**
Total events	12		5			
Heterogeneity: Not applicable						
Test for overall effect: Z = 2.07 (P = 0.04)						
**1.2.2 Quasi-experimental**						
Wang 2020 (2)	356	4488	417	4743	59.1%	0.90 [0.79, 1.03]
**Subtotal (95% CI)**		**4488**		**4743**	**59.1%**	**0.90 [0.79, 1.03]**
Total events	356		417			
Heterogeneity: Not applicable						
Test for overall effect: Z = 1.49 (P = 0.14)						
**Total (95% CI)**		**4507**		**4762**	**100.0%**	**1.35 [0.52, 3.46]**
Total events	368		422			

Heterogeneity: $Tau^2 = 0.39$; $Chi^2 = 5.24$, df = 1 (P = 0.02); $I^2 = 81\%$
Test for overall effect: Z = 0.62 (P = 0.54)
Test for subgroup differences: $Chi^2 = 5.24$, df = 1 (P = 0.02), $I^2 = 80.9\%$

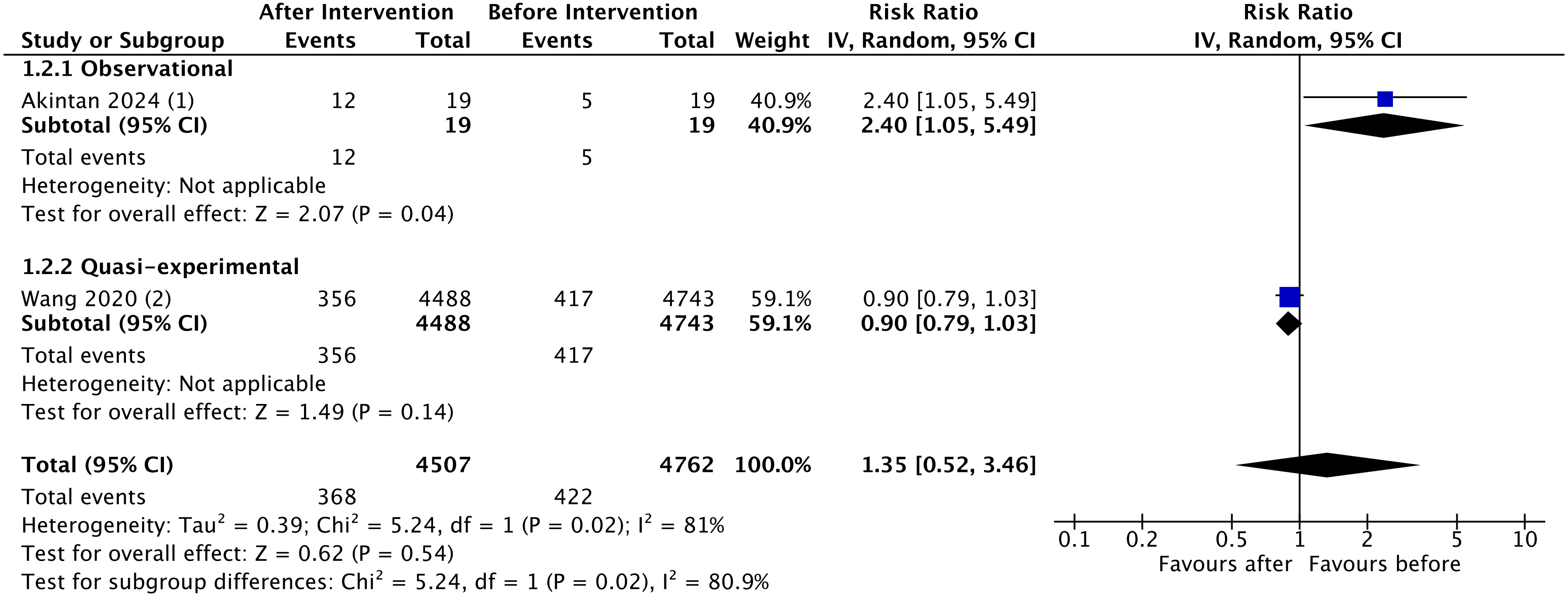

<u>Footnotes</u>
(1) Akintan 2024 reported sepsis among inpatient neonates
(2) Wang 2020 reported sepsis/suspected sepsis among inpatient neonates

Outcome: Number of neonates receiving at least one antimicrobial (by study design)

	After Intervention		Before Intervention			Risk Ratio
**Study or Subgroup**	**Events**	**Total**	**Events**	**Total**	**Weight**	**IV, Random, 95% CI**
**1.4.1 Observational**						
Akintan 2024	17	19	15	19	42.5%	1.13 [0.86, 1.50]
**Subtotal (95% CI)**		**19**		**19**	**42.5%**	**1.13 [0.86, 1.50]**
Total events	17		15			
Heterogeneity: Not applicable						
Test for overall effect: Z = 0.88 (P = 0.38)						
**1.4.2 Quasi-experimental**						
Wang 2020	3328	4488	4478	4743	57.5%	0.79 [0.77, 0.80]
**Subtotal (95% CI)**		**4488**		**4743**	**57.5%**	**0.79 [0.77, 0.80]**
Total events	3328		4478			
Heterogeneity: Not applicable						
Test for overall effect: Z = 25.44 (P < 0.00001)						
**Total (95% CI)**		**4507**		**4762**	**100.0%**	**0.92 [0.64, 1.31]**
Total events	3345		4493			
Heterogeneity: $Tau^2 = 0.06$; $Chi^2 = 6.62$, df = 1 (P = 0.01); $I^2 = 85\%$						
Test for overall effect: Z = 0.47 (P = 0.64)						
Test for subgroup differences: $Chi^2 = 6.62$, df = 1 (P = 0.01), $I^2 = 84.9\%$						

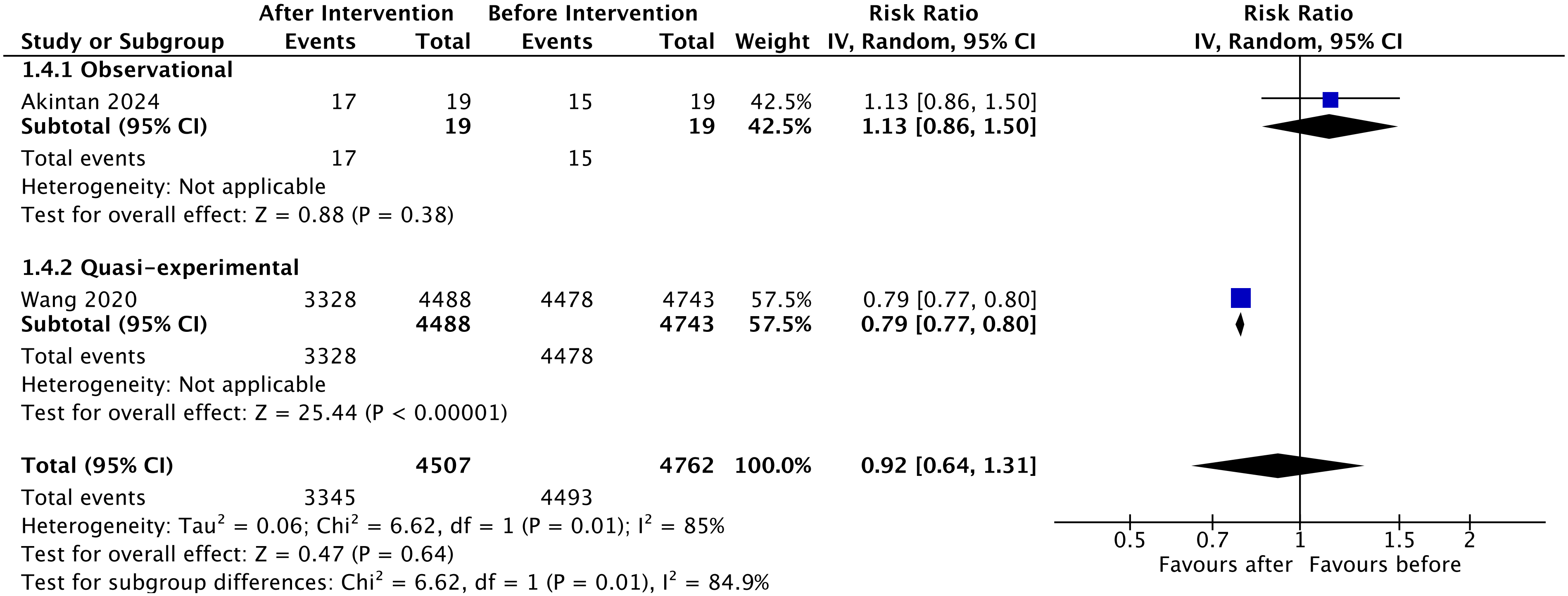

Optimization

Outcome: All-cause neonatal mortality

Study or Subgroup	After Intervention Events	After Intervention Total	Before Intervention Events	Before Intervention Total	Weight	Risk Ratio IV, Random, 95% CI
Sathyan 2022	1	43	1	40	6.8%	0.93 [0.06, 14.38]
Sowjanya 2018	12	428	10	429	74.1%	1.20 [0.53, 2.75]
Xiao-Lu 2016	5	470	2	168	19.1%	0.89 [0.18, 4.56]
**Total (95% CI)**		**941**		**637**	**100.0%**	**1.12 [0.55, 2.28]**
Total events	18		13			

Heterogeneity: Tau^2 = 0.00; Chi^2 = 0.12, df = 2 (P = 0.94); I^2 = 0%
Test for overall effect: Z = 0.30 (P = 0.76)

Risk Ratio IV, Random, 95% CI

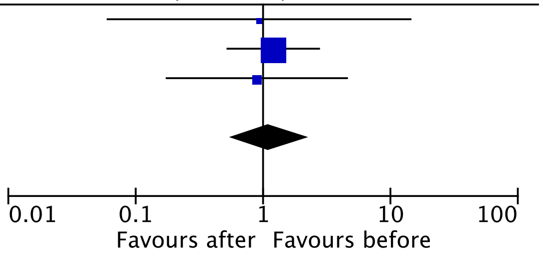

Outcome: Culture-positive sepsis

Study or Subgroup	After Intervention Events	After Intervention Total	Before Intervention Events	Before Intervention Total	Weight	Risk Ratio IV, Random, 95% CI
Sowjanya 2018	14	428	18	429	8.8%	0.78 [0.39, 1.55]
Xiao-Lu 2016	144	470	77	168	91.2%	0.67 [0.54, 0.83]
**Total (95% CI)**		**898**		**597**	**100.0%**	**0.68 [0.55, 0.83]**
Total events	158		95			

Heterogeneity: Tau^2 = 0.00; Chi^2 = 0.18, df = 1 (P = 0.67); I^2 = 0%
Test for overall effect: Z = 3.74 (P = 0.0002)

Risk Ratio IV, Random, 95% CI

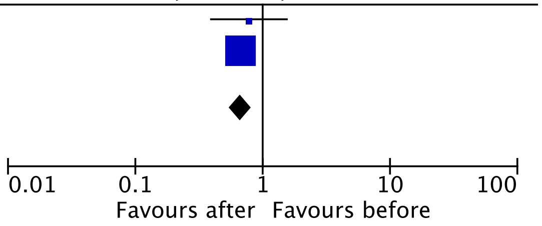

Outcome: Number of neonates on antibiotics

Study or Subgroup	After Intervention Events	After Intervention Total	Before Intervention Events	Before Intervention Total	Weight	Risk Ratio IV, Random, 95% CI
Jinka 2017	584	1276	681	1176	31.9%	0.79 [0.73, 0.85]
Sowjanya 2018	370	428	385	429	34.9%	0.96 [0.92, 1.01]
Xiao-Lu 2016	372	470	154	168	33.3%	0.86 [0.81, 0.92]
**Total (95% CI)**		**2174**		**1773**	**100.0%**	**0.87 [0.78, 0.98]**
Total events	1326		1220			

Heterogeneity: Tau^2 = 0.01; Chi^2 = 19.71, df = 2 (P < 0.0001); I^2 = 90%
Test for overall effect: Z = 2.35 (P = 0.02)

Risk Ratio IV, Random, 95% CI

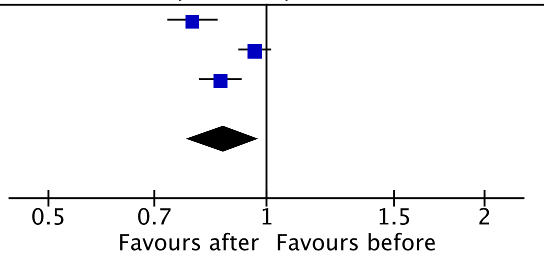

Outcome: AWaRe[63] antibiotic usage

Study or Subgroup	After Intervention Events	After Intervention Total	Before Intervention Events	Before Intervention Total	Weight	Risk Ratio IV, Random, 95% CI
**1.3.1 Access group**						
Jinka 2017 - Amikacin	116	584	301	681	7.1%	0.45 [0.37, 0.54]
Jinka 2017 - Ampicillin/Gentamicin	491	584	449	681	7.3%	1.28 [1.20, 1.36]
Sowjanya 2018 - Amikacin	160	428	190	429	7.2%	0.84 [0.72, 0.99]
Sowjanya 2018 -Ampicillin	40	428	45	429	6.2%	0.89 [0.59, 1.33]
Xiao-Lu 2016 - Penicillin	346	470	156	168	7.3%	0.79 [0.74, 0.85]
**Subtotal (95% CI)**		**2494**		**2388**	**35.2%**	**0.81 [0.58, 1.13]**
Total events	1153		1141			
Heterogeneity: $Tau^2 = 0.13; Chi^2 = 172.73, df = 4 (P < 0.00001); I^2 = 98\%$						
Test for overall effect: Z = 1.25 (P = 0.21)						
**1.3.2 Watch group**						
Jinka 2017 - 3rd Gen. Cephalosporins	39	584	281	681	6.6%	0.16 [0.12, 0.22]
Jinka 2017 - Ciprofloxacin	45	584	5	681	3.7%	10.49 [4.19, 26.26]
Jinka 2017 - Meropenem	58	584	58	681	6.5%	1.17 [0.82, 1.65]
Jinka 2017 - Piperacillin-Tazobactam	81	584	109	681	6.8%	0.87 [0.66, 1.13]
Sowjanya 2018 - Piperacillin-Tazobactam	155	428	175	429	7.1%	0.89 [0.75, 1.05]
Xiao-Lu 2016 - 3rd and 4th Gen. Cephalosporins	187	470	90	168	7.1%	0.74 [0.62, 0.89]
Xiao-Lu 2016 - Carbapenems	168	470	27	168	6.4%	2.22 [1.54, 3.21]
Xiao-Lu 2016 - Glycopeptides	72	470	32	168	6.3%	0.80 [0.55, 1.17]
**Subtotal (95% CI)**		**4174**		**3657**	**50.6%**	**1.00 [0.61, 1.63]**
Total events	805		777			
Heterogeneity: $Tau^2 = 0.45; Chi^2 = 165.33, df = 7 (P < 0.00001); I^2 = 96\%$						
Test for overall effect: Z = 0.00 (P = 1.00)						
**1.3.3 Mix of Access and Watch group**						
Sowjanya 2018 - Empirical 1st Line (1)	175	428	195	429	7.2%	0.90 [0.77, 1.05]
Xiao-Lu 2016 - 1st and 2nd Gen. Cephalosporins	100	470	126	168	7.1%	0.28 [0.23, 0.34]
**Subtotal (95% CI)**		**898**		**597**	**14.3%**	**0.51 [0.16, 1.57]**
Total events	275		321			
Heterogeneity: $Tau^2 = 0.66; Chi^2 = 83.07, df = 1 (P < 0.00001); I^2 = 99\%$						
Test for overall effect: Z = 1.18 (P = 0.24)						
**Total (95% CI)**		**7566**		**6642**	**100.0%**	**0.82 [0.64, 1.06]**
Total events	2233		2239			
Heterogeneity: $Tau^2 = 0.22; Chi^2 = 486.74, df = 14 (P < 0.00001); I^2 = 97\%$						
Test for overall effect: Z = 1.54 (P = 0.12)						
Test for subgroup differences: $Chi^2 = 1.32, df = 2 (P = 0.52), I^2 = 0\%$						

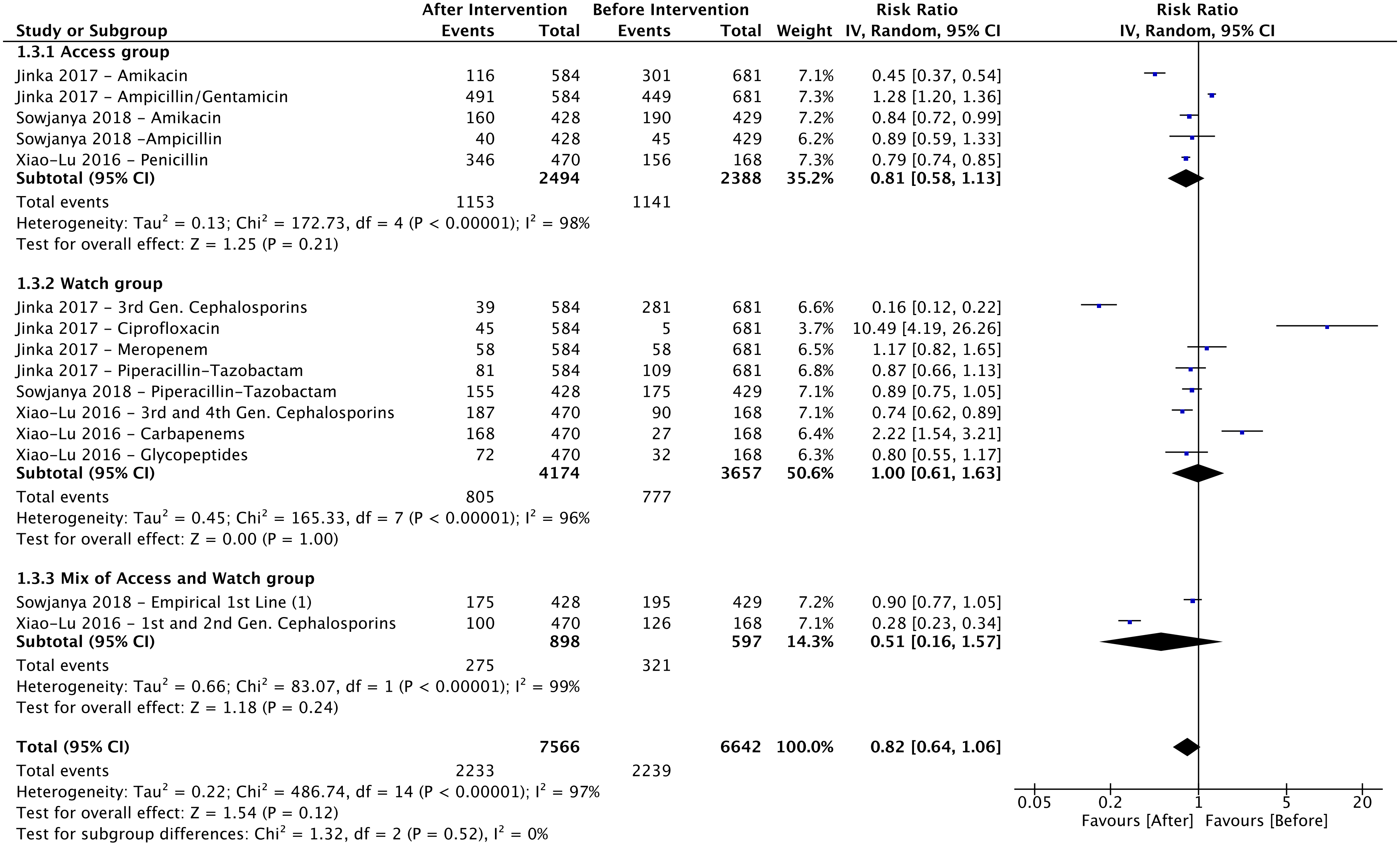

Footnotes

(1) Amikacin, Ampicillin, and Piperacillin-Tazobactam

Regulation and Optimization

Outcome: Neonatal mortality due to nosocomial bloodstream infection (by level of care)

Study or Subgroup	After Intervention Events	After Intervention Total	Before Intervention Events	Before Intervention Total	Weight	Risk Ratio IV, Random, 95% CI
**1.37.1 Secondary**						
Landre–Peigne 2021	1	148	1	125	6.4%	0.84 [0.05, 13.37]
**Subtotal (95% CI)**		**148**		**125**	**6.4%**	**0.84 [0.05, 13.37]**
Total events	1		1			
Heterogeneity: Not applicable						
Test for overall effect: Z = 0.12 (P = 0.90)						
**1.37.2 Tertiary or higher**						
El–Baky 2020 (1)	13	617	16	462	93.6%	0.61 [0.30, 1.25]
**Subtotal (95% CI)**		**617**		**462**	**93.6%**	**0.61 [0.30, 1.25]**
Total events	13		16			
Heterogeneity: Not applicable						
Test for overall effect: Z = 1.35 (P = 0.18)						
**Total (95% CI)**		**765**		**587**	**100.0%**	**0.62 [0.31, 1.25]**
Total events	14		17			
Heterogeneity: $Tau^2 = 0.00$; $Chi^2 = 0.05$, df = 1 (P = 0.82); $I^2 = 0\%$						
Test for overall effect: Z = 1.34 (P = 0.18)						
Test for subgroup differences: $Chi^2 = 0.05$, df = 1 (P = 0.82), $I^2 = 0\%$						

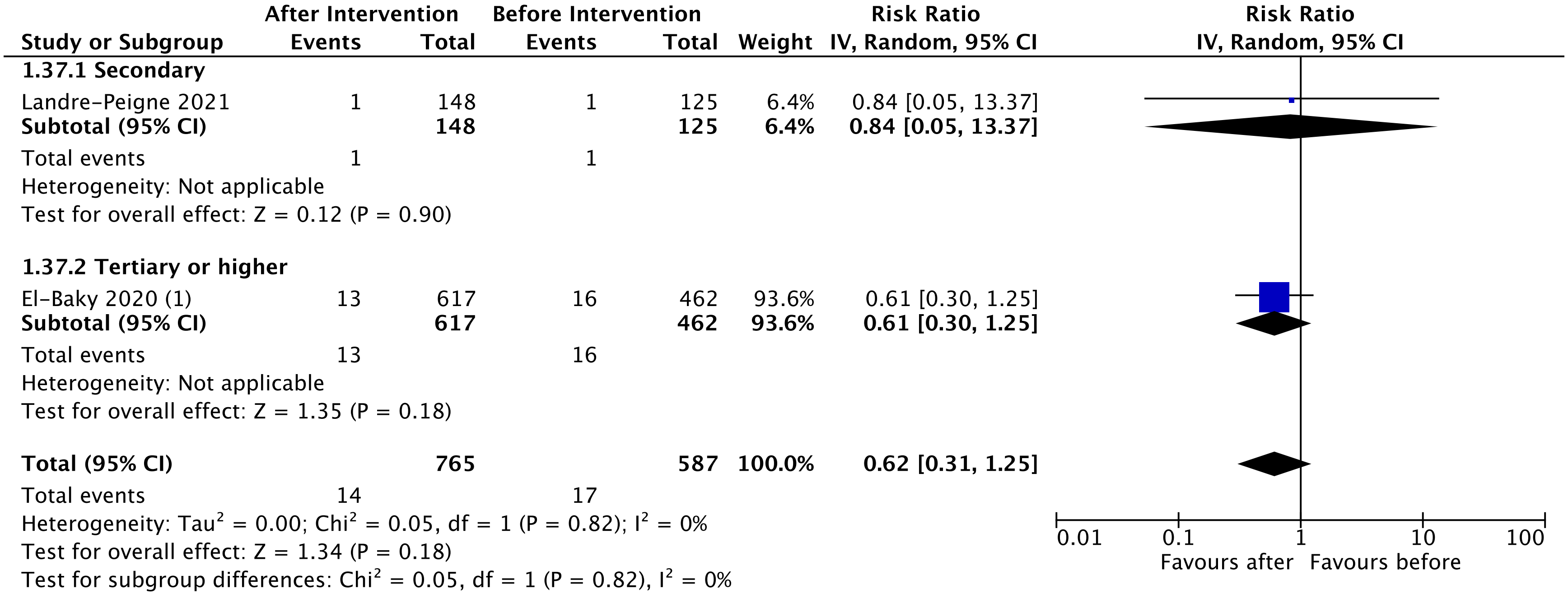

Footnotes
(1) Data updated through direct correspondance with study authors

Regulation, Education, and Optimization

Outcome: All-cause neonatal mortality

Study or Subgroup	After Intervention Events	After Intervention Total	Before Intervention Events	Before Intervention Total	Weight	Risk Ratio IV, Random, 95% CI
Agarwal 2021	56	864	20	290	10.8%	0.94 [0.57, 1.54]
Bassiouny 2020	31	150	20	60	11.2%	0.62 [0.39, 1.00]
Chu 2023	2	90	3	96	1.7%	0.71 [0.12, 4.16]
Feng 2022	0	2901	3	4804	0.7%	0.24 [0.01, 4.58]
Gill 2009 (1)	144	1000	290	1000	17.6%	0.50 [0.41, 0.59]
Gill 2009 (2)	481	1000	598	1000	19.1%	0.80 [0.74, 0.87]
Jain 2021 (3)	16	1000	25	1000	8.6%	0.64 [0.34, 1.19]
Kommalur 2021	18	58	38	75	11.8%	0.61 [0.39, 0.95]
Lu 2019	388	5786	473	7754	18.5%	1.10 [0.97, 1.25]
**Total (95% CI)**		**12849**		**16079**	**100.0%**	**0.73 [0.57, 0.93]**
Total events	1136		1470			

Heterogeneity: Tau^2 = 0.08; Chi^2 = 54.12, df = 8 (P < 0.00001); I^2 = 85%

Test for overall effect: Z = 2.57 (P = 0.01)

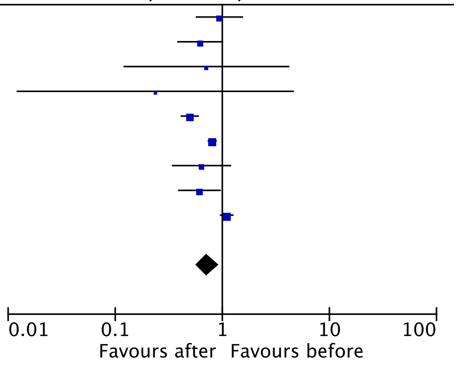

Footnotes

(1) Gill 2009 reported neonatal mortality in NICU 1 as deaths per 1000 admissions

(2) Gill 2009 reported neonatal mortality in NICU 2 as deaths per 1000 admissions

(3) Jain 2021 reported neonatal mortality per 1000 live births

Outcome: All-cause neonatal mortality (sensitivity analysis omitting high risk of bias studies)

Study or Subgroup	After Intervention Events	After Intervention Total	Before Intervention Events	Before Intervention Total	Weight	Risk Ratio IV, Random, 95% CI
Agarwal 2021	56	864	20	290	12.0%	0.94 [0.57, 1.54]
Bassiouny 2020	31	150	20	60	12.3%	0.62 [0.39, 1.00]
Chu 2023	2	90	3	96	1.9%	0.71 [0.12, 4.16]
Feng 2022	0	2901	3	4804	0.7%	0.24 [0.01, 4.58]
Gill 2009 (1)	144	1000	290	1000	19.2%	0.50 [0.41, 0.59]
Gill 2009 (2)	481	1000	598	1000	20.7%	0.80 [0.74, 0.87]
Kommalur 2021	18	58	38	75	13.0%	0.61 [0.39, 0.95]
Lu 2019	388	5786	473	7754	20.1%	1.10 [0.97, 1.25]
**Total (95% CI)**		**11849**		**15079**	**100.0%**	**0.73 [0.57, 0.95]**
Total events	1120		1445			

Heterogeneity: Tau^2 = 0.08; Chi^2 = 53.57, df = 7 (P < 0.00001); I^2 = 87%

Test for overall effect: Z = 2.34 (P = 0.02)

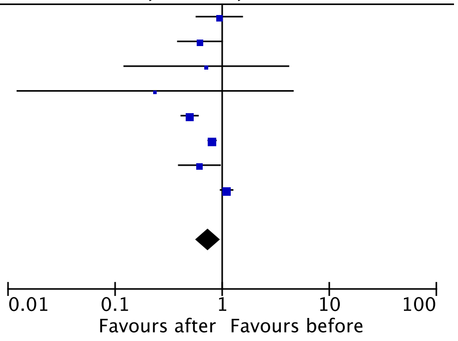

Footnotes

(1) Gill 2009 reported neonatal mortality in NICU 1 as deaths per 1000 admissions

(2) Gill 2009 reported neonatal mortality in NICU 2 as deaths per 1000 admissions

Outcome: Necrotizing enterocolitis (any Bell stage)

Study or Subgroup	After Intervention Events	After Intervention Total	Before Intervention Events	Before Intervention Total	Weight	Risk Ratio IV, Random, 95% CI
Agarwal 2021 (1)	49	864	21	290	8.8%	0.78 [0.48, 1.28]
Chu 2023 (2)	8	90	7	96	2.3%	1.22 [0.46, 3.22]
Feng 2022 (3)	30	2901	54	4804	10.9%	0.92 [0.59, 1.43]
Lu 2019 (4)	249	5786	287	7754	77.9%	1.16 [0.98, 1.37]
**Total (95% CI)**		**9641**		**12944**	**100.0%**	**1.10 [0.95, 1.27]**
Total events	336		369			

Heterogeneity: $Tau^2 = 0.00$; $Chi^2 = 2.91$, df = 3 (P = 0.41); $I^2 = 0\%$

Test for overall effect: Z = 1.22 (P = 0.22)

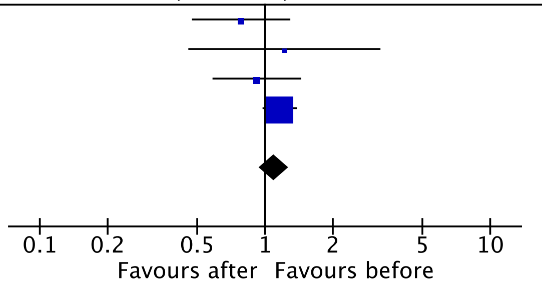

Footnotes

(1) Agarwal 2021 reported overall NEC

(2) Chu 2023 reported NEC (Bell stage ≥ II)

(3) Feng 2022 reported NEC (Bell stage ≥ II)

(4) Lu 2019 reported NEC (Stage ≥ II)

Outcome: Necrotizing enterocolitis (Bell stage ≥ II)

Study or Subgroup	After Intervention Events	After Intervention Total	Before Intervention Events	Before Intervention Total	Weight	Risk Ratio IV, Random, 95% CI
Chu 2023	8	90	7	96	2.5%	1.22 [0.46, 3.22]
Feng 2022	30	2901	54	4804	12.0%	0.92 [0.59, 1.43]
Lu 2019	249	5786	287	7754	85.5%	1.16 [0.98, 1.37]
**Total (95% CI)**		**8777**		**12654**	**100.0%**	**1.13 [0.97, 1.32]**
Total events	287		348			

Heterogeneity: $Tau^2 = 0.00$; $Chi^2 = 0.96$, df = 2 (P = 0.62); $I^2 = 0\%$

Test for overall effect: Z = 1.58 (P = 0.11)

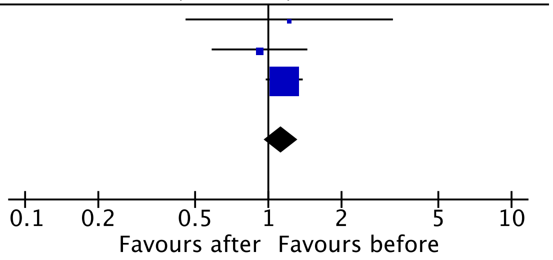

Outcome: Neonatal sepsis (any)

Study or Subgroup	After Intervention Events	After Intervention Total	Before Intervention Events	Before Intervention Total	Weight	Risk Ratio IV, Random, 95% CI
Chu 2023 (1)	10	90	23	96	23.7%	0.46 [0.23, 0.92]
Feng 2022 (2)	10	2901	12	4804	22.0%	1.38 [0.60, 3.19]
Lu 2019 (3)	376	5786	884	7754	27.7%	0.57 [0.51, 0.64]
Maalouf 2023 (4)	39	532	77	153	26.7%	0.15 [0.10, 0.20]
**Total (95% CI)**		**9309**		**12807**	**100.0%**	**0.46 [0.19, 1.09]**
Total events	435		996			

Heterogeneity: $Tau^2 = 0.69$; $Chi^2 = 60.94$, df = 3 ($P < 0.00001$); $I^2 = 95\%$
Test for overall effect: Z = 1.77 (P = 0.08)

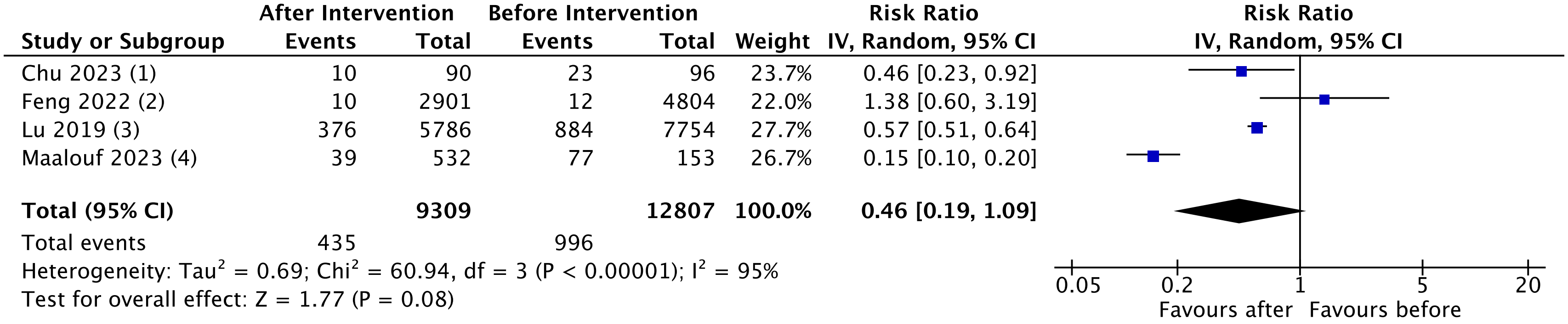

Footnotes
(1) A positive blood or CSF fluid culture, or clinical deterioration and ≥2 abnormal blood indicators or changes in CSF consistent with meningitis
(2) LOS defined as >72 hours of age and positive pathogenic results in blood, urine, or CSF fluid specimens
(3) Late-onset defined as ≥72 hours after birth
(4) EOS defined as ≤72 hours after birth

Outcome: Late-onset sepsis (>72 hours after birth)

Study or Subgroup	After Intervention Events	After Intervention Total	Before Intervention Events	Before Intervention Total	Weight	Risk Ratio IV, Random, 95% CI
Chu 2023	10	90	23	96	25.3%	0.46 [0.23, 0.92]
Feng 2022 (1)	10	2901	12	4804	19.8%	1.38 [0.60, 3.19]
Lu 2019 (2)	376	5786	884	7754	54.8%	0.57 [0.51, 0.64]
**Total (95% CI)**		**8777**		**12654**	**100.0%**	**0.64 [0.41, 1.02]**
Total events	396		919			

Heterogeneity: $Tau^2 = 0.10$; $Chi^2 = 4.59$, df = 2 ($P = 0.10$); $I^2 = 56\%$
Test for overall effect: Z = 1.86 (P = 0.06)

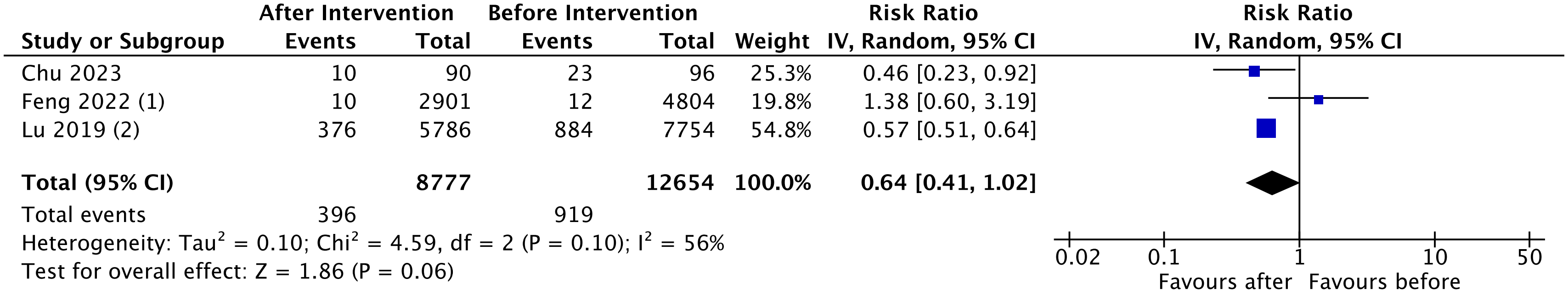

Footnotes
(1) Feng 2022 reported LOS defined as >72 hours of age and positive pathogenic results in blood, urine, or cerebrospinal fluid specimens
(2) Lu 2019 reported LOS and defined late-onset as ≥72 hours after birth

Outcome: Culture-negative sepsis

	After Intervention		Before Intervention			Risk Ratio
**Study or Subgroup**	**Events**	**Total**	**Events**	**Total**	**Weight**	**IV, Random, 95% CI**
Feng 2022 (1)	368	2901	663	4804	73.4%	0.92 [0.82, 1.04]
Lu 2019 (2)	156	5786	248	7754	26.6%	0.84 [0.69, 1.03]
**Total (95% CI)**		**8687**		**12558**	**100.0%**	**0.90 [0.81, 0.99]**
Total events	524		911			

Heterogeneity: $Tau^2 = 0.00; Chi^2 = 0.54, df = 1 (P = 0.46); I^2 = 0\%$
Test for overall effect: Z = 2.07 (P = 0.04)

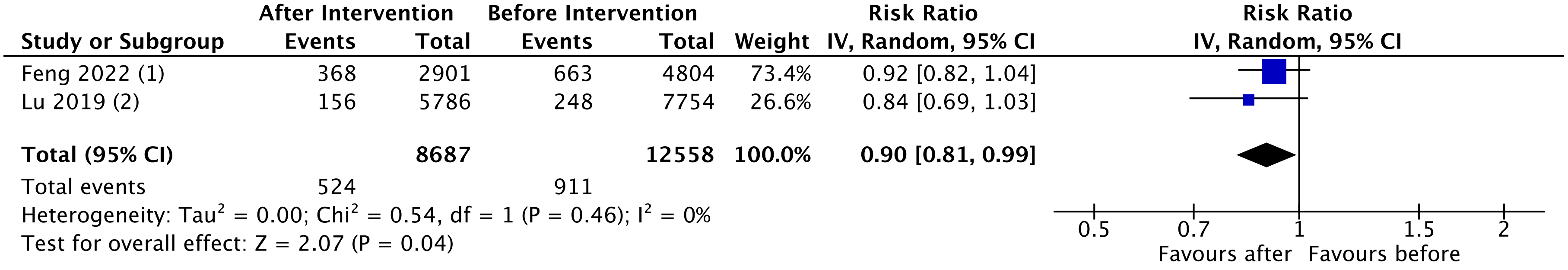

Footnotes
(1) Feng 2022 reported infants treated in ≤5 days for culture-negative sepsis
(2) Lu 2019 reported all infants treated for culture-negative sepsis

Outcome: Pneumonia

	After Intervention		Before Intervention			Risk Ratio
**Study or Subgroup**	**Events**	**Total**	**Events**	**Total**	**Weight**	**IV, Random, 95% CI**
Feng 2022 (1)	128	2901	296	4804	47.9%	0.72 [0.59, 0.88]
Lu 2019 (2)	434	5786	543	7754	52.1%	1.07 [0.95, 1.21]
**Total (95% CI)**		**8687**		**12558**	**100.0%**	**0.88 [0.60, 1.31]**
Total events	562		839			

Heterogeneity: $Tau^2 = 0.07; Chi^2 = 11.19, df = 1 (P = 0.0008); I^2 = 91\%$
Test for overall effect: Z = 0.62 (P = 0.54)

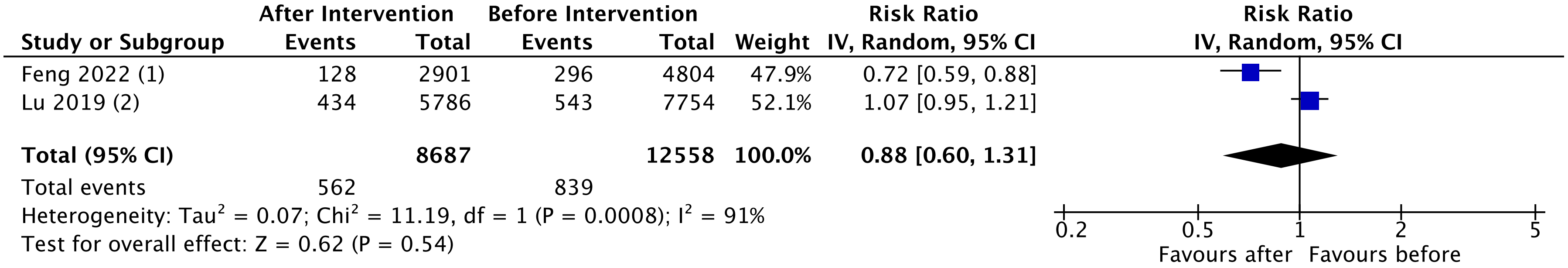

Footnotes
(1) Feng 2022 reported infants treated in ≤5 days for pneumonia
(2) Lu 2019 reported all infants treated for pneumonia

Outcome: Multidrug-resistant organism infections or colonizations

	After Intervention		Before Intervention			Risk Ratio
**Study or Subgroup**	**Events**	**Total**	**Events**	**Total**	**Weight**	**IV, Random, 95% CI**
Feng 2022 (1)	3	2901	7	4804	5.2%	0.71 [0.18, 2.74]
Lu 2019 (2)	58	5786	109	7754	94.8%	0.71 [0.52, 0.98]
**Total (95% CI)**		**8687**		**12558**	**100.0%**	**0.71 [0.52, 0.97]**
Total events	61		116			

Heterogeneity: $Tau^2 = 0.00$; $Chi^2 = 0.00$, df = 1 (P = 0.99); $I^2 = 0\%$
Test for overall effect: Z = 2.15 (P = 0.03)

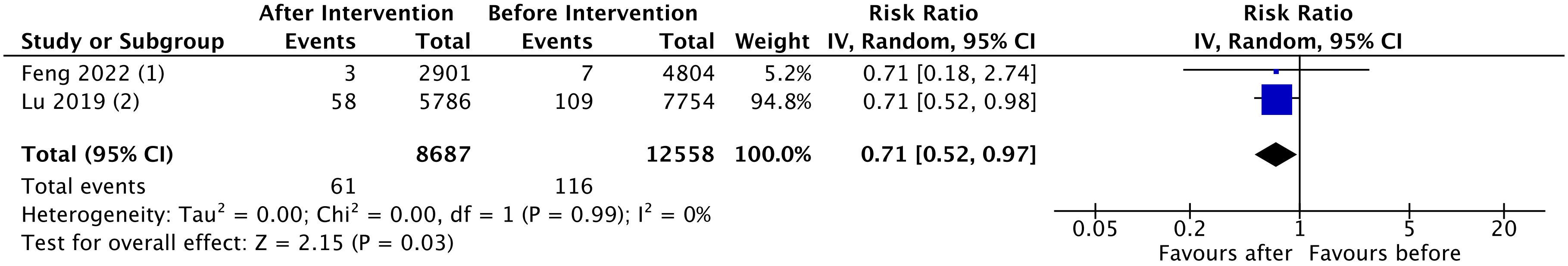

Footnotes
(1) Feng 2022 reported multidrug–resistant organism infections
(2) Lu 2019 reported multidrug–resistant organism colonizations

Outcome: Bloodstream isolates of methicillin-resistant *Staphylococcus aureus* (MRSA)

	After Intervention		Before Intervention			Risk Ratio
**Study or Subgroup**	**Events**	**Total**	**Events**	**Total**	**Weight**	**IV, Random, 95% CI**
Bassiouny 2020	4	58	0	20	34.4%	3.20 [0.18, 57.01]
Gill 2009	1	776	0	486	27.9%	1.88 [0.08, 46.06]
Kommalur 2021	1	58	1	75	37.7%	1.29 [0.08, 20.24]
**Total (95% CI)**		**892**		**581**	**100.0%**	**1.96 [0.36, 10.62]**
Total events	6		1			

Heterogeneity: $Tau^2 = 0.00$; $Chi^2 = 0.20$, df = 2 (P = 0.90); $I^2 = 0\%$
Test for overall effect: Z = 0.78 (P = 0.43)

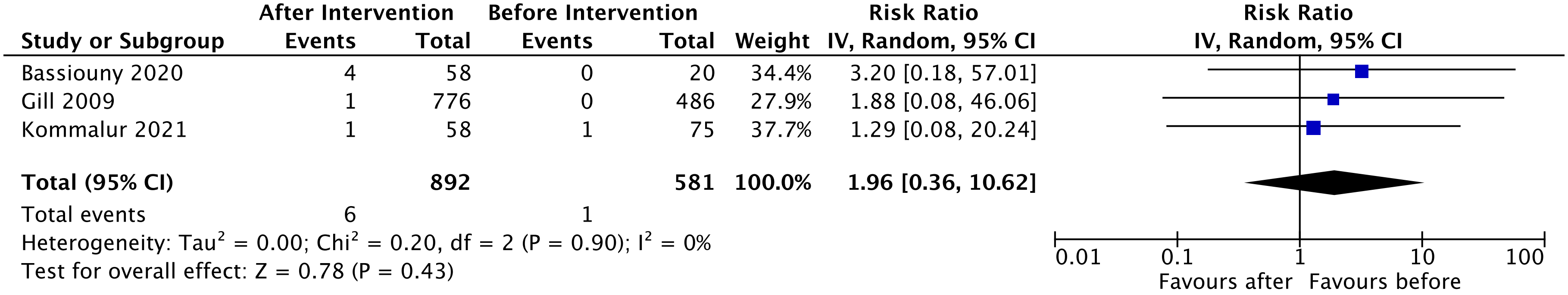

Outcome: Bloodstream isolates of *Klebsiella* spp.

Study or Subgroup	After Intervention Events	After Intervention Total	Before Intervention Events	Before Intervention Total	Weight	Risk Ratio IV, Random, 95% CI
Bassiouny 2020	12	58	8	20	12.6%	0.52 [0.25, 1.08]
Gill 2009	87	776	76	486	83.4%	0.72 [0.54, 0.95]
Kommalur 2021 (1)	3	58	7	75	4.0%	0.55 [0.15, 2.05]
**Total (95% CI)**		**892**		**581**	**100.0%**	**0.68 [0.52, 0.88]**
Total events	102		91			

Heterogeneity: $Tau^2 = 0.00$; $Chi^2 = 0.76$, df = 2 (P = 0.69); $I^2 = 0\%$
Test for overall effect: Z = 2.88 (P = 0.004)

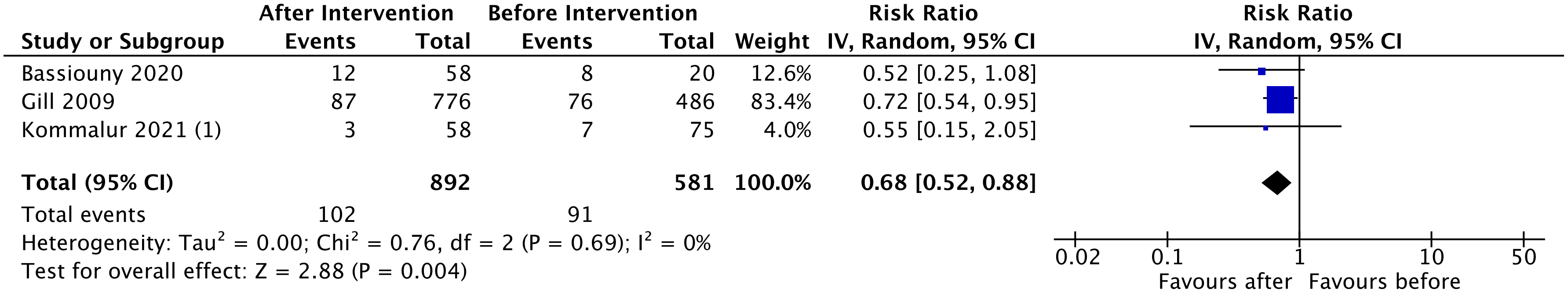

Footnotes
(1) Kommalur 2021 reported *Klebsiella pneumoniae* organisms

Outcome: Bloodstream isolates of *Acinetobacter* spp.

Study or Subgroup	After Intervention Events	After Intervention Total	Before Intervention Events	Before Intervention Total	Weight	Risk Ratio IV, Random, 95% CI
Bassiouny 2020	8	58	1	20	26.0%	2.76 [0.37, 20.71]
Gill 2009 (1)	5	776	3	486	51.5%	1.04 [0.25, 4.35]
Kommalur 2021 (2)	1	58	4	75	22.6%	0.32 [0.04, 2.82]
**Total (95% CI)**		**892**		**581**	**100.0%**	**1.03 [0.37, 2.89]**
Total events	14		8			

Heterogeneity: $Tau^2 = 0.01$; $Chi^2 = 2.02$, df = 2 (P = 0.36); $I^2 = 1\%$
Test for overall effect: Z = 0.06 (P = 0.95)

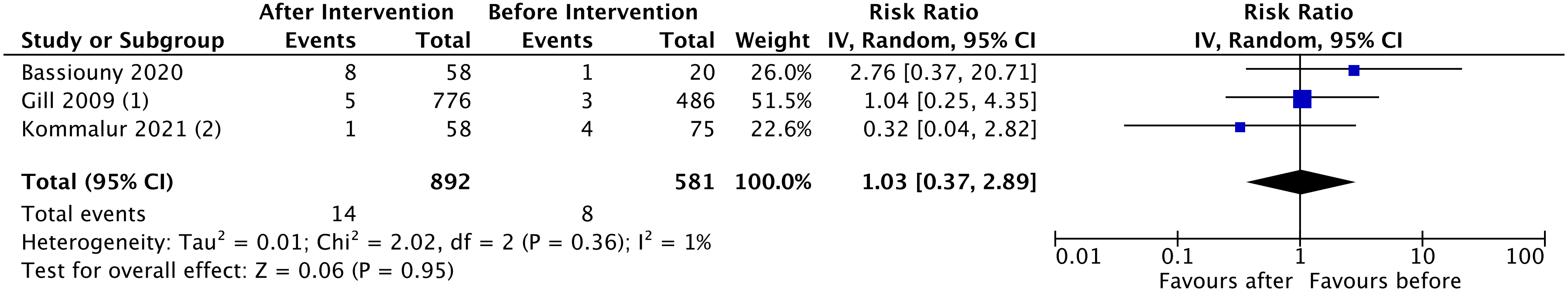

Footnotes
(1) Gill 2009 reported *Acinetobacter baumanii* organisms
(2) Kommalur 2021 reported *Acinetobacter baumanii* organisms

Outcome: Bloodstream isolates of *Escherichia coli* (*E. coli*)

	After Intervention		Before Intervention			Risk Ratio
**Study or Subgroup**	**Events**	**Total**	**Events**	**Total**	**Weight**	**IV, Random, 95% CI**
Bassiouny 2020	4	58	0	20	24.6%	3.20 [0.18, 57.01]
Gill 2009	8	776	16	486	53.7%	0.31 [0.14, 0.73]
Kommalur 2021	1	58	0	75	21.7%	3.86 [0.16, 93.16]
**Total (95% CI)**		**892**		**581**	**100.0%**	**0.96 [0.15, 6.11]**
Total events	13		16			

Heterogeneity: $Tau^2 = 1.48$; $Chi^2 = 4.24$, df = 2 (P = 0.12); $I^2 = 53\%$
Test for overall effect: Z = 0.05 (P = 0.96)

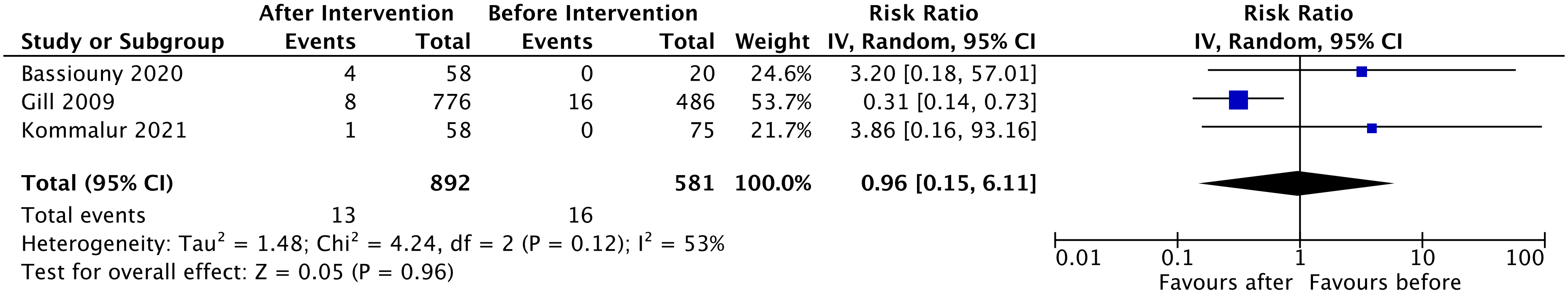

Outcome: Bloodstream isolates of *Enterobacter* spp.

	After Intervention		Before Intervention			Risk Ratio
**Study or Subgroup**	**Events**	**Total**	**Events**	**Total**	**Weight**	**IV, Random, 95% CI**
Bassiouny 2020	4	58	0	20	9.2%	3.20 [0.18, 57.01]
Gill 2009	48	776	47	486	90.8%	0.64 [0.43, 0.94]
Kommalur 2021	0	58	0	75		Not estimable
**Total (95% CI)**		**892**		**581**	**100.0%**	**0.74 [0.30, 1.85]**
Total events	52		47			

Heterogeneity: $Tau^2 = 0.20$; $Chi^2 = 1.18$, df = 1 (P = 0.28); $I^2 = 15\%$
Test for overall effect: Z = 0.64 (P = 0.52)

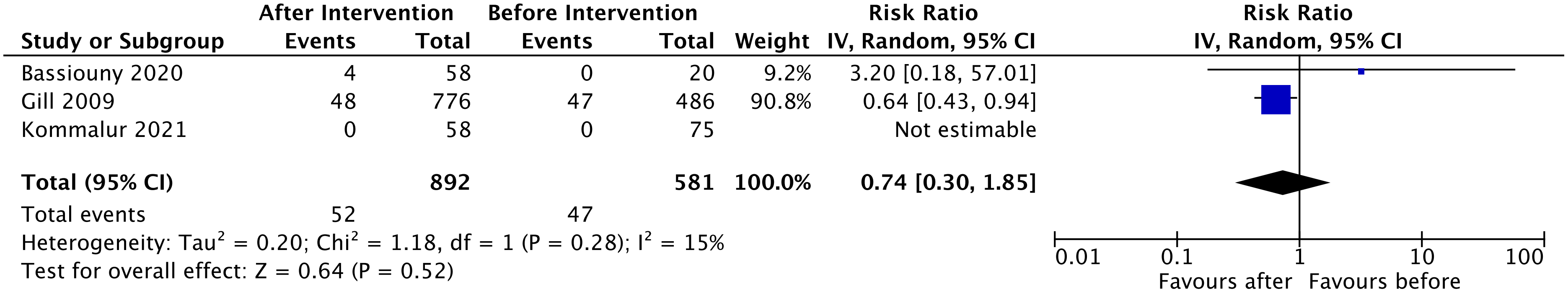

Outcome: Bloodstream isolates of coagulase-negative *staphylococci* (CoNS)

	After Intervention		Before Intervention			Risk Ratio
**Study or Subgroup**	**Events**	**Total**	**Events**	**Total**	**Weight**	**IV, Random, 95% CI**
Bassiouny 2020	14	58	1	20	10.0%	4.83 [0.68, 34.41]
Gill 2009	34	776	11	486	86.1%	1.94 [0.99, 3.78]
Kommalur 2021	0	58	1	75	3.8%	0.43 [0.02, 10.35]
**Total (95% CI)**		**892**		**581**	**100.0%**	**2.00 [1.08, 3.73]**
Total events	48		13			

Heterogeneity: $Tau^2 = 0.00$; $Chi^2 = 1.68$, df = 2 (P = 0.43); $I^2 = 0\%$
Test for overall effect: Z = 2.19 (P = 0.03)

Risk Ratio
IV, Random, 95% CI

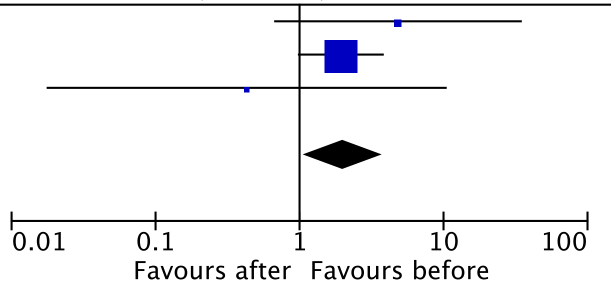

Outcome: Bloodstream isolates of *Pseudomonas* spp.

	After Intervention		Before Intervention			Risk Ratio
**Study or Subgroup**	**Events**	**Total**	**Events**	**Total**	**Weight**	**IV, Random, 95% CI**
Bassiouny 2020	1	58	0	20	2.4%	1.07 [0.05, 25.21]
Gill 2009	20	776	52	486	95.2%	0.24 [0.15, 0.40]
Kommalur 2021 (1)	0	58	1	75	2.4%	0.43 [0.02, 10.35]
**Total (95% CI)**		**892**		**581**	**100.0%**	**0.25 [0.15, 0.41]**
Total events	21		53			

Heterogeneity: $Tau^2 = 0.00$; $Chi^2 = 0.94$, df = 2 (P = 0.63); $I^2 = 0\%$
Test for overall effect: Z = 5.49 (P < 0.00001)

Risk Ratio
IV, Random, 95% CI

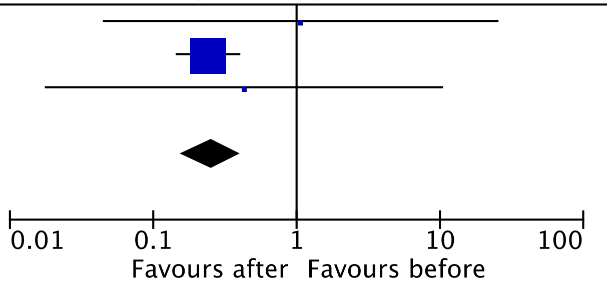

Footnotes
(1) Kommalur 2021 reported *Pseudomonas aeruginosa* organisms

Outcome: Bloodstream isolates of *Candida* spp.

Study or Subgroup	After Intervention Events	After Intervention Total	Before Intervention Events	Before Intervention Total	Weight	Risk Ratio IV, Random, 95% CI
Bassiouny 2020	3	58	8	20	87.1%	0.13 [0.04, 0.44]
Kommalur 2021	0	58	1	75	12.9%	0.43 [0.02, 10.35]
**Total (95% CI)**		**116**		**95**	**100.0%**	**0.15 [0.05, 0.47]**
Total events	3		9			

Heterogeneity: $Tau^2 = 0.00$; $Chi^2 = 0.48$, df = 1 (P = 0.49); $I^2 = 0\%$
Test for overall effect: Z = 3.24 (P = 0.001)

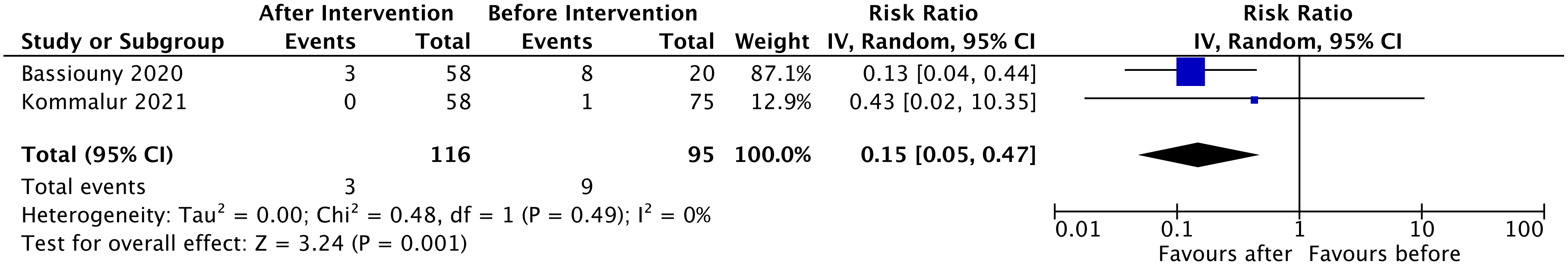

Outcome: Mean length of hospital stay, days

Study or Subgroup	After Intervention Mean	After Intervention SD	After Intervention Total	Before Intervention Mean	Before Intervention SD	Before Intervention Total	Weight	Mean Difference IV, Random, 95% CI
Bassiouny 2020	11.94	10.71	150	15.15	11.38	60	49.9%	−3.21 [−6.56, 0.14]
Maalouf 2023	41.6	31.8	563	23.6	5.5	153	50.1%	18.00 [15.23, 20.77]
**Total (95% CI)**			**713**			**213**	**100.0%**	**7.42 [−13.37, 28.20]**

Heterogeneity: $Tau^2 = 222.47$; $Chi^2 = 91.49$, df = 1 (P < 0.00001); $I^2 = 99\%$
Test for overall effect: Z = 0.70 (P = 0.48)

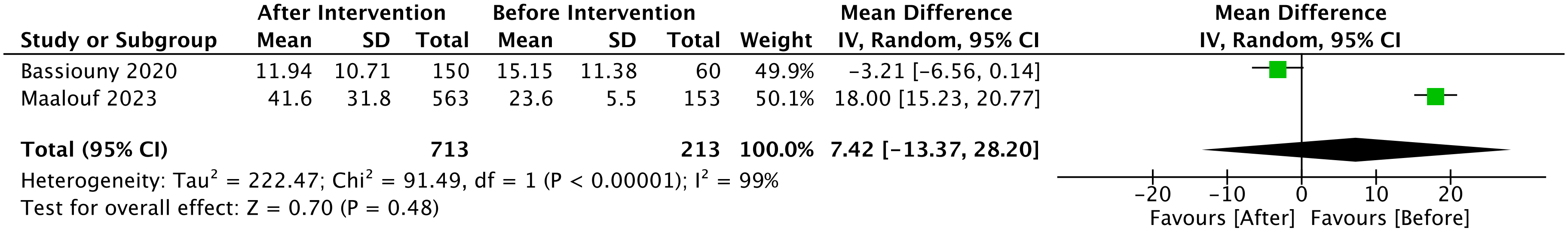

Outcome: Mean length of hospital stay, days (including *Feng 2022* [35])

	After Intervention			Before Intervention				Mean Difference
**Study or Subgroup**	**Mean**	**SD**	**Total**	**Mean**	**SD**	**Total**	**Weight**	**IV, Random, 95% CI**
Bassiouny 2020	11.94	10.71	150	15.15	11.38	60	32.9%	−3.21 [−6.56, 0.14]
Feng 2022 (1)	7	2.96	2901	7	3.7	4804	33.9%	0.00 [−0.15, 0.15]
Maalouf 2023	41.6	31.8	563	23.6	5.5	153	33.2%	18.00 [15.23, 20.77]
**Total (95% CI)**			**3614**			**5017**	**100.0%**	**4.92 [−6.34, 16.18]**

Heterogeneity: $Tau^2 = 97.36$; $Chi^2 = 165.66$, df = 2 (P < 0.00001); $I^2 = 99\%$
Test for overall effect: Z = 0.86 (P = 0.39)

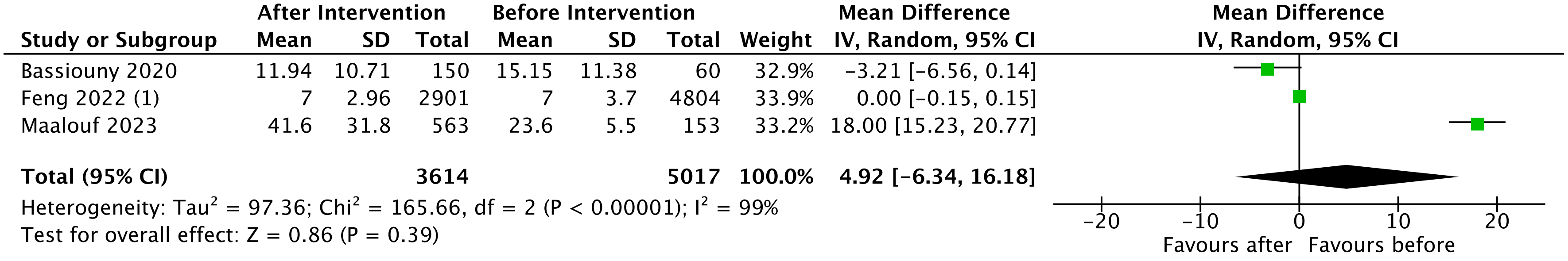

Footnotes
(1) Feng 2022 expressed length of hospital stay as median and IQR. assuming the distribution of data is symmetrical, we estimated mean and SD

Outcome: Number of newborns receiving antibiotics

	After Intervention		Before Intervention			Risk Ratio
**Study or Subgroup**	**Events**	**Total**	**Events**	**Total**	**Weight**	**IV, Random, 95% CI**
Agarwal 2021 (1)	544	864	226	290	21.8%	0.81 [0.75, 0.88]
Chu 2023 (2)	60	90	95	96	18.7%	0.67 [0.58, 0.78]
Feng 2022 (3)	1341	2901	3153	4804	22.9%	0.70 [0.67, 0.74]
Kommalur 2021 (4)	44	58	63	75	17.2%	0.90 [0.76, 1.08]
Maalouf 2023 (5)	208	532	118	153	19.3%	0.51 [0.44, 0.58]
**Total (95% CI)**		**4445**		**5418**	**100.0%**	**0.71 [0.61, 0.81]**
Total events	2197		3655			

Heterogeneity: $Tau^2 = 0.02$; $Chi^2 = 41.16$, df = 4 (P < 0.00001); $I^2 = 90\%$
Test for overall effect: Z = 4.84 (P < 0.00001)

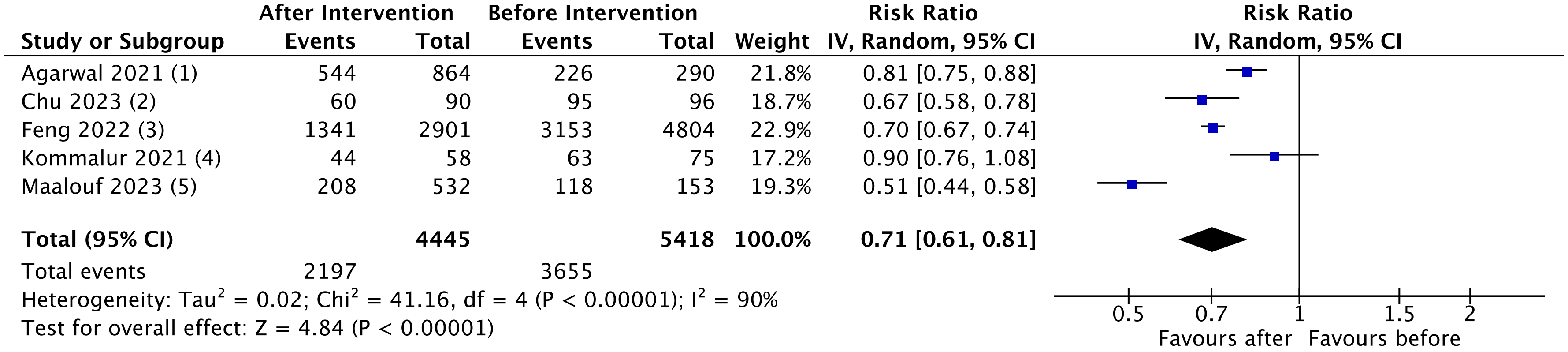

Footnotes
(1) Agarwal 2021 reported neonates unexposed to antibiotics (from which the neonates exposed to antibiotics was derived)
(2) Chu 2023 reported proportion of early antibiotic usage
(3) Feng 2022 reported proportion of antibiotic exposure
(4) Kommalur 2021 reported percentage of neonates with no antibiotics (from which neonates receiving antibiotics was derived)
(5) Maalouf 2023 reported proportion of neonates treated for early-onset sepsis

Outcome: Duration of antibiotic therapy >5 days

Study or Subgroup	After Intervention Events	After Intervention Total	Before Intervention Events	Before Intervention Total	Weight	Risk Ratio IV, Random, 95% CI
Chu 2023 (1)	19	90	92	96	47.8%	0.22 [0.15, 0.33]
Feng 2022 (2)	654	2901	1882	4804	52.2%	0.58 [0.53, 0.62]
**Total (95% CI)**		**2991**		**4900**	**100.0%**	**0.36 [0.14, 0.93]**
Total events	673		1974			

Heterogeneity: $Tau^2 = 0.44$; $Chi^2 = 21.21$, df = 1 ($P < 0.00001$); $I^2 = 95\%$
Test for overall effect: Z = 2.11 (P = 0.03)

Risk Ratio IV, Random, 95% CI

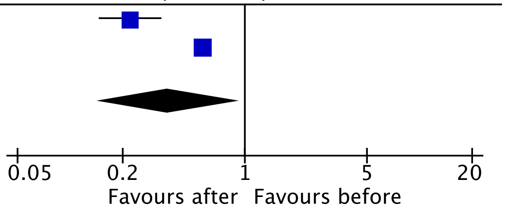

Footnotes
(1) Chu 2023 reported the proportion of neonates treated with an initial antibiotic course >7 days
(2) Feng 2022 reported the proportion of neonates treated with a duration of therapy >5 days

Outcome: Neonates with antibiotics discontinued after 48 hours

Study or Subgroup	After Intervention Events	After Intervention Total	Before Intervention Events	Before Intervention Total	Weight	Risk Ratio IV, Random, 95% CI
Kommalur 2021	11	58	0	75	30.6%	29.63 [1.78, 492.56]
Lu 2019	5497	5786	2481	7754	69.4%	2.97 [2.87, 3.07]
**Total (95% CI)**		**5844**		**7829**	**100.0%**	**6.00 [0.75, 47.88]**
Total events	5508		2481			

Heterogeneity: $Tau^2 = 1.62$; $Chi^2 = 2.57$, df = 1 ($P = 0.11$); $I^2 = 61\%$
Test for overall effect: Z = 1.69 (P = 0.09)

Risk Ratio IV, Random, 95% CI

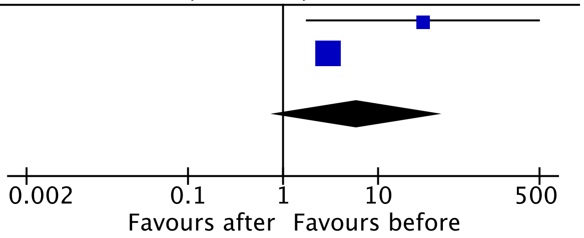

Appendix M: Forest plots for risk differences (RDs)

Regulation

Outcome: Sepsis/suspected sepsis (by study design)

	After Intervention		Before Intervention			Risk Difference
**Study or Subgroup**	**Events**	**Total**	**Events**	**Total**	**Weight**	**IV, Random, 95% CI**
**1.2.1 Observational**						
Akintan 2024 (1)	12	19	5	19	42.1%	0.37 [0.07, 0.66]
**Subtotal (95% CI)**		**19**		**19**	**42.1%**	**0.37 [0.07, 0.66]**
Total events	12		5			
Heterogeneity: Not applicable						
Test for overall effect: Z = 2.46 (P = 0.01)						
**1.2.2 Quasi-experimental**						
Wang 2020 (2)	356	4488	417	4743	57.9%	-0.01 [-0.02, 0.00]
**Subtotal (95% CI)**		**4488**		**4743**	**57.9%**	**-0.01 [-0.02, 0.00]**
Total events	356		417			
Heterogeneity: Not applicable						
Test for overall effect: Z = 1.49 (P = 0.14)						
**Total (95% CI)**		**4507**		**4762**	**100.0%**	**0.15 [-0.21, 0.52]**
Total events	368		422			
Heterogeneity: $Tau^2 = 0.06$; $Chi^2 = 6.32$, df = 1 (P = 0.01); $I^2 = 84\%$						
Test for overall effect: Z = 0.81 (P = 0.42)						
Test for subgroup differences: $Chi^2 = 6.32$, df = 1 (P = 0.01), $I^2 = 84.2\%$						

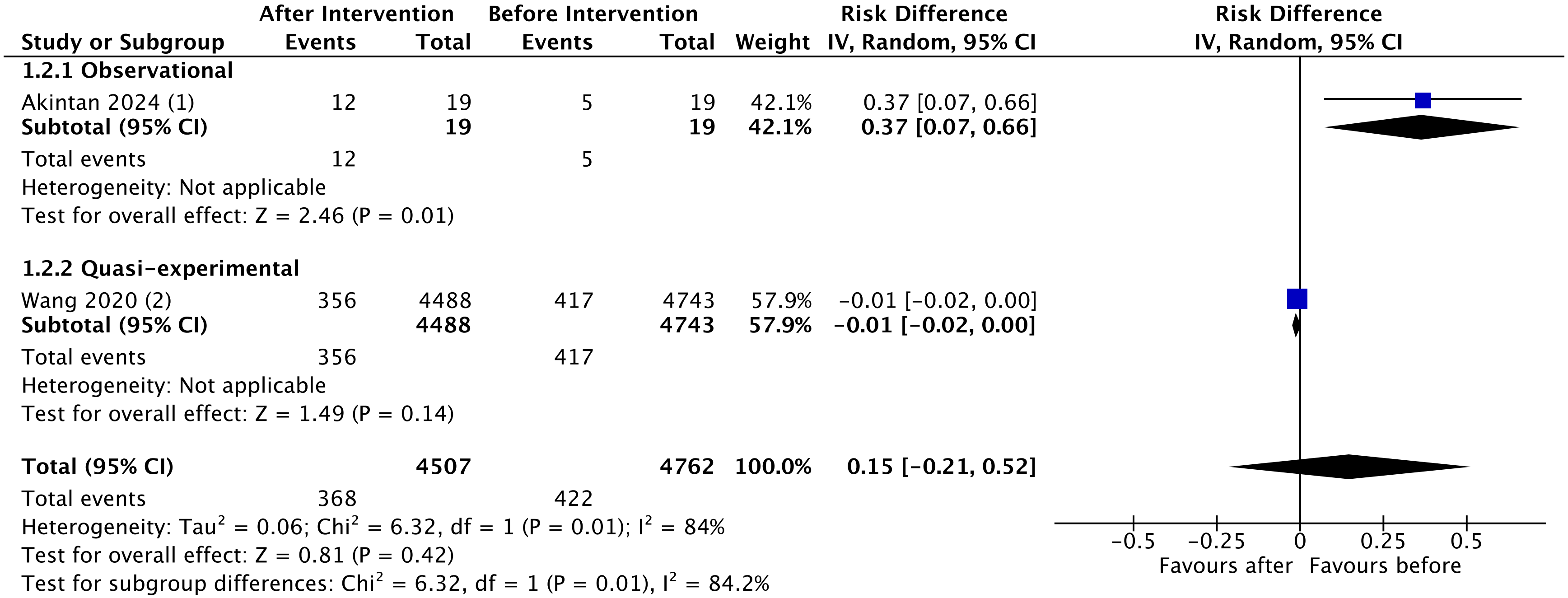

Footnotes
(1) Akintan 2024 reported sepsis among inpatient neonates
(2) Wang 2020 reported sepsis/suspected sepsis among inpatient neonates

Outcome: Number of neonates receiving at least one antimicrobial (by study design)

Study or Subgroup	After Intervention Events	After Intervention Total	Before Intervention Events	Before Intervention Total	Weight	Risk Difference IV, Random, 95% CI
**1.4.1 Observational**						
Akintan 2024	17	19	15	19	42.8%	0.11 [−0.12, 0.33]
**Subtotal (95% CI)**		**19**		**19**	**42.8%**	**0.11 [−0.12, 0.33]**
Total events	17		15			
Heterogeneity: Not applicable						
Test for overall effect: Z = 0.90 (P = 0.37)						
**1.4.2 Quasi−experimental**						
Wang 2020	3328	4488	4478	4743	57.2%	−0.20 [−0.22, −0.19]
**Subtotal (95% CI)**		**4488**		**4743**	**57.2%**	**−0.20 [−0.22, −0.19]**
Total events	3328		4478			
Heterogeneity: Not applicable						
Test for overall effect: Z = 27.61 (P < 0.00001)						
**Total (95% CI)**		**4507**		**4762**	**100.0%**	**−0.07 [−0.37, 0.23]**
Total events	3345		4493			
Heterogeneity: $Tau^2 = 0.04$; $Chi^2 = 6.89$, df = 1 (P = 0.009); $I^2 = 85\%$						
Test for overall effect: Z = 0.47 (P = 0.64)						
Test for subgroup differences: $Chi^2 = 6.89$, df = 1 (P = 0.009), $I^2 = 85.5\%$						

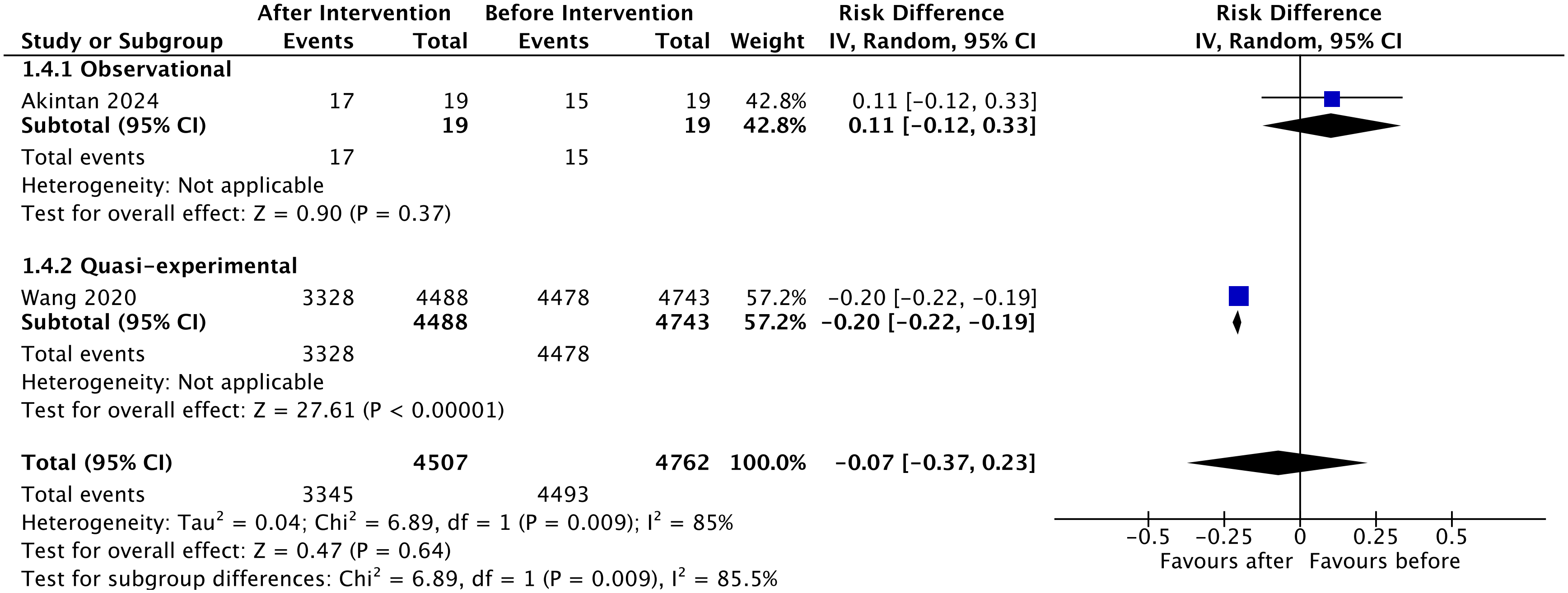

Optimization

Outcome: All-cause neonatal mortality

Study or Subgroup	After Intervention Events	After Intervention Total	Before Intervention Events	Before Intervention Total	Weight	Risk Difference IV, Random, 95% CI
Sathyan 2022	1	43	1	40	4.3%	−0.00 [−0.07, 0.06]
Sowjanya 2018	12	428	10	429	42.3%	0.00 [−0.02, 0.03]
Xiao−Lu 2016	5	470	2	168	53.4%	−0.00 [−0.02, 0.02]
**Total (95% CI)**		**941**		**637**	**100.0%**	**0.00 [−0.01, 0.02]**
Total events	18		13			
Heterogeneity: $Tau^2 = 0.00$; $Chi^2 = 0.18$, df = 2 (P = 0.91); $I^2 = 0\%$						
Test for overall effect: Z = 0.18 (P = 0.86)						

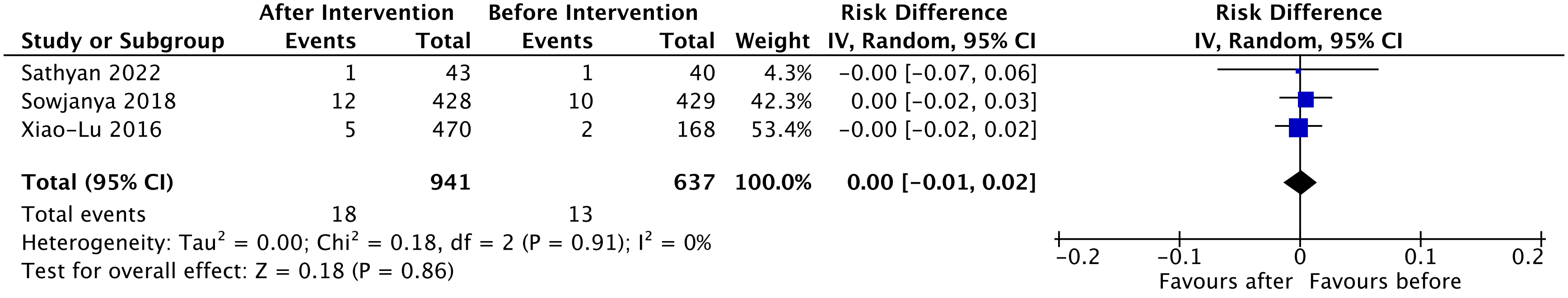

Outcome: Culture-positive sepsis

	After Intervention		Before Intervention			Risk Difference
**Study or Subgroup**	**Events**	**Total**	**Events**	**Total**	**Weight**	**IV, Random, 95% CI**
Sowjanya 2018	14	428	18	429	54.3%	−0.01 [−0.03, 0.02]
Xiao-Lu 2016	144	470	77	168	45.7%	−0.15 [−0.24, −0.07]
**Total (95% CI)**		**898**		**597**	**100.0%**	**-0.07 [-0.21, 0.06]**
Total events	158		95			

Heterogeneity: $Tau^2 = 0.01$; $Chi^2 = 9.71$, df = 1 (P = 0.002); $I^2 = 90\%$

Test for overall effect: Z = 1.05 (P = 0.30)

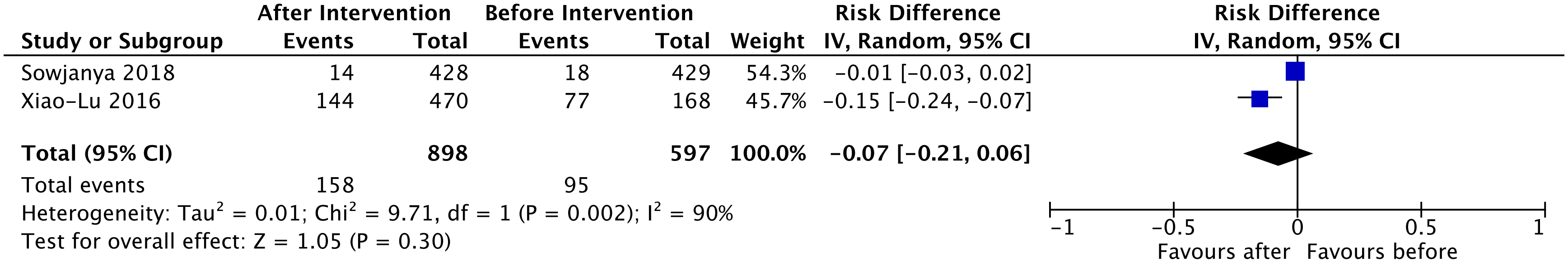

Outcome: Number of neonates on antibiotics

	After Intervention		Before Intervention			Risk Difference
**Study or Subgroup**	**Events**	**Total**	**Events**	**Total**	**Weight**	**IV, Random, 95% CI**
Jinka 2017	584	1276	681	1176	35.2%	-0.12 [-0.16, -0.08]
Sowjanya 2018	370	428	385	429	34.1%	-0.03 [-0.08, 0.01]
Xiao-Lu 2016	372	470	154	168	30.7%	-0.13 [-0.18, -0.07]
**Total (95% CI)**		**2174**		**1773**	**100.0%**	**-0.09 [-0.15, -0.03]**
Total events	1326		1220			

Heterogeneity: $Tau^2 = 0.00$; $Chi^2 = 10.65$, df = 2 (P = 0.005); $I^2 = 81\%$

Test for overall effect: Z = 2.98 (P = 0.003)

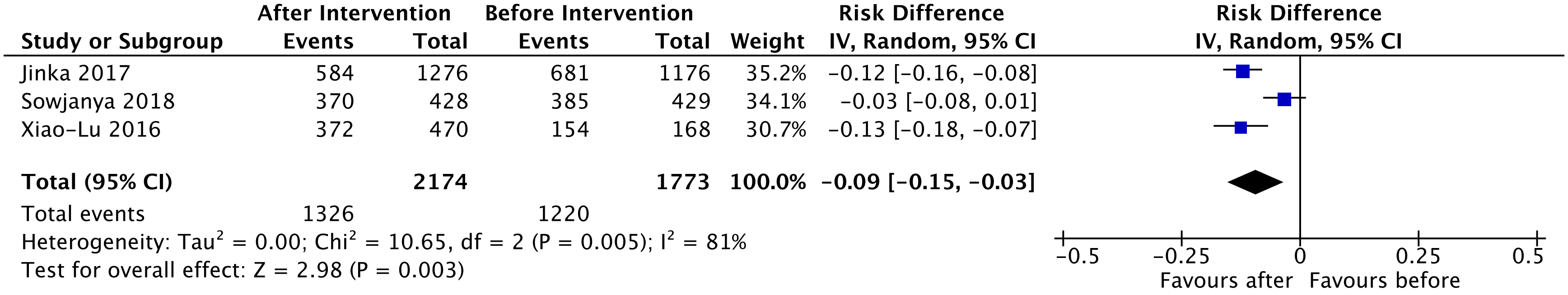

Outcome: AWaRe[63] antibiotic usage

Study or Subgroup	After Intervention Events	After Intervention Total	Before Intervention Events	Before Intervention Total	Weight	Risk Difference IV, Random, 95% CI
**1.3.1 Access group**						
Jinka 2017 – Amikacin	116	584	301	681	6.7%	−0.24 [−0.29, −0.19]
Jinka 2017 – Ampicillin/Gentamicin	491	584	449	681	6.7%	0.18 [0.14, 0.23]
Sowjanya 2018 – Amikacin	160	428	190	429	6.6%	−0.07 [−0.13, −0.00]
Sowjanya 2018 –Ampicillin	40	428	45	429	6.8%	−0.01 [−0.05, 0.03]
Xiao–Lu 2016 – Penicillin	346	470	156	168	6.7%	−0.19 [−0.25, −0.14]
**Subtotal (95% CI)**		**2494**		**2388**	**33.5%**	**−0.07 [−0.22, 0.09]**
Total events	1153		1141			
Heterogeneity: Tau² = 0.03; Chi² = 183.68, df = 4 (P < 0.00001); I² = 98%						
Test for overall effect: Z = 0.86 (P = 0.39)						
**1.3.2 Watch group**						
Jinka 2017 – 3rd Gen. Cephalosporins	39	584	281	681	6.8%	−0.35 [−0.39, −0.30]
Jinka 2017 – Ciprofloxacin	45	584	5	681	6.8%	0.07 [0.05, 0.09]
Jinka 2017 – Meropenem	58	584	58	681	6.8%	0.01 [−0.02, 0.05]
Jinka 2017 – Piperacillin–Tazobactam	81	584	109	681	6.8%	−0.02 [−0.06, 0.02]
Sowjanya 2018 – Piperacillin–Tazobactam	155	428	175	429	6.6%	−0.05 [−0.11, 0.02]
Xiao–Lu 2016 – 3rd and 4th Gen. Cephalosporins	187	470	90	168	6.4%	−0.14 [−0.23, −0.05]
Xiao–Lu 2016 – Carbapenems	168	470	27	168	6.6%	0.20 [0.13, 0.27]
Xiao–Lu 2016 – Glycopeptides	72	470	32	168	6.6%	−0.04 [−0.11, 0.03]
**Subtotal (95% CI)**		**4174**		**3657**	**53.4%**	**−0.04 [−0.14, 0.07]**
Total events	805		777			
Heterogeneity: Tau² = 0.02; Chi² = 337.34, df = 7 (P < 0.00001); I² = 98%						
Test for overall effect: Z = 0.71 (P = 0.48)						
**1.3.3 Mix of Access and Watch group**						
Sowjanya 2018 – Empirical 1st Line (1)	175	428	195	429	6.6%	−0.05 [−0.11, 0.02]
Xiao–Lu 2016 – 1st and 2nd Gen. Cephalosporins	100	470	126	168	6.5%	−0.54 [−0.61, −0.46]
**Subtotal (95% CI)**		**898**		**597**	**13.1%**	**−0.29 [−0.77, 0.19]**
Total events	275		321			
Heterogeneity: Tau² = 0.12; Chi² = 92.40, df = 1 (P < 0.00001); I² = 99%						
Test for overall effect: Z = 1.18 (P = 0.24)						
**Total (95% CI)**		**7566**		**6642**	**100.0%**	**−0.08 [−0.17, 0.00]**
Total events	2233		2239			
Heterogeneity: Tau² = 0.03; Chi² = 704.74, df = 14 (P < 0.00001); I² = 98%						
Test for overall effect: Z = 1.85 (P = 0.06)						
Test for subgroup differences: Chi² = 1.04, df = 2 (P = 0.59), I² = 0%						

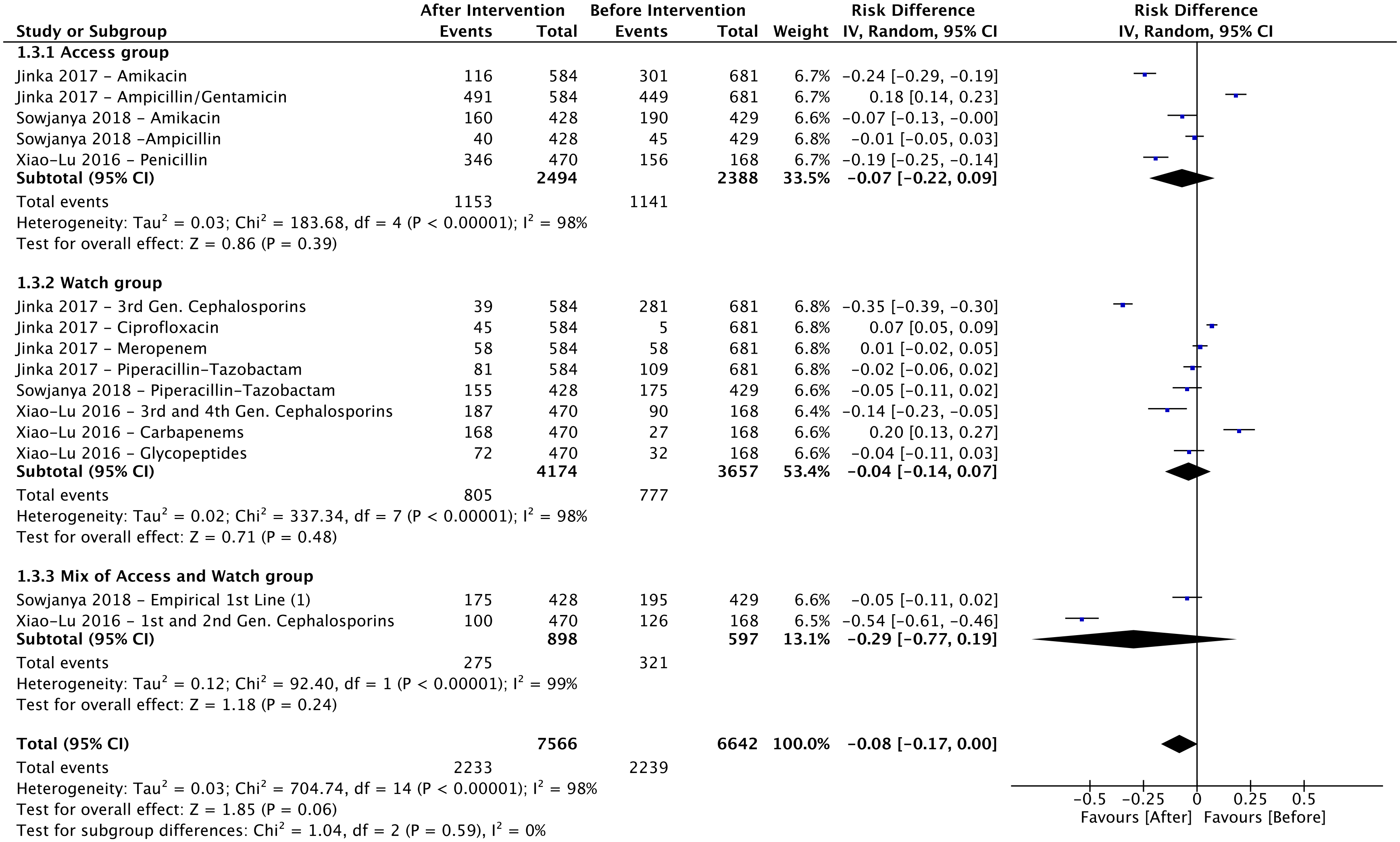

Footnotes
(1) Amikacin, Ampicillin, and Piperacillin–Tazobactam

Regulation and Optimization

Outcome: Neonatal mortality due to nosocomial bloodstream infection (by level of care)

	After Intervention		Before Intervention			Risk Difference
**Study or Subgroup**	**Events**	**Total**	**Events**	**Total**	**Weight**	**IV, Random, 95% CI**
**1.37.1 Secondary**						
Landre–Peigne 2021	1	148	1	125	49.3%	−0.00 [−0.02, 0.02]
**Subtotal (95% CI)**		**148**		**125**	**49.3%**	**−0.00 [−0.02, 0.02]**
Total events	1		1			
Heterogeneity: Not applicable						
Test for overall effect: Z = 0.12 (P = 0.91)						
**1.37.2 Tertiary or higher**						
El–Baky 2020 (1)	13	617	16	462	50.7%	−0.01 [−0.03, 0.01]
**Subtotal (95% CI)**		**617**		**462**	**50.7%**	**−0.01 [−0.03, 0.01]**
Total events	13		16			
Heterogeneity: Not applicable						
Test for overall effect: Z = 1.32 (P = 0.19)						
**Total (95% CI)**		**765**		**587**	**100.0%**	**−0.01 [−0.02, 0.01]**
Total events	14		17			

Heterogeneity: $Tau^2 = 0.00$; $Chi^2 = 0.71$, df = 1 (P = 0.40); $I^2 = 0\%$

Test for overall effect: Z = 1.02 (P = 0.31)

Test for subgroup differences: $Chi^2 = 0.71$, df = 1 (P = 0.40), $I^2 = 0\%$

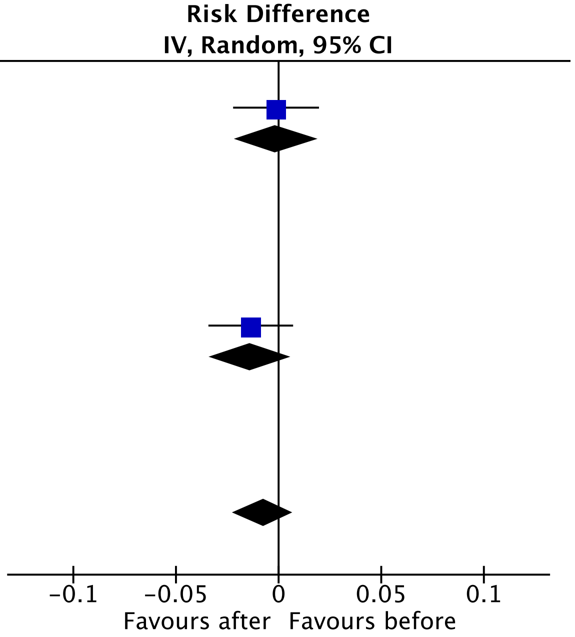

Footnotes

(1) Data updated through direct correspondance with study authors

Regulation, Education, and Optimization

Outcome: All-cause neonatal mortality

Study or Subgroup	After Intervention Events	After Intervention Total	Before Intervention Events	Before Intervention Total	Weight	Risk Difference IV, Random, 95% CI
Agarwal 2021	56	864	20	290	12.1%	-0.00 [-0.04, 0.03]
Bassiouny 2020	31	150	20	60	1.9%	-0.13 [-0.26, 0.01]
Chu 2023	2	90	3	96	9.2%	-0.01 [-0.06, 0.04]
Feng 2022	0	2901	3	4804	18.6%	-0.00 [-0.00, 0.00]
Gill 2009 (1)	144	1000	290	1000	11.6%	-0.15 [-0.18, -0.11]
Gill 2009 (2)	481	1000	598	1000	9.8%	-0.12 [-0.16, -0.07]
Jain 2021 (3)	16	1000	25	1000	17.4%	-0.01 [-0.02, 0.00]
Kommalur 2021	18	58	38	75	1.3%	-0.20 [-0.36, -0.03]
Lu 2019	388	5786	473	7754	18.0%	0.01 [-0.00, 0.01]
**Total (95% CI)**		**12849**		**16079**	**100.0%**	**-0.04 [-0.06, -0.02]**
Total events	1136		1470			

Heterogeneity: $Tau^2 = 0.00$; $Chi^2 = 104.90$, df = 8 ($P < 0.00001$); $I^2 = 92\%$
Test for overall effect: Z = 3.51 (P = 0.0004)

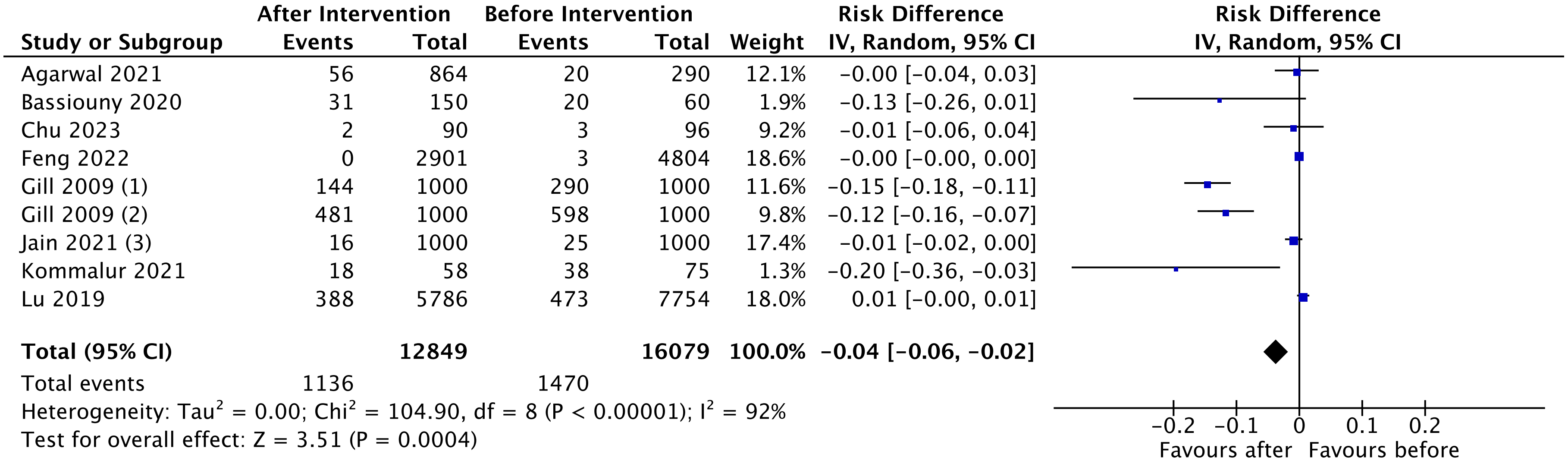

Footnotes
(1) Gill 2009 reported neonatal mortality in NICU 1 as deaths per 1000 admissions
(2) Gill 2009 reported neonatal mortality in NICU 2 as deaths per 1000 admissions
(3) Jain 2021 reported neonatal mortality per 1000 live births

Outcome: All-cause neonatal mortality (sensitivity analysis omitting high risk of bias studies)

	After Intervention		Before Intervention			Risk Difference
**Study or Subgroup**	**Events**	**Total**	**Events**	**Total**	**Weight**	**IV, Random, 95% CI**
Agarwal 2021	56	864	20	290	15.0%	−0.00 [−0.04, 0.03]
Bassiouny 2020	31	150	20	60	2.8%	−0.13 [−0.26, 0.01]
Chu 2023	2	90	3	96	11.9%	−0.01 [−0.06, 0.04]
Feng 2022	0	2901	3	4804	20.9%	−0.00 [−0.00, 0.00]
Gill 2009 (1)	144	1000	290	1000	14.5%	−0.15 [−0.18, −0.11]
Gill 2009 (2)	481	1000	598	1000	12.6%	−0.12 [−0.16, −0.07]
Kommalur 2021	18	58	38	75	2.0%	−0.20 [−0.36, −0.03]
Lu 2019	388	5786	473	7754	20.4%	0.01 [−0.00, 0.01]
**Total (95% CI)**		**11849**		**15079**	**100.0%**	**−0.04 [−0.07, −0.02]**
Total events	1120		1445			

Heterogeneity: $Tau^2 = 0.00$; $Chi^2 = 103.20$, df = 7 ($P < 0.00001$); $I^2 = 93\%$
Test for overall effect: Z = 3.53 (P = 0.0004)

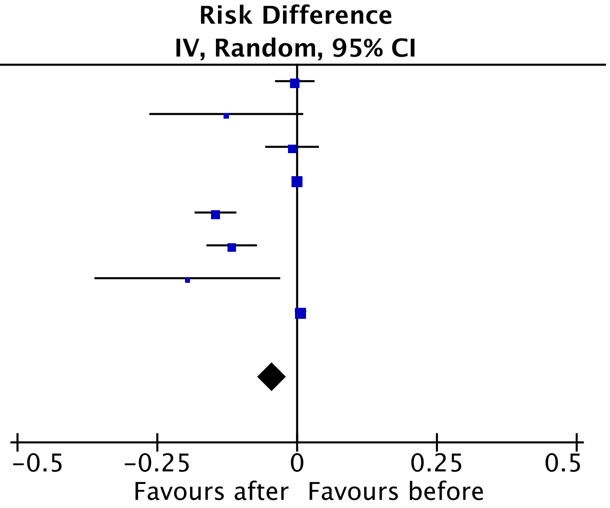

<u>Footnotes</u>
(1) Gill 2009 reported neonatal mortality in NICU 1 as deaths per 1000 admissions
(2) Gill 2009 reported neonatal mortality in NICU 2 as deaths per 1000 admissions

Outcome: Necrotizing enterocolitis (any Bell stage)

Study or Subgroup	After Intervention Events	After Intervention Total	Before Intervention Events	Before Intervention Total	Weight	Risk Difference IV, Random, 95% CI
Agarwal 2021 (1)	49	864	21	290	2.5%	−0.02 [−0.05, 0.02]
Chu 2023 (2)	8	90	7	96	0.5%	0.02 [−0.06, 0.09]
Feng 2022 (3)	30	2901	54	4804	57.5%	−0.00 [−0.01, 0.00]
Lu 2019 (4)	249	5786	287	7754	39.5%	0.01 [−0.00, 0.01]
**Total (95% CI)**		**9641**		**12944**	**100.0%**	**0.00 [−0.00, 0.01]**
Total events	336		369			

Heterogeneity: $Tau^2 = 0.00$; $Chi^2 = 3.85$, df = 3 (P = 0.28); $I^2 = 22\%$

Test for overall effect: Z = 0.57 (P = 0.57)

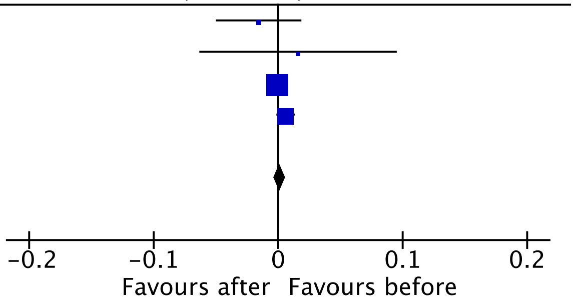

Footnotes

(1) Agarwal 2021 reported overall NEC

(2) Chu 2023 reported NEC (Bell stage ≥ II)

(3) Feng 2022 reported NEC (Bell stage ≥ II)

(4) Lu 2019 reported NEC (Stage ≥ II)

Outcome: Necrotizing enterocolitis (Bell stage ≥ II)

Study or Subgroup	After Intervention Events	After Intervention Total	Before Intervention Events	Before Intervention Total	Weight	Risk Difference IV, Random, 95% CI
Chu 2023	8	90	7	96	0.5%	0.02 [−0.06, 0.09]
Feng 2022	30	2901	54	4804	58.7%	−0.00 [−0.01, 0.00]
Lu 2019	249	5786	287	7754	40.8%	0.01 [−0.00, 0.01]
**Total (95% CI)**		**8777**		**12654**	**100.0%**	**0.00 [−0.00, 0.01]**
Total events	287		348			

Heterogeneity: $Tau^2 = 0.00$; $Chi^2 = 2.86$, df = 2 (P = 0.24); $I^2 = 30\%$

Test for overall effect: Z = 0.72 (P = 0.47)

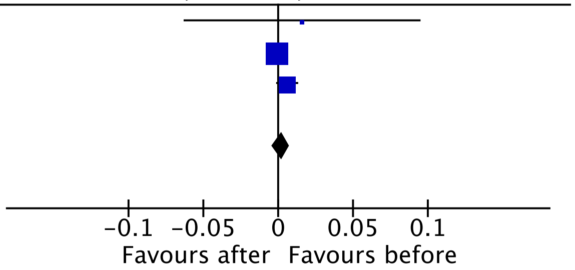

Outcome: Neonatal sepsis (any)

Study or Subgroup	After Intervention Events	After Intervention Total	Before Intervention Events	Before Intervention Total	Weight	Risk Difference IV, Random, 95% CI
Chu 2023 (1)	10	90	23	96	15.1%	−0.13 [−0.24, −0.02]
Feng 2022 (2)	10	2901	12	4804	32.9%	0.00 [−0.00, 0.00]
Lu 2019 (3)	376	5786	884	7754	32.6%	−0.05 [−0.06, −0.04]
Maalouf 2023 (4)	39	532	77	153	19.4%	−0.43 [−0.51, −0.35]
**Total (95% CI)**		**9309**		**12807**	**100.0%**	**−0.12 [−0.18, −0.06]**
Total events	435		996			

Heterogeneity: $Tau^2 = 0.00$; $Chi^2 = 207.91$, df = 3 ($P < 0.00001$); $I^2 = 99\%$
Test for overall effect: Z = 4.11 ($P < 0.0001$)

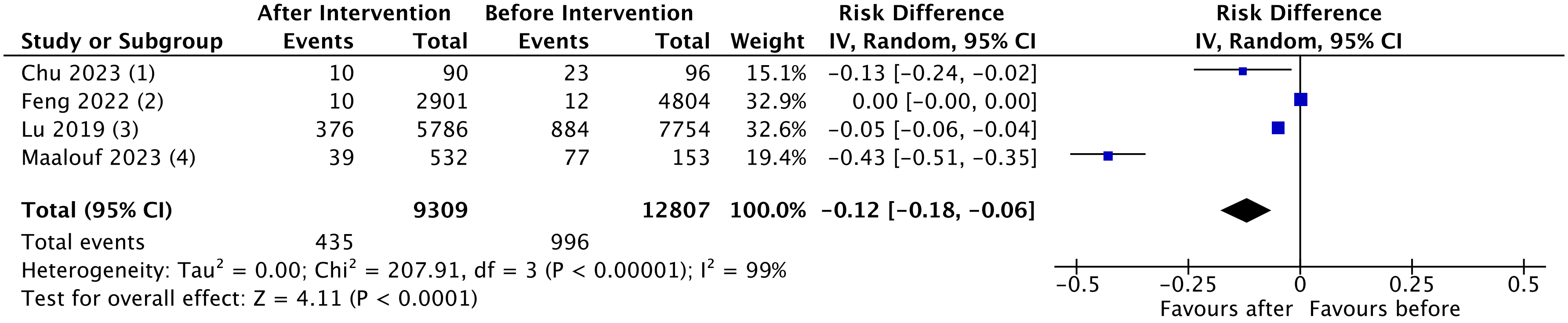

Footnotes
(1) A positive blood or CSF fluid culture, or clinical deterioration and ≥2 abnormal blood indicators or changes in CSF consistent with meningitis
(2) LOS defined as >72 hours of age and positive pathogenic results in blood, urine, or CSF fluid specimens
(3) Late-onset defined as ≥72 hours after birth
(4) EOS defined as ≤72 hours after birth

Outcome: Late-onset sepsis (>72 hours after birth)

Study or Subgroup	After Intervention Events	After Intervention Total	Before Intervention Events	Before Intervention Total	Weight	Risk Difference IV, Random, 95% CI
Chu 2023	10	90	23	96	13.1%	−0.13 [−0.24, −0.02]
Feng 2022 (1)	10	2901	12	4804	43.8%	0.00 [−0.00, 0.00]
Lu 2019 (2)	376	5786	884	7754	43.1%	−0.05 [−0.06, −0.04]
**Total (95% CI)**		**8777**		**12654**	**100.0%**	**−0.04 [−0.08, 0.01]**
Total events	396		919			

Heterogeneity: $Tau^2 = 0.00$; $Chi^2 = 104.27$, df = 2 ($P < 0.00001$); $I^2 = 98\%$
Test for overall effect: Z = 1.59 (P = 0.11)

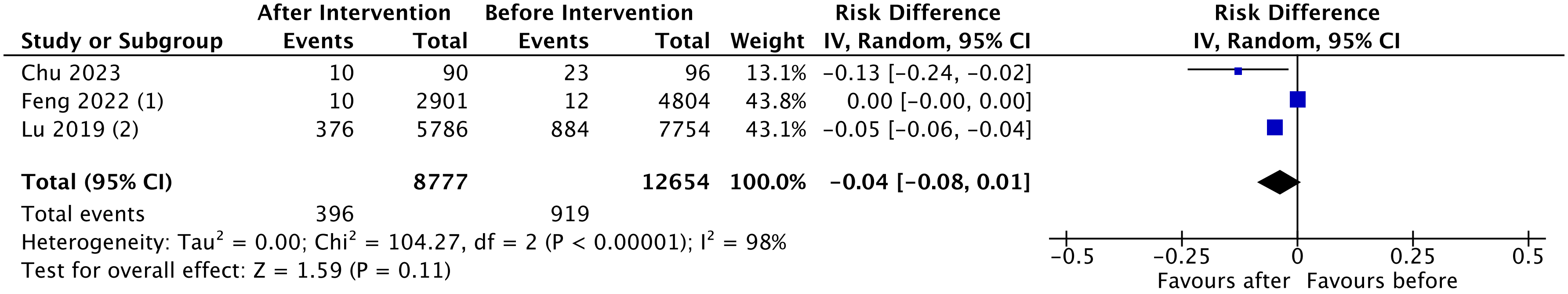

Footnotes
(1) Feng 2022 reported LOS defined as >72 hours of age and positive pathogenic results in blood, urine, or cerebrospinal fluid specimens
(2) Lu 2019 reported LOS and defined late-onset as ≥72 hours after birth

Outcome: Culture-negative sepsis

Study or Subgroup	After Intervention Events	After Intervention Total	Before Intervention Events	Before Intervention Total	Weight	Risk Difference IV, Random, 95% CI
Feng 2022 (1)	368	2901	663	4804	11.9%	−0.01 [−0.03, 0.00]
Lu 2019 (2)	156	5786	248	7754	88.1%	−0.01 [−0.01, 0.00]
**Total (95% CI)**		**8687**		**12558**	**100.0%**	**-0.01 [-0.01, -0.00]**
Total events	524		911			

Heterogeneity: $Tau^2 = 0.00$; $Chi^2 = 0.53$, df = 1 (P = 0.47); $I^2 = 0\%$
Test for overall effect: Z = 2.10 (P = 0.04)

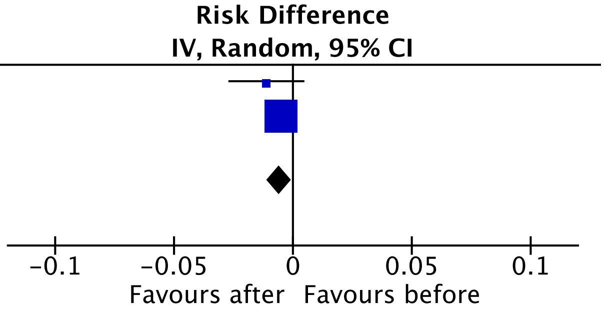

Footnotes
(1) Feng 2022 reported infants treated in ≤5 days for culture−negative sepsis
(2) Lu 2019 reported all infants treated for culture−negative sepsis

Outcome: Pneumonia

Study or Subgroup	After Intervention Events	After Intervention Total	Before Intervention Events	Before Intervention Total	Weight	Risk Difference IV, Random, 95% CI
Feng 2022 (1)	128	2901	296	4804	49.4%	−0.02 [−0.03, −0.01]
Lu 2019 (2)	434	5786	543	7754	50.6%	0.00 [−0.00, 0.01]
**Total (95% CI)**		**8687**		**12558**	**100.0%**	**-0.01 [-0.03, 0.02]**
Total events	562		839			

Heterogeneity: $Tau^2 = 0.00$; $Chi^2 = 10.75$, df = 1 (P = 0.001); $I^2 = 91\%$
Test for overall effect: Z = 0.54 (P = 0.59)

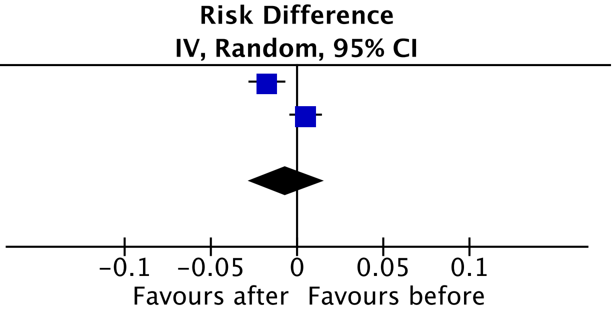

Footnotes
(1) Feng 2022 reported infants treated in ≤5 days for pneumonia
(2) Lu 2019 reported all infants treated for pneumonia

Outcome: Multidrug-resistant organism infections or colonizations

	After Intervention		Before Intervention			Risk Difference
**Study or Subgroup**	**Events**	**Total**	**Events**	**Total**	**Weight**	**IV, Random, 95% CI**
Feng 2022 (1)	3	2901	7	4804	60.9%	−0.00 [−0.00, 0.00]
Lu 2019 (2)	58	5786	109	7754	39.1%	−0.00 [−0.01, −0.00]
**Total (95% CI)**		**8687**		**12558**	**100.0%**	**−0.00 [−0.01, 0.00]**
Total events	61		116			

Heterogeneity: $Tau^2 = 0.00$; $Chi^2 = 3.13$, df = 1 (P = 0.08); $I^2 = 68\%$
Test for overall effect: Z = 1.04 (P = 0.30)

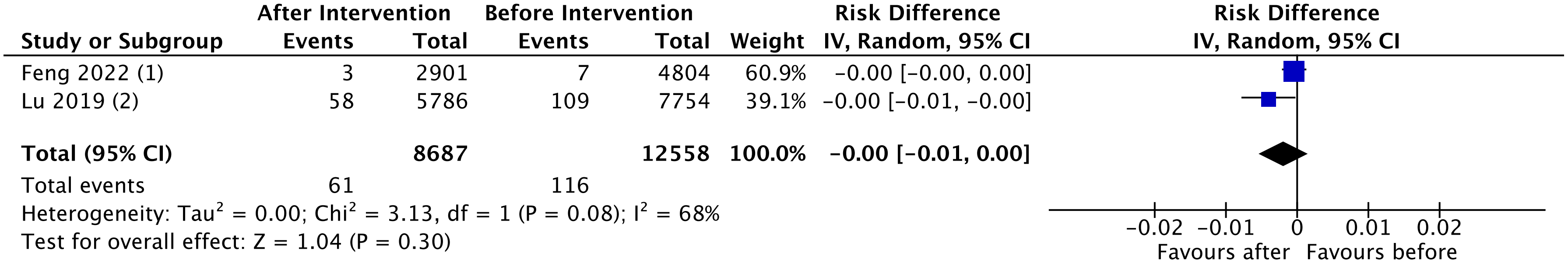

Footnotes
(1) Feng 2022 reported multidrug−resistant organism infections
(2) Lu 2019 reported multidrug−resistant organism colonizations

Outcome: Bloodstream isolates of methicillin-resistant *Staphylococcus aureus* (MRSA)

	After Intervention		Before Intervention			Risk Difference
**Study or Subgroup**	**Events**	**Total**	**Events**	**Total**	**Weight**	**IV, Random, 95% CI**
Bassiouny 2020	4	58	0	20	0.2%	0.07 [−0.03, 0.16]
Gill 2009	1	776	0	486	98.8%	0.00 [−0.00, 0.01]
Kommalur 2021	1	58	1	75	1.0%	0.00 [−0.04, 0.05]
**Total (95% CI)**		**892**		**581**	**100.0%**	**0.00 [−0.00, 0.01]**
Total events	6		1			

Heterogeneity: $Tau^2 = 0.00$; $Chi^2 = 2.00$, df = 2 (P = 0.37); $I^2 = 0\%$
Test for overall effect: Z = 0.68 (P = 0.50)

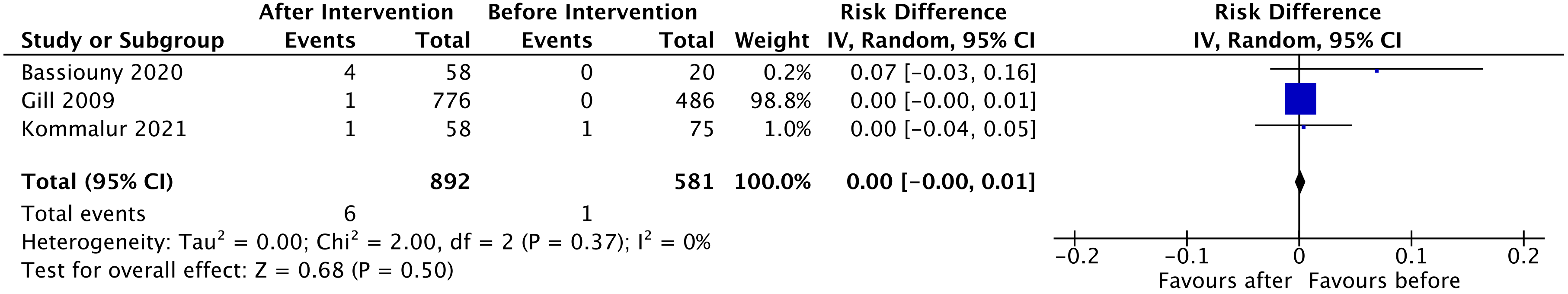

Outcome: Bloodstream isolates of *Klebsiella* spp.

Study or Subgroup	After Intervention Events	After Intervention Total	Before Intervention Events	Before Intervention Total	Weight	Risk Difference IV, Random, 95% CI
Bassiouny 2020	12	58	8	20	2.2%	−0.19 [−0.43, 0.05]
Gill 2009	87	776	76	486	81.3%	−0.04 [−0.08, −0.01]
Kommalur 2021 (1)	3	58	7	75	16.5%	−0.04 [−0.13, 0.05]
**Total (95% CI)**		**892**		**581**	**100.0%**	**−0.05 [−0.08, −0.01]**
Total events	102		91			

Heterogeneity: $Tau^2 = 0.00$; $Chi^2 = 1.47$, df = 2 (P = 0.48); $I^2 = 0\%$
Test for overall effect: Z = 2.61 (P = 0.009)

Risk Difference
IV, Random, 95% CI

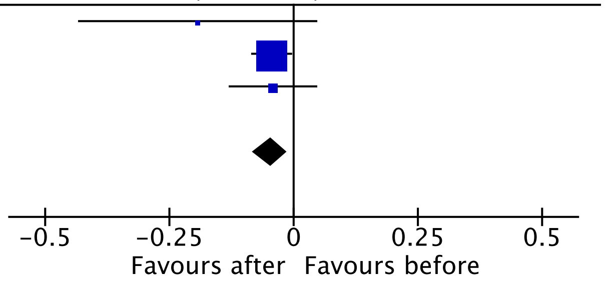

Footnotes
(1) Kommalur 2021 reported *Klebsiella pneumoniae* organisms

Outcome: Bloodstream isolates of *Acinetobacter* spp.

Study or Subgroup	After Intervention Events	After Intervention Total	Before Intervention Events	Before Intervention Total	Weight	Risk Difference IV, Random, 95% CI
Bassiouny 2020	8	58	1	20	6.7%	0.09 [−0.04, 0.22]
Gill 2009 (1)	5	776	3	486	70.1%	0.00 [−0.01, 0.01]
Kommalur 2021 (2)	1	58	4	75	23.2%	−0.04 [−0.10, 0.02]
**Total (95% CI)**		**892**		**581**	**100.0%**	**−0.00 [−0.04, 0.03]**
Total events	14		8			

Heterogeneity: $Tau^2 = 0.00$; $Chi^2 = 3.10$, df = 2 (P = 0.21); $I^2 = 35\%$
Test for overall effect: Z = 0.13 (P = 0.90)

Risk Difference
IV, Random, 95% CI

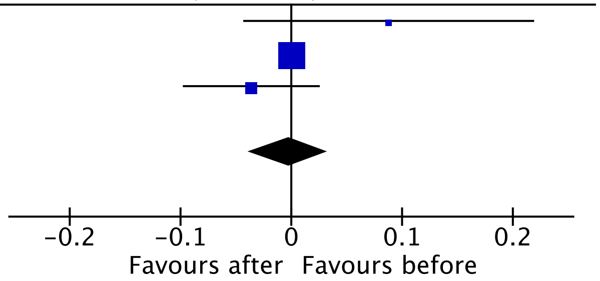

Footnotes
(1) Gill 2009 reported *Acinetobacter baumanii* organisms
(2) Kommalur 2021 reported *Acinetobacter baumanii* organisms

Outcome: Bloodstream isolates of *Escherichia coli* (*E. coli*)

	After Intervention		Before Intervention			Risk Difference
Study or Subgroup	Events	Total	Events	Total	Weight	IV, Random, 95% CI
Bassiouny 2020	4	58	0	20	15.5%	0.07 [−0.03, 0.16]
Gill 2009	8	776	16	486	49.7%	−0.02 [−0.04, −0.01]
Kommalur 2021	1	58	0	75	34.9%	0.02 [−0.03, 0.06]
**Total (95% CI)**		**892**		**581**	**100.0%**	**0.01 [−0.04, 0.05]**
Total events	13		16			

Heterogeneity: $Tau^2 = 0.00; Chi^2 = 5.85, df = 2 (P = 0.05); I^2 = 66\%$
Test for overall effect: Z = 0.24 (P = 0.81)

Risk Difference
IV, Random, 95% CI

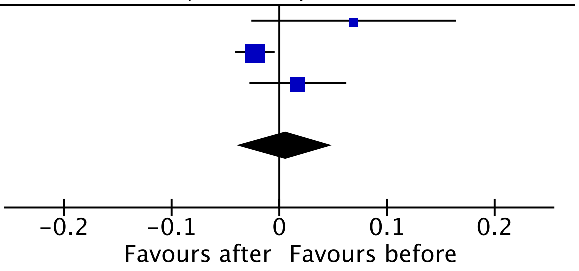

Outcome: Bloodstream isolates of *Enterobacter* spp.

	After Intervention		Before Intervention			Risk Difference
Study or Subgroup	Events	Total	Events	Total	Weight	IV, Random, 95% CI
Bassiouny 2020	4	58	0	20	13.9%	0.07 [−0.03, 0.16]
Gill 2009	48	776	47	486	42.5%	−0.03 [−0.07, −0.00]
Kommalur 2021	0	58	0	75	43.6%	0.00 [−0.03, 0.03]
**Total (95% CI)**		**892**		**581**	**100.0%**	**−0.01 [−0.05, 0.04]**
Total events	52		47			

Heterogeneity: $Tau^2 = 0.00; Chi^2 = 5.53, df = 2 (P = 0.06); I^2 = 64\%$
Test for overall effect: Z = 0.25 (P = 0.80)

Risk Difference
IV, Random, 95% CI

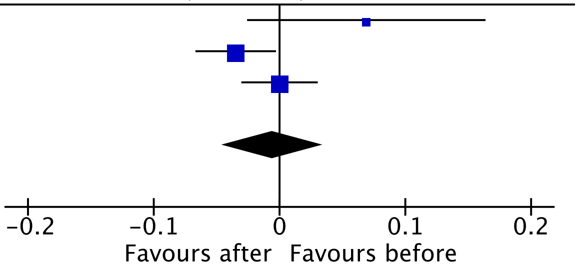

Outcome: Bloodstream isolates of coagulase-negative *staphylococci* (CoNS)

	After Intervention		Before Intervention			Risk Difference
Study or Subgroup	Events	Total	Events	Total	Weight	IV, Random, 95% CI
Bassiouny 2020	14	58	1	20	10.5%	0.19 [0.05, 0.34]
Gill 2009	34	776	11	486	48.8%	0.02 [0.00, 0.04]
Kommalur 2021	0	58	1	75	40.7%	−0.01 [−0.05, 0.03]
**Total (95% CI)**		**892**		**581**	**100.0%**	**0.02 [−0.03, 0.08]**
Total events	48		13			

Heterogeneity: $Tau^2 = 0.00; Chi^2 = 7.99, df = 2 (P = 0.02); I^2 = 75\%$
Test for overall effect: Z = 0.93 (P = 0.35)

Risk Difference
IV, Random, 95% CI

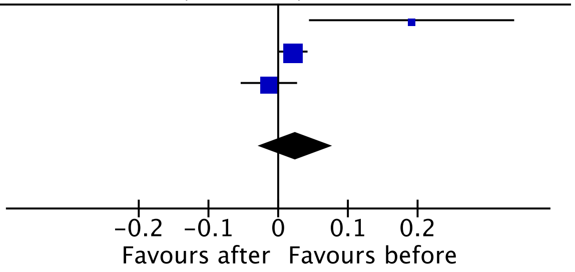

Outcome: Bloodstream isolates of *Pseudomonas* spp.

Study or Subgroup	After Intervention Events	After Intervention Total	Before Intervention Events	Before Intervention Total	Weight	Risk Difference IV, Random, 95% CI
Bassiouny 2020	1	58	0	20	24.9%	0.02 [−0.06, 0.09]
Gill 2009	20	776	52	486	38.9%	−0.08 [−0.11, −0.05]
Kommalur 2021 (1)	0	58	1	75	36.2%	−0.01 [−0.05, 0.03]
**Total (95% CI)**		**892**		**581**	**100.0%**	**−0.03 [−0.09, 0.03]**
Total events	21		53			

Heterogeneity: $Tau^2 = 0.00$; $Chi^2 = 10.61$, df = 2 (P = 0.005); $I^2 = 81\%$

Test for overall effect: Z = 1.07 (P = 0.29)

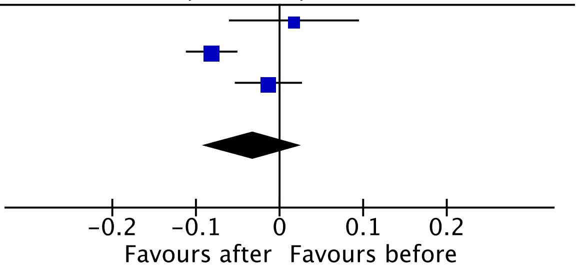

Footnotes

(1) Kommalur 2021 reported *Pseudomonas aeruginosa* organisms

Outcome: Bloodstream isolates of *Candida* spp.

Study or Subgroup	After Intervention Events	After Intervention Total	Before Intervention Events	Before Intervention Total	Weight	Risk Difference IV, Random, 95% CI
Bassiouny 2020	3	58	8	20	44.5%	−0.35 [−0.57, −0.13]
Kommalur 2021	0	58	1	75	55.5%	−0.01 [−0.05, 0.03]
**Total (95% CI)**		**116**		**95**	**100.0%**	**−0.16 [−0.49, 0.16]**
Total events	3		9			

Heterogeneity: $Tau^2 = 0.05$; $Chi^2 = 8.47$, df = 1 (P = 0.004); $I^2 = 88\%$

Test for overall effect: Z = 0.97 (P = 0.33)

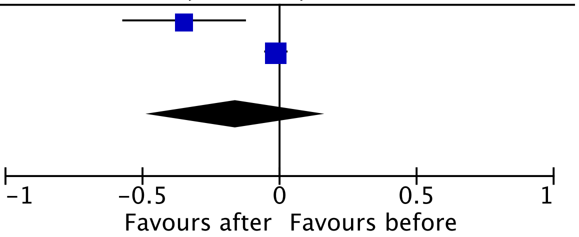

Outcome: Number of newborns receiving antibiotics

Study or Subgroup	After Intervention Events	After Intervention Total	Before Intervention Events	Before Intervention Total	Weight	Risk Difference IV, Random, 95% CI
Agarwal 2021 (1)	544	864	226	290	22.2%	−0.15 [−0.21, −0.09]
Chu 2023 (2)	60	90	95	96	18.3%	−0.32 [−0.42, −0.22]
Feng 2022 (3)	1341	2901	3153	4804	24.5%	−0.19 [−0.22, −0.17]
Kommalur 2021 (4)	44	58	63	75	14.7%	−0.08 [−0.22, 0.06]
Maalouf 2023 (5)	208	532	118	153	20.3%	−0.38 [−0.46, −0.30]
**Total (95% CI)**		**4445**		**5418**	**100.0%**	**−0.23 [−0.31, −0.15]**
Total events	2197		3655			

Heterogeneity: $Tau^2 = 0.01$; $Chi^2 = 32.09$, df = 4 (P < 0.00001); $I^2 = 88\%$
Test for overall effect: Z = 5.46 (P < 0.00001)

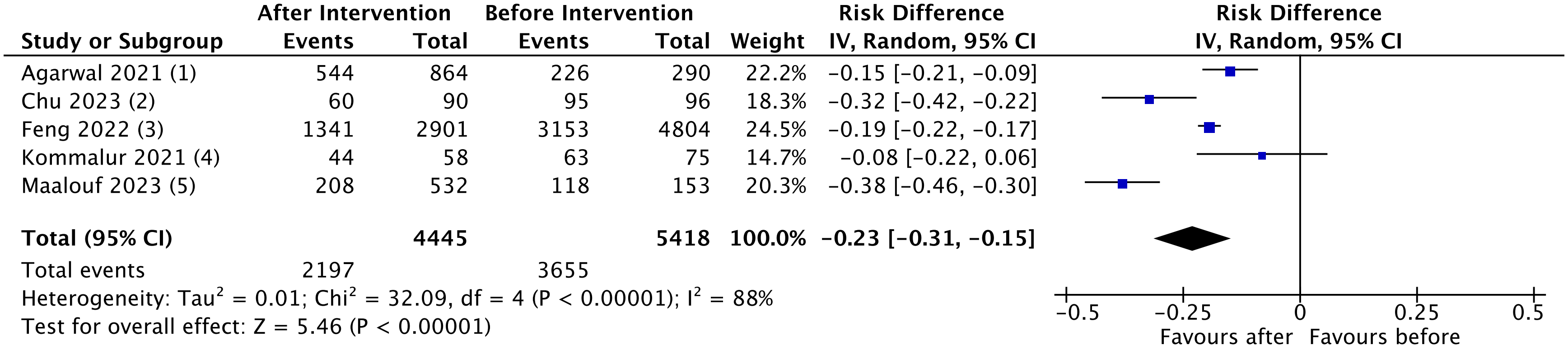

Footnotes
(1) Agarwal 2021 reported neonates unexposed to antibiotics (from which the neonates exposed to antibiotics was derived)
(2) Chu 2023 reported proportion of early antibiotic usage
(3) Feng 2022 reported proportion of antibiotic exposure
(4) Kommalur 2021 reported percentage of neonates with no antibiotics (from which neonates receiving antibiotics was derived)
(5) Maalouf 2023 reported proportion of neonates treated for early-onset sepsis

Outcome: Duration of antibiotic therapy >5 days

Study or Subgroup	After Intervention Events	After Intervention Total	Before Intervention Events	Before Intervention Total	Weight	Risk Difference IV, Random, 95% CI
Chu 2023 (1)	19	90	92	96	49.7%	−0.75 [−0.84, −0.65]
Feng 2022 (2)	654	2901	1882	4804	50.3%	−0.17 [−0.19, −0.15]
**Total (95% CI)**		**2991**		**4900**	**100.0%**	**−0.45 [−1.02, 0.11]**
Total events	673		1974			

Heterogeneity: $Tau^2 = 0.17$; $Chi^2 = 142.01$, df = 1 (P < 0.00001); $I^2 = 99\%$
Test for overall effect: Z = 1.57 (P = 0.12)

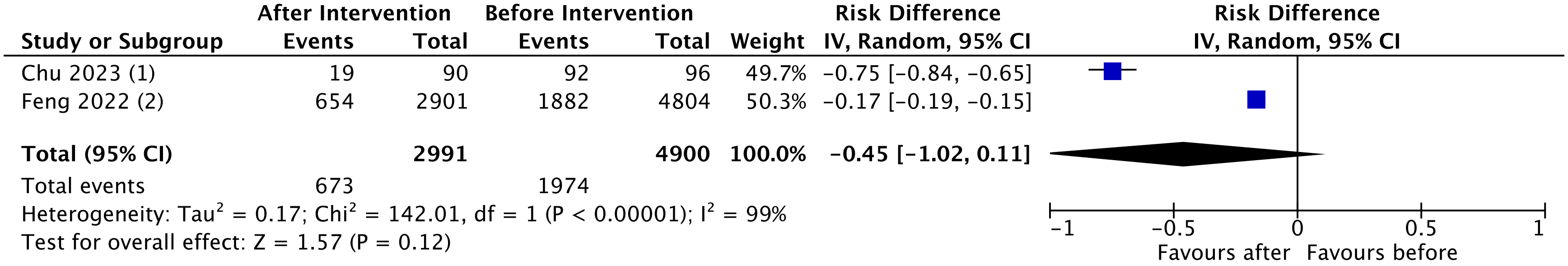

Footnotes
(1) Chu 2023 reported the proportion of neonates treated with an initial antibiotic course >7 days
(2) Feng 2022 reported the proportion of neonates treated with a duration of therapy >5 days

Outcome: Neonates with antibiotics discontinued after 48 hours

	After Intervention		Before Intervention			Risk Difference
**Study or Subgroup**	**Events**	**Total**	**Events**	**Total**	**Weight**	**IV, Random, 95% CI**
Kommalur 2021	11	58	0	75	49.3%	0.19 [0.09, 0.29]
Lu 2019	5497	5786	2481	7754	50.7%	0.63 [0.62, 0.64]
**Total (95% CI)**		**5844**		**7829**	**100.0%**	**0.41 [−0.02, 0.84]**
Total events	5508		2481			

Heterogeneity: $Tau^2 = 0.10$; $Chi^2 = 69.73$, df = 1 ($P < 0.00001$); $I^2 = 99\%$

Test for overall effect: Z = 1.88 (P = 0.06)

Risk Difference
IV, Random, 95% CI

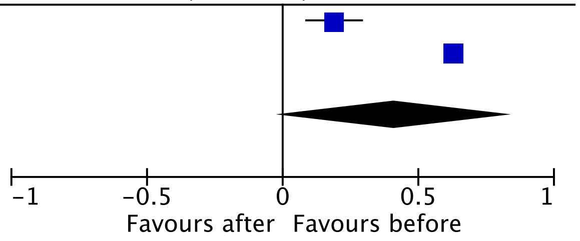

Appendix N: Effect direction plot of secondary outcomes

Study	Study design	Sample size*	Overall antibiotic usage	Access antibiotic usage	Watch antibiotic usage	Reserve antibiotic usage	Restricted antibiotic usage	Duration of antibiotic usage	Length of hospital stay
Chimhini 2020	QE	189	▲	▲	▲			▲	▲
Ren 2023	QE	8,706	▲		▲				▲
Agarwal 2021	QE	1,138	▲		▲			▲	
Akintan 2024	Obs	19	▼						
Alp 2015	QE	148							▼
Bassiouny 2020	QE	150	◄►	▼	◄►	◄►			▲
Chu 2023	QE	90	▲						▼
Dramowski 2024	QE	565						▲	
El-Baky 2020	QE	617							◄►
Feng 2022	QE	2,901	▲	▲	▼			◄►	▼
Gill 2009	QE	902							
Graus 2022[a]	QE	NR	▲						
Huang 2019	QE	5,972							
Jinka 2017	QE	1,276	▲	▼	◄►	▼			▲
Kommalur 2021	QE	58	▲		▲				
Landre-Peigne 2011	QE	148	▲						
Lu 2019	QE	5,786	▲						◄►
Maalouf 2023	QE	563	▲	▲	▲		▲		▼
Olita'a 2019	Obs	85							
Sathyan 2022	QE	43	▲						
Sowjanya 2018	QE	428	▲	▲	▲		▲	▲	
Wang 2020	QE	4,488	▲						▼
Xiao-Lu 2016	QE	7,265	▲	▲	▲			▼	▼
Yin 2020	QE	6,516							
Zain 2021	RCT	60							
Farias-Filho 2024	QE	915	▲		▲				
Garpvall 2021	QE	323							
Jain 2021[b]	QE	NR	▲					▲	
Konda 2021[c]	QE	NR	▲						
Nygren 2025	QE	250							
Sewornu 2025	QE	249							

Study design: Obs, observational study; QE, quasi-experimental study; RCT, randomised controlled trial
**Sample size represents the number of individuals in the intervention/exposed group; NR, not reported*

Effect direction: upward arrow ▲= positive health impact (improvement), downward arrow ▼= negative health impact (deterioration), sideways arrow ◄►= no change/mixed effects/conflicting findings
Study quality: denoted by row colour: green = low risk of bias; amber = some concerns; red = high risk of bias
[a]Graus 2022 reported the total number of newborns admitted to the NICU as N=858 but did not report the sample sizes of the pre-intervention and post-intervention groups; therefore, study findings should be interpreted with caution
[b]Jain 2021 did not report total or intervention group sample sizes; therefore, study findings should be interpreted with caution
[c]Konda 2021 reported the baseline sample size as N=200, but did not report the intervention group sample size nor the overall study population size with which to calculate the intervention group sample size; thus, study findings should be interpreted with caution
***Figure 2:* Effect direction plot summarizing the impact of strategies to reduce AMR on secondary outcomes of antibiotic usage and length of hospital stay**

References

1 Fitchett EJA, Seale AC, Vergnano S, Sharland M, Heath PT, Saha SK, et al. Strengthening the Reporting of Observational Studies in Epidemiology for Newborn Infection (STROBE-NI): an extension of the STROBE statement for neonatal infection research. Lancet Infect Dis. 2016;16(10):e202–e13.

2 Araujo da Silva AR, Jaszkowski E, Schober T, von Both U, Meyer-Buehn M, Marques AF, et al. Patterns of antimicrobial consumption in neonatal and pediatric intensive care units in Germany and Brazil. Eur J Clin Microbiol Infect Dis. 2020;39(2):249–55.

3 Azab SF, Sherbiny HS, Saleh SH, Elsaeed WF, Elshafiey MM, Siam AG, et al. Reducing ventilator-associated pneumonia in neonatal intensive care unit using "VAP prevention Bundle": a cohort study. BMC Infect Dis. 2015;15:314.

4 Balla KC, Rao SPN, Arul C, Shashidhar A, Prashantha YN, Nagaraj S, et al. Decreasing Central Line-associated Bloodstream Infections Through Quality Improvement Initiative. Indian Pediatrics. 2018;55.

5 Bazan IGM, Lobo BBP, Schreiber AZ, Calil R, Marba STM, de Siqueira Caldas JP. Long-lasting effects of control measures on trends in incidence in neonatal late-onset sepsis due to multiresistant bacteria in a Brazilian neonatal unit. Am J Infect Control. 2025;53(1):22–9.

6 Dramowski A, Aucamp M, Bekker A, Pillay S, Moloto K, Whitelaw AC, et al. NeoCLEAN: a multimodal strategy to enhance environmental cleaning in a resource-limited neonatal unit. Antimicrob Resist Infect Control. 2021;10(1):35.

7 Hussain AS, Ahmed AM, Arbab S, Ariff S, Ali R, Demas S, et al. CLABSI reduction using evidence based interventions and nurse empowerment: a quality improvement initiative from a tertiary care NICU in Pakistan. Arch Dis Child. 2021;106(4):394–400.

8 Iqbal F, Siva N, Lewis LES, Purkayastha J, Bharadwaj SK, Nayak BS, et al. Assessment of an Antimicrobial Stewardship Program for Enhancing Clinical Knowledge in Neonatal Care Settings With High Antimicrobial Resistance. Quality Management in Health Care. 2025;34(3):220–7.

9 Johnson J, Latif A, Randive B, Kadam A, Rajput U, Kinikar A, et al. Implementation of the Comprehensive Unit-Based Safety Program to Improve Infection Prevention and Control Practices in Four Neonatal Intensive Care Units in Pune, India. Front Pediatr. 2021;9:794637.

10 Khan N, Chimhini G, Shrestha SK, Cortina-Borja M, Chimhuya S, Zailani G, et al. Assessing the Use of Neonatal Sepsis Guidelines and Antibiotic Prescription With Large-Scale Prospective Data From Zimbabwe and Malawi Open Access. Journal of the Pediatric Infectious Diseases Society. 2025;14(4):1–9.

11 Kulali F, Calkavur S, Oruc Y, Demiray N, Devrim I. Impact of central line bundle for prevention of umbilical catheter-related bloodstream infections in a neonatal intensive care unit: A pre-post intervention study. Am J Infect Control. 2019;47(4):387–90.

12 Lloyd LG, van Weissenbruch MM, Bekker A, Ferreyra C, Gleeson B, Dramowski A. Theoretical impact of a bedside decision-making tool on antibiotic use for suspected neonatal healthcare-associated infection: an observational study. BMC Pediatr. 2025;25(1) (no pagination).

13 Mwananyanda L, Pierre C, Mwansa J, Cowden C, Localio AR, Kapasa ML, et al. Preventing Bloodstream Infections and Death in Zambian Neonates: Impact of a Low-cost Infection Control Bundle. Clin Infect Dis. 2019;69(8):1360–7.

14 Navoa-Ng JA, Berba R, Galapia YA, Rosenthal VD, Villanueva VD, Tolentino MC, et al. Device-associated infections rates in adult, pediatric, and neonatal intensive care units of hospitals in the Philippines: International Nosocomial Infection Control Consortium (INICC) findings. Am J Infect Control. 2011;39(7):548–54.

15 Picheansathian W, Pearson A, Suchaxaya P. The effectiveness of a promotion programme on hand hygiene compliance and nosocomial infections in a neonatal intensive care unit. Int J Nurs Pract. 2008;14(4):315–21.

16 Rahim RH, Barnett T. Reducing nosocomial infection in neonatal intensive care: an intervention study. Int J Nurs Pract. 2009;15(6):580–4.

17 Resende DS, Moreira do O J, von Dolinger de Brito D, Abdullah VOS, Filho PPG. Reduction of catheter-associated bloodstream infections through procedures in newborn babies admitted in a university hospital intensive care unit in Brazil. Revista da Sociedade Brasileira de Medicina Tropical. 2011;44(6):731–4.

18 Rosenthal VD, Rodriguez-Calderon ME, Rodriguez-Ferrer M, Singhal T, Pawar M, Sobreyra-Oropeza M, et al. Findings of the International Nosocomial Infection Control Consortium (INICC), Part II: Impact of a multidimensional strategy to reduce ventilator-associated pneumonia in neonatal intensive care units in 10 developing countries. Infect Control Hosp Epidemiol. 2012;33(7):704–10.

19 Rosenthal VD, Duenas L, Sobreyra-Oropeza M, Ammar K, Navoa-Ng JA, de Casares AC, et al. Findings of the International Nosocomial Infection Control Consortium (INICC), part III: effectiveness of a multidimensional infection control approach to reduce central line-associated bloodstream infections in the neonatal intensive care units of 4 developing countries. Infect Control Hosp Epidemiol. 2013;34(3):229–37.

20 Tagare A, Kadam S, Vaidya U, Pandit A. Routine antibiotic use in preterm neonates: a randomised controlled trial. Journal of Hospital Infection. 2009;74:332–6.

21 Tran HT, Mannava P, Murray JCS, Nguyen PTT, Tuyen LTM, Hoang Anh T, et al. Early Essential Newborn Care Is Associated With Reduced Adverse Neonatal Outcomes in a Tertiary Hospital in Da Nang, Viet Nam: A Pre- Post- Intervention Study. EClinicalMedicine. 2018;6:51–8.

22 Urzúa S FM, García P, Sánchez A, Luco M. Estrategias para reducir infecciones, uso de antimicrobianos y sus efectos en una unidad de neonatología [Strategies to reduce infections and antibiotic use and its effects in a neonatal care unit]. Rev Chilena Infectol. 2017;Apr;34(2):99-107.

23 Wang W, Zhao C, Ji Q, Liu Y, Shen G, Wei L. Prevention of peripherally inserted central line-associated blood stream infections in very low-birth-weight infants by using a central line bundle guideline with a standard checklist: a case control study. BMC Pediatr. 2015;15:69.

24 WHO. Web Annex B. World Health Organization Model List of Essential Medicines for Children - 9th List, 2023. 2023. In: The selection and use of essential medicines 2023: Executive summary of the report of the 24th WHO Expert Committee on the Selection and Use of Essential Medicines, 24-28 April 2023 [Internet]. Geneva: World Health Organization. Available from: https://iris.who.int/bitstream/handle/10665/371091/WHO-MHP-HPS-EML-2023.03-eng.pdf?sequence=1.

25 WHO. Antimicrobial stewardship programmes in health-care facilities in low- and middle-income countries. A practical toolkit. Geneva: World Health Organization; 2019.

26 Agarwal S, Patodia J, Mittal J, Singh Y, Agnihotri V, Sharma V. Antibiotic stewardship in a tertiary care NICU of northern India: a quality improvement initiative. BMJ Open Qual. 2021;10(Suppl 1).

27 Akintan P, Oshun P, Osuagwu C, Ola-Bello O, Fajolu I, Roberts A, et al. Point prevalence surveys of antibiotic prescribing in children at a tertiary hospital in a resource constraint, low-income sub-Saharan African country-the impact of an antimicrobial stewardship program. BMC Pediatr. 2024;24(1):383.

28 Alp E, Orhan T, Kurkcu CA, Ersoy S, McLaws ML. The first six years of surveillance in pediatric and neonatal intensive care units in Turkey. Antimicrob Resist Infect Control. 2015;4:34.

29 Bassiouny DM, Hassan RM, Shalaby A, Halim MMA, Wassef MA. Establishment of an antimicrobial stewardship strategy on the surgical NICU at Cairo University specialized pediatric hospital. J Pediatr Surg. 2020;55(9):1959–64.

30 Chimhini G, Chimhuya S, Madzudzo L, Heys M, Crehan C, Robertson V, et al. Auditing use of antibiotics in Zimbabwean neonates. Infect Prev Pract. 2020;2(2):100046.

31 Chu M, Lin J, Wang M, Liao Z, Cao C, Hu M, et al. Restrictive Use of Empirical Antibiotics Is Associated with Improved Short Term Outcomes in Very Low Birth Weight Infants: A Single Center, Retrospective Cohort Study from China. Antibiotics (Basel). 2023;12(4).

32 Dramowski A, Prusakov P, Goff DA, Brink A, Govender NP, Annor AS, et al. Prospective antimicrobial stewardship interventions by multidisciplinary teams to reduce neonatal antibiotic use in South Africa: The Neonatal Antimicrobial Stewardship (NeoAMS) study. Int J Infect Dis. 2024;146:107158.

33 El-Baky RMA, Senosy EM, Omara W, Mohamed DS, Ibrahim RA. The Impact of the Implementation of Culture-based Antibiotic Policy on the Incidence of Nosocomial Infections in Neonates Hospitalized in Neonatal Intensive Care Unit in a General Egyptian Hospital in Upper Egypt, 2016-2018. Journal of Pure and Applied Microbiology. 2020;14(3):1879–92.

34 Farias-Filho FA, Souza PV, Nascimento SNdR, Rodrigues RdC, Nascimento MMGd, Carvalho VdF. Multiple-step antimicrobial stewardship approach in a neonatal intensive care unit: a quasi-experimental study. Rev epidemiol controle infecç. 2024;14(3):382–8.

35 Feng K, He Y, Liu W, Zhang X, Song P, Hua Z. Evaluation of antibiotic stewardship among near-term and term infants admitted to a neonatal unit. Eur J Pediatr. 2022;182(1):245–54.

36 Garpvall K, Duong V, Linnros S, Quoc TN, Mucchiano D, Modeen S, et al. Admission screening and cohort care decrease carbapenem resistant enterobacteriaceae in Vietnamese pediatric ICU's. Antimicrob Resist Infect Control. 2021;10(1):128.

37 Gill CJ, Mantaring JB, Macleod WB, Mendoza M, Mendoza S, Huskins WC, et al. Impact of enhanced infection control at 2 neonatal intensive care units in the Philippines. Clin Infect Dis. 2009;48(1):13–21.

38 Graus JM, Herbozo C, Hernandez R, Pantoja AF, Zegarra J. Managing antibiotics wisely in a neonatal intensive care unit in a low resource setting. J Perinatol. 2022;42(7):965–70.

39 Huang H, Ran J, Yang J, Li P, Zhuang G. Impact of MRSA Transmission and Infection in a Neonatal Intensive Care Unit in China: A Bundle Intervention Study during 2014-2017. Biomed Res Int. 2019;2019:5490413.

40 Jain M, Bang A, Meshram P, Gawande P, Kawhale K, Kamble P, et al. Institution of an antibiotic stewardship programme for rationalising antibiotic usage: a quality improvement project in the NICU of a public teaching hospital in rural central India. BMJ Open Qual. 2021;10(Suppl 1).

41 Jinka DR, Gandra S, Alvarez-Uria G, Torre N, Tadepalli D, RPR N. Impact of Antibiotic Policy on Antibiotic Consumption in a Neonatal Intensive Care Unit in India. Indian Pediatrics. 2017;54:739–41.

42 Kommalur A, Baddadka V, Devadas S, Kariyappa M, Dakshayani B, Krishnapura Lakshminarayana S, et al. Decreasing antibiotic over-use by implementation of an antibiotic stewardship programme in preterm neonates in resource limited settings - a quality improvement initiative. Paediatr Int Child Health. 2021;41(2):103–11.

43 Konda KC, Singh H, Madireddy A, Poodari MMR. Quality improvement initiative approach to decrease the unindicated usage of antibiotics in a neonatal intensive care unit of a tertiary care teaching hospital in Hyderabad, India. BMJ Open Qual. 2021;10(Suppl 1).

44 Landre-Peigne C, Ka AS, Peigne V, Bougere J, Seye MN, Imbert P. Efficacy of an infection control programme in reducing nosocomial bloodstream infections in a Senegalese neonatal unit. J Hosp Infect. 2011;79(2):161–5.

45 Lu C, Liu Q, Yuan H, Wang L. Implementation of the Smart Use of Antibiotics Program to Reduce Unnecessary Antibiotic Use in a Neonatal ICU: A Prospective Interrupted Time-Series Study in a Developing Country. Crit Care Med. 2019;47(1):e1–e7.

46 Maalouf FI, Saad T, Zakhour R, Yunis K. Successful establishment and five-year sustainability of a neonatal-specific antimicrobial stewardship program in a low middle-income country. Front Pharmacol. 2022;13:1076392.

47 Nygren D, Andersson F, Barrow E, Bittaye SO, Bah H, Banja F, et al. Bloodstream Infections and Antimicrobial Resistance in The Gambia: Continuous Surveillance from a Tertiary Care Center. The American journal of tropical medicine and hygiene. 2025;23.

48 Rahden P, Barrow E, Bah H, Bittaye SO, Nygren D, Badjan A. Bloodstream infections at a tertiary hospital in the Gambia - a one-year retrospective study. BMC Infect Dis. 2025;25(1) (no pagination).

49 Olita'a D, Barnabas R, Vali Boma G, Pameh W, Vince J, Duke T. Simplified management protocol for term neonates after prolonged rupture of membranes in a setting with high rates of neonatal sepsis and mortality: a quality improvement study. Arch Dis Child. 2019;104(2):115–20.

50 Ren Z, Yang S, Han J, Nie C, Wang C, Wang J, et al. Reduction of antibiotic use and multi-drug resistance bacteria infection in neonates after improvement of antibiotics use strategy in a level 4 neonatal intensive care unit in southern China. Eur J Clin Microbiol Infect Dis. 2023;42(1):87–98.

51 Sathyan S, Pournami F, Prithvi AK, Nandakumar A, Prabhakar J, Jain N. Optimizing antibiotic use in culture-negative healthcare-associated infection with a 'stop' policy: a descriptive analytical study. J Trop Pediatr. 2022;69(1).

52 Sewornu R, Boakye-Yiadom E, Ativi E, Kwadzokpui PK, Senahey B, Owusu H, et al. Improved Utilisation and Quality of Blood Culture Services Following Operational Research in a Tertiary Hospital in Ghana. Tropical Medicine and Infectious Disease. 2025;10(9) (no pagination).

53 Boakye-Yiadom E, Najjemba R, Thekkur P, Labi AK, Gil-Cuesta J, Asafo-Adjei K, et al. Use and Quality of Blood Cultures for the Diagnosis of Bloodstream Infections: A Cross-Sectional Study in the Ho Teaching Hospital, Ghana, 2019-2021. Int J Environ Res Public Health. 2023;20(17).

54 Sowjanya SVNS, L V. Restriction of Antimicrobial Usage in a Tertiary Care Neonatal Unit in South India: A Before After Trial. International Journal of Neonatology. 2018;1(1):1–9.

55 Wang B, Li G, Jin F, Weng J, Peng Y, Dong S, et al. Effect of Weekly Antibiotic Round on Antibiotic Use in the Neonatal Intensive Care Unit as Antibiotic Stewardship Strategy. Front Pediatr. 2020;8:604244.

56 Liu XL, Yang J, Chen XH, Hua ZY. [Effects of antibiotic stewardship on neonatal bloodstream infections]. Zhongguo Dang Dai Er Ke Za Zhi. 2016;18(9):796–801.

57 Yin L, He L, Miao J, Yang W, Wang X, Ma J, et al. Actively surveillance and appropriate patients placements' contact isolation dramatically decreased Carbapenem-Resistant Enterobacteriaceae infection and colonization in pediatric patients in China. J Hosp Infect. 2020.

58 Zain J, Asim M, Firdos K, Laique T. Different Management Strategies for Term Newborns Delivered with Premature rupture of membranes. Pakistan Journal of Medical and Health Sciences. 2021;15(6):1423–5.

59 Sterne JAC, Savovic J, Page MJ, Elbers RG, Blencowe NS, Boutron I, et al. RoB 2: a revised tool for assessing risk of bias in randomised trials. BMJ. 2019;366:l4898.
60 Sterne JA, Hernan MA, Reeves BC, Savovic J, Berkman ND, Viswanathan M, et al. ROBINS-I: a tool for assessing risk of bias in non-randomised studies of interventions. BMJ. 2016;355:i4919.
61 National Institutes of Health (NIH) National Heart, Lung, and Blood Institute. Study quality assessment tools. 2021 [cited 2024 December 16]. Available from: https://www.nhlbi.nih.gov/health-topics/study-quality-assessment-tools.
62 GRADEpro. GRADEpro Guideline Development Tool [Software]. McMaster University and Evidence Prime; 2024.
63 WHO. Access, Watch, Reserve (AWaRe) classification of antibiotics for evaluation and monitoring of use: World Health Organization 2021 [Available from: https://iris.who.int/handle/10665/345555.
